# Manual joint mobilisation techniques, supervised physical activity, psychological treatment, acupuncture and patient education for patients with tension-type headache. A systematic review and meta-analysis

**DOI:** 10.1186/s10194-021-01298-4

**Published:** 2021-08-21

**Authors:** Lotte Skytte Krøll, Henriette Edemann Callesen, Louise Ninett Carlsen, Kirsten Birkefoss, Dagmar Beier, Henrik Wulff Christensen, Mette Jensen, Hanna Tómasdóttir, Hanne Würtzen, Christel Vesth Høst, Jakob Møller Hansen

**Affiliations:** 1 Department of Neurology, Danish Headache Centre, Rigshospitalet-Glostrup, University of Copenhagen, Valdemar Hansens Vej 5, 2600 Glostrup, Denmark; 2 Metodekonsulent Callesen, Åhavevej 26, 8600 Silkeborg, Denmark; 3 Danish Knowledge Centre on Headache Disorders, Rigshospitalet-Glostrup, University of Copenhagen, Valdemar Hansens Vej 5, 2600 Glostrup, Denmark; 4 Danish Health Authority, Islands Brygge 67, 2300 Copenhagen S, Denmark; 5 Department of Neurology, Odense University Hospital and University of Southern Denmark, Sdr. Boulevard 29, 5000 Odense C, Denmark; 6 Chiropractic Knowledge Hub, University of Southern Denmark, Campusvej 55, 5230 Odense M, Denmark; 7 Doktor Jensen Akupunkturklinik, Anders Billes Vej 2 B, 7000 Fredericia, Denmark; 8 Osteopath, Danske Osteopater and Q KLINIK, Finsensvej 42, 2000 Frederiksberg, Denmark; 9 The Multidisciplinary Pain Center (Section 7612), Rigshospitalet, Blegdamsvej 9, 2100 Copenhagen Ø, Denmark

**Keywords:** Headache, Exercise, Manual therapy, Mindfulness, Dry needling, Non-pharmacological treatment

## Abstract

**Background:**

Tension-type headache (TTH) has been ranked the second most prevalent health condition worldwide. Non-pharmacological treatments for TTH are widely used as a supplement or an alternative to medical treatment. However, the evidence for their effects are limited. Therefore, the aim of this study was to review the evidence for manual joint mobilisation techniques, supervised physical activity, psychological treatment, acupuncture and patient education as treatments for TTH on the effect of headache frequency and quality of life.

**Methods:**

A systematic literature search was conducted from February to July 2020 for clinical guidelines, systematic reviews, and individual randomised controlled trials (RCT). The primary outcomes measured were days with headache and quality of life at the end of treatment along with a number of secondary outcomes. Meta-analyses were performed on eligible RCTs and pooled estimates of effects were calculated using the random-effect model. The overall certainty of evidence was evaluated using the Grading of Recommendations, Assessment, Development, and Evaluation approach (GRADE). In addition, patient preferences were included in the evaluation.

**Results:**

In all, 13 RCTs were included. Acupuncture might have positive effects on both primary outcomes. Supervised physical activity might have a positive effect on pain intensity at the end of treatment and headache frequency at follow-up. Manual joint mobilisation techniques might have a positive effect on headache frequency and quality of life at follow-up. Psychological treatment might have a positive effect on stress symptoms at the end of treatment. No relevant RCTs were identified for patient education. The overall certainty of evidence was downgraded to low and very low. No serious adverse events were reported. A consensus recommendation was made for patient education and weak recommendations for the other interventions.

**Conclusion:**

Based on identified benefits, certainty of evidence, and patient preferences, manual joint mobilisation techniques, supervised physical activity, psychological treatment, acupuncture, and patient education can be considered as non-pharmacological treatment approaches for TTH. Some positive effects were shown on headache frequency, quality of life, pain intensity and stress symptoms. Few studies and low sample sizes posed a challenge in drawing solid conclusions. Therefore, high-quality RCTs are warranted.

**Supplementary Information:**

The online version contains supplementary material available at 10.1186/s10194-021-01298-4.

## Background

Headache was ranked third among causes of years lived with disability [1], and tension-type headache (TTH) was ranked the second most prevalent health condition worldwide in 2010 [2]. Current TTH has a global prevalence of 38% [3], and an estimated one-year prevalence from 38 to 87% [4–7]. Infrequent episodic TTH has a one-year prevalence of 19%, frequent episodic TTH 22%, and chronic TTH 3% [4]. Although the cause of TTH is unresolved, muscular factors, especially from pericranial muscles, stress and central sensitization all seem to play a role in the pathophysiology of TTH [8].

TTH has an economic impact on both society and the individual. The mean per-person annual indirect and direct costs was €303 which included lost productivity, absenteeism, outpatient care etc. The main direct annual costs was outpatient care (which accounted for 44% (€11) of the total direct annual costs) which amongst others included professional advice from a nurse, physiotherapist, osteopath, and chiropractor, followed by investigations, acute medications, hospitalisation and prophylactics [9].

Even though non-pharmacological treatments for TTH are widely used and the direct financial costs associated with non-pharmacological treatments are high, the evidence for their effects is limited. In 2010, a European expert panel recommended non-pharmacological treatment for TTH such as psychological approaches that included EMG biofeedback, cognitive-behavioural therapy and relaxation as well as physical therapy and acupuncture [10]. In 2012, The National Clinical Guideline Centre, commissioned by the National Institute for Health and Care Excellence (NICE), published a clinical guideline for the management of primary headache disorders (NICE CG150) [11]. The NICE CG150 found some evidence for acupuncture but found no evidence for manual therapy, exercise, psychological therapies, and education and self-management. Therefore, there is a need for a systematic evaluation of the latest research associated with these non-pharmacological approaches.

Non-pharmacological treatment should be considered for TTH management [10]. It can be considered as a supplement to medical treatment or as an alternative to medical treatment [12]. Therefore, non-pharmacological treatment may also be considered if TTH-patients suffer from unpleasant side effects and/or perceive the medical treatment as not effective.

Previous systematic reviews, published after the release of ICHD-2 [13] have evaluated the effectiveness of manual therapy compared to conservative or conventional treatments [14, 15], effectiveness of trigger point manual therapy [16], several modalities [17, 18] or a solely focus on quality of life [19]. We need a focus on modalities that used manual joint mobilisation techniques rather than including manual therapies as massage, trigger point therapy or cranio-sacral therapy.

To our knowledge, no previous systematic review has evaluated the literature for the evidence of physical activity for TTH such as strength training and aerobic exercises. Systematic reviews have evaluated the effect of exercises in combination with manual therapies [20] and of yoga exercise [21]. Yoga exercise showed potential positive effects on TTH frequency and pain intensity, however, a part of these yoga programmes included relaxation and meditation which may have had an independent effect.

Previous systematic reviews have evaluated the effect of psychological treatment on headache disorders [22–25]. However, these studies primarily reported results on headache rather than separately for TTH.

Acupuncture has shown a positive effect on TTH frequency [26, 27]. Though, we need a literature evaluation of the latest evidence for acupuncture excluding acupuncture with electrical stimulation as intervention and sham acupuncture with needle insertion as comparator.

Self-management programs have shown positive effects on headache such as pain intensity, mood and disability [28]. We need a literature search for programs on TTH that included disease specific education that also included education about medication in order to avoid medication-overuse headache.

With this review we aimed at gathering the evidence for these five areas to evaluate them in relation to the same outcomes in order to produce recommendations for a National Clinical Guideline (NCG). Which may facilitate the path for health professionals in identifying and directing the patient with TTH to the appropriate treatment strategy targeting the patient’s needs and resources.

Thus, the aim of the current study was to conduct a systematic review with meta-analyses and summarise the evidence for the following five separate treatment approaches for TTH: manual joint mobilisation techniques, supervised physical activity, psychological treatment, acupuncture, and patient education compared to other than the respective investigated modality on the effect of headache frequency and quality of life.

## Methods

The current review and NCG were based on the Population, Intervention, Comparison and Outcome (PICO) framework, with the methodology following the Grading of Recommendation, Assessment, Development, and Evaluation (GRADE) approach. This review adhere to the Preferred Reporting Items for Systematic Reviews and Meta-Analyses (PRISMA) statement and its protocol was registered in the International Prospective Register of Systematic Reviews (PROSPERO) in 2020, ID: CRD42020220124.

### Organization of the work

The Danish Knowledge Centre on Headache Disorders invited a multidisciplinary expert group with the purpose of developing a NCG for non-pharmacological treatment for patients with TTH as an aid to assist healthcare professionals and patients with TTH to identify and seek the most relevant non-pharmacological treatment strategies. The study process was led by the Danish Knowledge Centre on Headache Disorders (CVH, LNC, JMH) assisted by a specialist in literature search (KB) and a research methodologist (HEC). Members of the multidisciplinary expert group were appointed by Danish societies, i.e. the Chiropractor Association, the Osteopath Association, the Society for Physiotherapy, the Psychologist Association, the Medical Acupuncture, and the Neurological Society. Patient organizations and other representatives from the Danish health care system were included as a reference group. They provided feedback on the questions investigated, patient preferences, and on the final recommendations. The draft of the NCG was peer-reviewed by two external reviewers and disseminated for public feedback i.e. to relevant stakeholders.

### Clinical questions

Five PICO questions were constructed in order to investigate the effect of specific non-pharmacological interventions for patients diagnosed with TTH.

#### Population

Patients diagnosed with TTH according to the International Classification of Headache Disorder 2nd-3rd edition (ICHD 2–3) [13, 29] were included. The study population was aged 18 years or older. Studies of patients suffering from both TTH and migraine were also included but only if results were reported separately for TTH. We excluded studies of any other primary or secondary headache disorders. If studies of cervicogenic headache also included patients with TTH, there had to be a clear distinction between these two classifications based on ICHD 2–3 for both headache disorders and/or the classification proposed by Sjaastad et al. for cervicogenic headache [30].

#### Intervention

The investigated interventions included: 1) manual joint mobilisation techniques 2) supervised physical activity 3) psychological treatment 4) acupuncture and 5) patient education.

#### Comparator

1) Maunal joint mobilisation techniques compared to no manual joint mobilisation techniques, other treatment than manual joint mobilisation techniques, treatment as usual, placebo, sham intervention or waitlist; 2) Supervised physical activity compared to no supervised physical activity, other treatment than physical activity, treatment as usual, placebo, sham intervention or waitlist; 3) Psychological treatment compared to no psychological treatment, other treatment than psychological treatment, treatment as usual, placebo, sham intervention or waitlist; 4) Acupuncture compared to no acupuncture, other treatment than acupuncture, treatment as usual, placebo, sham intervention (studies that only used sham acupuncture with skin penentration as control group were excluded) or waitlist; 5) Patient education compared to no patient education, other treatment than patient education, treatment as usual, placebo, sham intervention or waitlist.

#### Outcomes

Two primary outcomes were selected for each PICO question: headache frequency in days per month and quality of life, both measured at the end of treatment. Additional primary outcomes were selected for psychological treatment (functioning) and patient education (degree of increased knowledge about illness and treament). A complete list of primary and secondary outcomes for each PICO question can be found in the Additional file 1 (Table 1).

### Definitions

#### Manual joint mobilisation techniques

Manual joint mobilisation techniques were defined as all manual techniques, mobilisation or manipulation within the normal range of motion of the joint, aimed at affecting the joints, muscles and connective tissues of the neck, chest and lower back.

#### Supervised physical activity

Supervised physical activity was defined as planned, repeated and structured physical activity [43].

#### Psychological treatment

Phychological treatment refers to approaches that address self-efficacy to decrease negative effect of reduced functioning and improve quality of life despite headache pain and headache related disability.

#### Acupuncture

Acupuncture was defined as a treatment where thin needles are inserted into the body including dry-needling and trigger point acupuncture excluding electroacupuncture.

#### Patient education

A disease-specific education aiming at improving the understanding of the disorder and treatment options, thus improving both knowledge and skills to master the life with recurring headache. Disease specific education can be conducted individually and in classes and also address family members containing information about the disease, its treatment, medication overuse, self-care, lifestyle, physical activity, regular diet and sleep. The intervention had to be conducted by a trained professional.

### Literature search and study selection

A systematic search was made on February 2020 to July 2020. Databases included EMBASE, PsycINFO, MEDLINE, CINAHL and PEDRO. The search was performed for Randomised Controlled Trials (RCT) included in existing guidelines and systematic reviews. Subsequently, an individual search for RCTs was performed, with the search date limited to the date of the latest search in identified existing systematic reviews. There was no restriction regarding publication status. The studies had to be published in English, Swedish, Norwegian or Danish. The search protocol can be found in Additional file 2.

The title and abstract of studies were independently screened by one review author followed by an assessment of full text of potential studies by two review authors. Any disagreement was recognised and resolved. The authors evaluating the studies were not blinded.

### Data extraction, risk of bias and quality assessment

Systematic reviews or existing clinical guidelines were identified and the methodological quality was evaluated using the AMSTAR [44] and AGREE [45] tools, respectively. Only systematic reviews and guidelines of sufficient quality were included, followed by data extraction of individual RCTs mentioned in the review and/or clinical guideline. Data including population demographics, intervention and control details, outcome and time measurement were independently extracted, by two review authors, from relevant RCTs. Risk of bias within individual RCTs was independently assessed by two review authors using the Cochrane risk of bias tool [46]. Any discrepancy was resolved through discussion. The review authors were not blinded.

### Summary measures

Meta-analysis for each outcome were performed if data, in the included RCTs, was comparable. Data was extracted in the online software program, Covidence [47] and subsequently exported to RevMan5 [48], where pooled estimates of effects were calculated using the random-effect model. Dichotomous outcomes were calculated as relative risks (RR) and continous outcomes as mean differences (MD). If different scales were used in the measurement of continuous outcomes, a standardised mean difference (SMD) was applied. A 95% confidence interval was calculated for both dichotomous and continuous outcomes. I^2^ statistics were used for quantification of statistical heterogeneity (I^2^ > 50% was considered substantial heterogeneity). There was insufficient data to allow for any subgroup analysis. Results were displayed in forest plots for each outcome.

### Certainty of evidence and recommendations

The GRADE approach was applied to assess the certainty of evidence obtained for each outcome. One of four possible rating was possible; high, moderate, low or very low. If needed, downgrading the certainty of evidence was done based on the extent of risk of bias, inconsistency, indirectness, imprecision, and publication bias. The overall certainty of evidence for each clinical question was based on the lowest rating of the primary outcome.

If evidence was available for a given clinical question, either a strong or weak recommendation, for or against an intervention was made. If no evidence was available, a consensus recommendation was made. Recommendations were based on a combined evaluation of the certainty of evidence, patient preferences, and the identified benefits and harms.

### Patient preferences and consensus recommendation

The Danish Knowledge Centre on Headache Disorders performed a survey on patients’ expectations concerning treatments offered by chiropractors, physiotherapists, psychologists, acupuncturists and courses about knowledge on illness and treatment. They were also asked whether they would seek this treatment again. The headache population was recruited by a modified snowball sampling via facebook and patient organizations.

Patient preferences and consensus recommendation were based on direct patient involvement based on responses from the survey, direct feedback from chairmen (ABO, BOL) and board member (JB) representing Danish Migraine Association, Denmark’s Patient Society for People with Headache, and the Migraine and Headache Society, respectively. Additionally, patient preferences were based on expert opinion of the multidisciplinary working group formed by their experience in working with patients in daily practice.

## Results

### Literature search and study selection

**Fig. 1:**
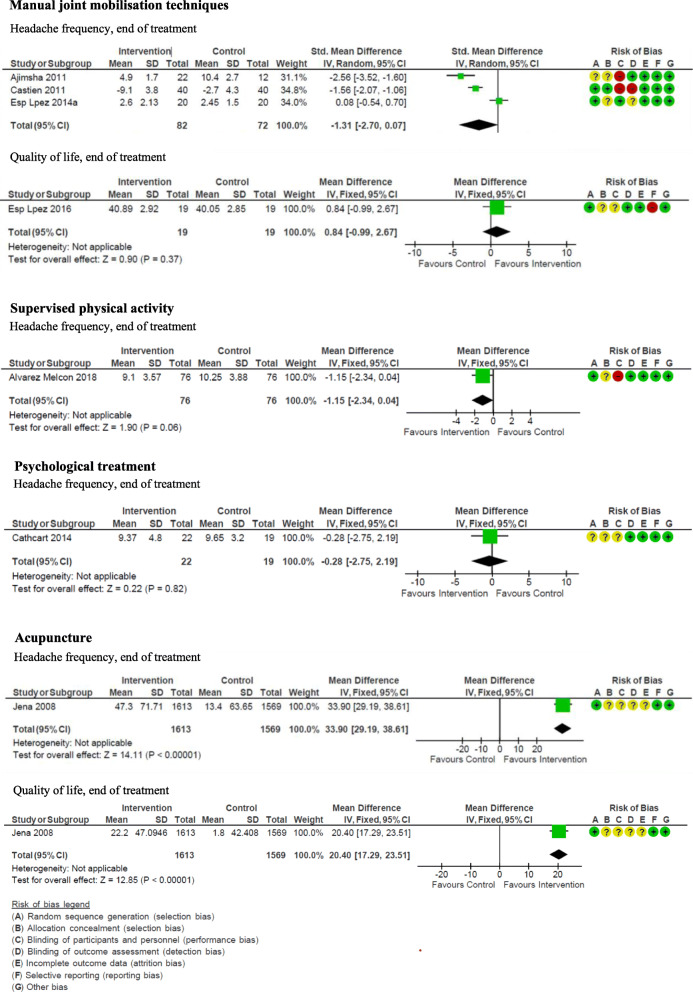
Non-pharmacological treatments for tension-type headache. Meta-analyses of primary outcomes: headache frequency and quality of life at end of treatment of manual joint mobilisation techniques, supervised physical activity, psychological treatment, and acupuncture. No randomized controlled trials were found for patient education. SD: standard deviation. CI: confidence interval

One relevant guideline was identified [11] and considered of high methodological quality in accordance with the AGREE-II tool. From this guideline one relevant RCT [32], concerning manual joint mobilisation techniques, was identified. This was followed by a search for existing systematic reviews; 11 relevant reviews were identified [14, 16, 17, 19, 22–27, 49], and rated using the AMSTAR tool. From the included systematic reviews, ten relevant RCTs were identified (six concerning manual joint mobilisation techniques [31–36]; three concerning psychological treatment [39–41]; one concerning acupuncture [42]. The AGREE-II and AMSTAR assessments can be found in Additional file 1 (Figure 1 and Table 2). An individual search for RCTs identified two additional studies, both concerning supervised physical activity [37, 38]. PRISMA flow charts of identified clinical guidelines, systematic reviews and primary studies can be seen in Additional file 1 (Figures 2–12).

### Estimated effect and recommendations

The RCTs included for each PICO and the outcomes assessed can be found in Table 1**.** The meta-analyses of primary outcomes can be seen in Fig. 1. Meta-analyses of secondary outcomes can be found in Additional file 1 (Figures 13–16). An overview of the recommendations can be found in Table 2.

**Table 1:** Identified randomised controlled trials (RCTs) for non-pharmacological treatments for tension-type headache. Studies were found in existing guidelines and systematic reviews, or via a search for individual RCTs

Non-pharmacological treatment	Number of RCTs identified	Intervention	Comparison	Outcomes assessed in the studies
Manual joint mobilisation techniques	6 (Ajimsha et al. [31]); Castien et al. [32]; Espi-Lopez et al. 2014a [33]; Espi-Lopez et al. 2014b [34]; Espi-Lopez et al. 2016 [35]; Rolle et al. [36])	Direct myofascial release technique (Ajimsha et al.), manual therapy (Castien et al.), osteopathic manual therapy (Rolle et al.), suboccipital muscle manipulation (Espi-Lopez 2014a, 2014b, 2016).	Sham intervention (Ajimsha et al.; Rolle et al.), treatment as usual (Castien et al.), no treatment (Espi-Lopez 2014a, 2014b, 2016).	• Headache frequency (at the end of treatment and at follow-up) • Quality of life (at the end of treatment and at follow-up) • Headache intensity • Serious adverse events
Supervised physical activity	2 (Alvarez-Melcon et al .[37]; Andersen et al. [38])	Training program for the neck and relaxation (Alvarez-Melcon et al.) and training of neck and shoulder using an elastic band (Andersen et al.).	Relaxation (Alvarez-Melcon et al.) and weekly information on health (Andersen et al).	• Headache frequency (at the end of treatment and at follow-up) • Headache intensity • The use of attack-medicine/analgesics
Psychological treatment	3 (Cathcart et al. [39]; Holroyd et al. [40]; Omidi et al. [41])	Mindfulness-based therapy (Cathcart et al.), cognitive behavioural stress management (Holroyd et al.) and mindfulness-based stress reduction (Omidi et al.).	Waiting list (Cathcart et al.), placebo (Holroyd et al.) and medical treatment as usual (Omidi et al.).	• Headache frequency • Stress symptoms
Acupuncture	1 (Jena et al. [42])	Acupuncture	No treatment	• Quality of life • Number of days with migraine • Serious adverse events
Patient education	None identified	–	–	–

**Table 2:** Overview of the recommendations for the use of non-pharmacological treatments for patients with tension-type headache

Intervention	Certainty of evidence	Patient preferences	Recommendation
Manual joint mobilisation techniques	Very Low	Variability in patient preferences is expected	Weak recommendation for
Supervised physical activity	Very low	No substantial variability in patient preferences is expected	Weak recommendation for
Psychological treatment	Very low	Variability in patient preferences is expected	Weak recommendation for
Acupuncture	Low	Variability in patient preferences is expected	Weak recommendation for
Patient education	None identified	No substantial variability in patient preferences is expected	Consensus based recommendation

#### Manual joint mobilisation techniques

Six relevant RCTs were identified [31–36]. In the study by Ajimsha et al. [31], direct myofascial release and sham intervention were included in the meta-analysis. In the studies by Espi-Lopez et al. [33–35], suboccipital muscle manipulation and no treatment were included in the meta-analysis. In the study by Rolle et al. [36], data could not be included in the analysis as data were reported visually in figures.

Results showed that manual joint mobilisation techniques have a potential positive effect on headache frequency (SMD -1.31 (95% CI -2.70 - 0.07)) and quality of life (MD 0.84 (95% CI -0.09 - 2.67)) when measured at follow-up. The certainty of the evidence was very low due to risk of bias, inconsistency and imprecision. We expect that there would be variability in the number of patients who would prefer this intervention. Based on these findings, a weak recommendation is made for manual joint mobilisation techniques which can be considered as a supplement to medical treatment for patients with TTH.

#### Supervised physical activity

Two relevant RCTs were identified [37, 38]. In the study by Andersen et al. [38], data were not presented in a form that could be used in the meta-analysis.

Results showed a potential positive effect of headache intensity at the end of treatment (MD -0.61 (95% CI -1.09 - -0.13)) and headache frequency at follow-up (MD -1.15 (95% CI -2.34 - 0.04)). The certainty of evidence was very low due to risk of bias and imprecision. We expect that most patients would prefer this intervention. Based on these findings, a weak recommendation is made for supervised physical activity which can be considered as a supplement to medical treatment for patients with TTH.

#### Psychological treatment

Three relevant RCTs were identified [39–41]. In the study by Holroyd et al. [40], the data could not be included in the meta-analysis as they were depicted visually in figures.

Results showed that psychological treatment potentially has a positive effect on stress symptoms at the end of treatment (MD -0.28 (95% CI -2.75 - 2.19)). The certainty of evidence was very low due to risk of bias and imprecision. We expect that there would be variability in the number of patients who would prefer this intervention. Based on these findings, a weak recommendation is made for psychological treatment which can be considered as a supplement to medical treatment for patients with TTH.

#### Acupuncture

One relevant RCT was identified [42]. Results showed that acupuncture potentially has a positive effect on headache frequency (MD 33.90 (95% CI 29.19–38.61)) and quality of life (MD 20.40 (95% CI 17.29–23.51)) at the end of treatment. The certainty of evidence was low due to risk of bias and imprecision. We expect that there would be variability in the number of patients with TTH who would prefer this intervention. Based on these findings, a weak recommendation is made for acupuncture which can be considered as a supplement to medical treatment for patients with TTH.

#### Patient education

No relevant RCTs were identified, and therefore the recommendation is solely based on consensus from experts. Based on clinical experience, patient education provided as systematic information and counseling, may have a positive effect on the patient’s ability to manage their disease and their life with chronic illness thereby reducing the overall symptom burden. We expect that the majority of patients would like to receive this intervention. Based on consensus, it is recommended to consider using patient education as a supplement to medical treatment for patients with TTH.

No serious adverse events were identified for any of the non-pharmacological treatment approaches.

#### Survey of patient expectations

The survey was answered by 380 patients. Regarding the different intervention, the largest part of patients expected reduced number of headache days (chiropractor 72%, physiotherapist 71%, psychologist 12%, acupuncture 79%, and education (lectures/ classes) 9% respectively). Subsequently, improved quality of life (chiropractor 46%, physiotherapist 47%, psychologist 69%, acupuncture 40%, education (lectures/classes) 44% respectively), and reduced use of analgesics (chiropractor 43% physiotherapist 41%, psychologist 6%, acupuncture 47%, and education (lectures/classes) 10% respectively). Moreover, 86% of the patients expected education to result in increased knowledge about headache and headache treatment. Psychological treatment was expected to result in improved pain coping strategies (63%) and positive impact on depression, anxiety and stress (58%). Regarding the likelihood with which patients would ask for a specific intervention again, there was a certain amount of variability relating to interventions by chiropractors, physiotherapists, acupuncture and psychologists, whereas 54% of the patients would ask for education again. Additional file 1 (Figure 17).

## Discussion

The current study presented a systematic review of the evidence for manual joint mobilisation techniques, supervised physical activity, psychological treatment, acupuncture, and patient education as non-pharmacological treatment strategies for TTH regarding headache related outcomes, quality of life and disability.

The findings reveal that non-pharmacological treatment approaches for TTH were safe and showed a number of positive effects. In summary, in relation to primary outcomes, a potential positive effect on headache frequency and quality of life at the end of treatment were found for acupuncture. In relation to secondary outcomes, a potential positive effect was found for headache frequency (manual joint mobilisation techniques and supervised physical activity) and quality of life (manual joint mobilisation techniques) at follow-up; pain intensity (supervised physical activity) and stress symptoms (psychological treatment) at the end of treatment.

According to the GRADE approach, there was very low certainty of evidence for supervised physical activity and psychological treatment owing to risk of bias and imprecision. There was very low certainty of evidence for manual joint mobilisation techniques owing to risk of bias, inconsistency and imprecision. There was low certainty of evidence for acupuncture owing to risk of bias and imprecision. No relevant RCTs were found regarding patient education. There were no identified serious adverse events for any of the investigated interventions. A consensus recommendation was made for patient education and weak recommendations for the other interventions.

To the best of our knowledge, no former guideline, applying a similar systemtic approach, has been publised since the NICE CG150 was publiched in 2012. In line with the current review, the NICE CG150 [11] reviewed the evidence for manual therapies, exercise, psychological therapies, acupuncture, and patient education and self-management also applying the GRADE approach. There are similarities between the findings from the NICE CG150 and the current review. The NICE CG150 found some evidence for acupuncture as preventive treatment for TTH and made a recommendation for this treatment. We based our recommendation on one large RCT which was rated with low certainty of evidence. The NICE CG150 found low to very low certainty of evidence for manual therapy, but did not find enough evidence to form a recommendation. We formed a recommendation; however, the overall certainty of evidence was also rated very low. In contrast to the current review, the NICE CG150 did not find any relevant RCTs evaluating the effect of exercise on TTH. We based our recommendation on one study that could be included in the meta-analysis. The included study was based on university students with a mean age of 20 years, limiting the external validity. In line with the NICE CG150, that found low to very low certainty of evidence for psychological treatment but were not able to form a recommendation. We formed a recommendation but found an overall certainty of evidence that also was rated very low. In line with our review, the NICE CG150 did not find any evidence for self-management and educational programs for TTH.

Overall, direct comparison with other systematic reviews is challenging due to methodological variations, especially regarding variability in the intervention and comparison groupings as well as grouping in the analyses. However, in line with our results, earlier reviews reported potential positive effects for manual therapies on headache frequency [16, 18] and quality of life [14, 19]. All studies found low to very low level of evidence or inconclusive results [14–19]. However, these studies represent a variety of different treatment modalities. We included studies which directed the treatment towards affecting the joints, muscles and connective tissues of the neck, chest and lower back.

No previous systematic reviews were identified evaluating the effect of physical activity for TTH. We identified only RCT studies that evaluated the effect of exercises such as strength training, but no studies were identified evaluating aerobic exercise. Therefore, future studies may investigate the effect of a combination of these exercise treatments.

Regarding psychological treatment, Lee et al. (2019) [25] reported in a subgroup analysis that patients with TTH showed positive effects on headache index (reflecting diverse aspects of headache suffering [25]). We found positive effects on stress symptoms, which may indicate that this treatment approach could contribute to positive effects on both headache and psychological symptoms.

In line with previous systematic reviews on acupuncture [26, 27], we found positive effects on headache frequency. We excluded studies using electroacupuncture as intervention and studies using sham acupuncture with needle insertion and identified only one RCT study. Therefore, further studies using these criteria are needed to confirm these results.

There is a major gap in the quality of research related to patient education. Ashina et al. (2021) [12] indicated that all patients with TTH benefit from education that includes healthy lifestyle habits. Patient education aims to provide patients, who experience an impaired quality of life, with a better understanding and knowledge about their disease together with strategies for self-care and self-management. Based on clinical experience, the multidisciplinary expert group of this review recommended patient education for patients with frequent and chronic TTH as they often seek knowledge about their disorder and tools to manage their life with chronic illness. However, there is a need for more research in various programs within the field of headache.

The recommendations from the current review were also formed by an assessment of patient preferences with respect to non-pharmacological treatment. There may be variations in individuals’ need for treatment depending on the degree of headache and the impact on their daily living and quality of life. The patients with TTH should be informed, by healthcare professionals, about options for relevant forms of treatment and about realistic expectations of their effects. Lack of possibility of reimbursement may limit some patients’ access to non-pharmacological treament. Ideally, non-pharmacological treatments should be subjected to cost-effectiveness analysis in future guidelines.

The internal validity of the included studies needs to be addressed. In several studies, the reporting of headache classification was inconsistent and unclear. It was not clear who diagnosed the participants and how their diagnosis was obtained even if it was stated that the diagnosis was based on ICHD criteria. Currently, there are validated tools to classify primary headaches, however, there is a lack of tools that can be used by non-experts in primary care settings [50]. It is recommended to aid the diagnosis of headache by keeping a headache diary [11, 29].

Since the published guideline from the NICE CG150 in 2012 and updates in 2015, we found few studies matching each PICO. As a result, further research is warranted. It is recommended that future studies use uniform outcome measures and follow the designs of clinical trials as recommended by the guidelines from the International Headache Society [51], and follow the CONSORT statement for reporting randomised controlled trials [52].

The overall certainty of evidence for the included studies assessed by using GRADE was low to very low owing to insufficient blinding of staff, participants and outcome assessors. Moreover, there were uncertainties about the random sequence generation and allocation concealment in some studies, which could skew the baseline parameters in the intervention arms. The effect estimates display imprecision mainly because of wide confidence intervals, few studies, and low sample sizes. Consequently, is it difficult to draw any solid conclusions. Future reviews may, therefore, come to different conclusions than the conclusions reported from our review.

### Strengths and limitations

We have based our review on the best current standards for systematic reviews and meta-analyses which strengthens the resulting NCG. The work was based on the PICO framework and performed using transparent quality assessment instruments, the AGREE-II and AMSTAR tools for clinical guidelines and systematic reviews respectively. The Cochrane risk of bias tool was used for individual RCTs and the overall certainty of evidence was performed using the GRADE approach. The reporting of this review was based on the PRISMA statement and registered beforehand in the PROSPERO register. The multidisciplinary expert group was composed of relevant experts with both clinical and research experience, and the literature search was performed by an expert research librarian. Finally, this NCG was peer-reviewed by the professional and medical societies and by two independent and international experts.

There were some limitations of this review. The GRADE approach is prone to downgrade research that is not double-blinded, which is challenging for clinical non-pharmacological research. The literature search was limited to English and Scandinavian languages. The survey on headache patients’ expectations were based on a modified snowball sampling, therefore this population may not represent the total headache population. The aim of the survey was to have an overview of the use and preferences of non-pharmacological treatment as a starting point for this review. The target population was headache patients. Therefore, there were no distinction between headache types, headache frequency, demographics or psychiatric or other comorbidities that may have influenced the responses. The data presented in the meta-analyses are based on the published material. The authors of the included studies have not been contacted for further details. This review and recommendations are primarily based on an evaluation of the certainty of evidence in combination with consensus between the members of the multidisciplinary expert group, patient preferences, and benefits and harms.

## Conclusion

Based on the identified benefits, the certainty of evidence, and patient preferences, the multidisciplinary expert group of this review concluded that manual joint mobilisation techniques, supervised physical activity, psychological treatment, acupuncture, and patient education can be considered as treatment approaches for TTH. No serious adverse events were identified for any of the included studies. Some positive effects were shown on headache frequency, quality of life, pain intensity and stress symptoms. Few studies and low sample sizes posed a challenge in drawing solid conclusions. Therefore, there is a need for high-quality RCTs in this field.

This NCG can be used by healthcare professionals as an aid in identifying and directing the patient with TTH to the appropriate treatment strategy targeting the patient’s needs and resources based on evidence.

## Supplementary Information

**Additional file 1.** Additional tables and figures. Complete list of primary and secondary outcomes, AGREE and AMSTAR evaluations, flowcharts of litterature search and meta-analyses of secondary outcomes.

**Additional file 2.** Search protocol.

## Data Availability

The datasets generated and analysed during the current study are available at the Danish Knowledge Centre on Headache Disorders webpage: https://videnscenterforhovedpine.dk/nkr/

## Abbreviations

DHA: Danish Health Authority
ICHD: International Classification of Headache Disorders
GRADE: Grading of Recommendation, Assessment, Development, and Evaluation
MD: Mean Differences
NCG: National Clinical Guideline
NICE: National Institute for Health and Care Excellence
PICO: Population, Intervention, Comparison and Outcome
RCT: Randomised Clinical Trial
RR: Relative Risk
TTH: Tension-type Headache
