# Correction to: Manual joint mobilisation techniques, supervised physical activity, psychological treatment, acupuncture and patient education for patients with tension-type headache. A systematic review and meta-analysis

**DOI:** 10.1186/s10194-021-01329-0

**Published:** 2021-10-06

**Authors:** Lotte Skytte Krøll, Henriette Edemann Callesen, Louise Ninett Carlsen, Kirsten Birkefoss, Dagmar Beier, Henrik Wulff Christensen, Mette Jensen, Hanna Tómasdóttir, Hanne Würtzen, Christel Vesth Høst, Jakob Møller Hansen

**Affiliations:** 1 Department of Neurology, Danish Headache Centre, Rigshospitalet-Glostrup, University of Copenhagen, Valdemar Hansens Vej 5, 2600 Glostrup, Denmark; 2 Metodekonsulent Callesen, Åhavevej 26, 8600 Silkeborg, Denmark; 3 Danish Knowledge Centre on Headache Disorders, Rigshospitalet-Glostrup, University of Copenhagen, Valdemar Hansens Vej 5, 2600 Glostrup, Denmark; 4 Danish Health Authority, Islands Brygge 67, 2300 Copenhagen S, Denmark; 5 Department of Neurology, Odense University Hospital and University of Southern Denmark, Sdr. Boulevard 29, 5000 Odense C, Denmark; 6 Chiropractic Knowledge Hub, University of Southern Denmark, Campusvej 55, 5230 Odense M, Denmark; 7 Doktor Jensen Akupunkturklinik, Anders Billes Vej 2 B, 7000 Fredericia, Denmark; 8 Osteopath, Danske Osteopater and Q KLINIK, Finsensvej 42, 2000 Frederiksberg, Denmark; 9 The Multidisciplinary Pain Center (Section 7612), Rigshospitalet, Blegdamsvej 9, 2100 Copenhagen Ø, Denmark

**Correction to: J Headache Pain 22, 96 (2021)**

**https://doi.org/10.1186/s10194-021-01298-4**

Following the publication of the original article [1], we were notified that the quality for Fig. 1 needed improvement. The presentation of the data in Fig. 1 has not changed.

The original article has been corrected.
