# Supplementary material for: Manual joint mobilisation techniques, supervised physical activity, psychological treatment, acupuncture and patient education for patients with tension-type headache. A systematic review and meta-analysis
Source: J Headache Pain. 2021 Aug 21;22(1):96. doi: 10.1186/s10194-021-01298-4 (PMC8379845; doi:10.1186/s10194-021-01298-4)
Supplement: Supplementary file 1 — Additional file 1. Additional tables and figures. Complete list of primary and secondary outcomes, AGREE and AMSTAR evaluations, flowcharts of litterature search and meta-analyses of secondary outcomes. [file 10194_2021_1298_MOESM1_ESM.pdf]

Additional tables and figures

Manual joint mobilisation techniques, supervised physical activity, psychological treatment, acupuncture and patient education for patients with tension-type headache. A systematic review and meta-analysis

Lotte Skytte Krøll[1], Henriette Edemann Callesen[2], Louise Ninett Carlsen[1,3], Kirsten Birkefoss[4], Dagmar Beier[5], Henrik Wulff Christensen[6], Mette Jensen[7], Hanna Tómasdóttir[8], Hanne Würtzen[9], Christel Vesth Høst[3], Jakob Møller Hansen[1,3].

Table 1 Primary and secondary outcomes for, manual joint mobilisation techniques, supervised physical activity, psychological treatment, acupuncture, and patient education.

Interventions	Outcome	Time-point	Primary/secondary
• Manual joint mobilisation techniques<br>• Supervised physical activity<br>• Acupuncture	Headache frequency, days per month	At the end of treatment	Primary
	Quality of life	At the end of the treatment	Primary
	Headache frequency, days per month	Follow-up after the end of treatment	Secondary
	Quality of life	Follow-up after the end of treatment	Secondary
	Headache intensity measured on Numeric rating scale or 100mm VAS	At the end of the treatment	Secondary
	Frequency of tension-type headache, days per month	At the end of the treatment	Secondary
	Functioning	At the end of the treatment	Secondary
	Acute medication intake in days	At the end of the treatment	Secondary
	Sick leave, days per month	At the end of the treatment	Secondary
	Serious adverse events	At the end of the treatment	Secondary
• Psychological treatment	Headache frequency, days per month	At the end of the treatment	Primary
	Quality of life	At the end of the treatment	Primary
	Functioning	At the end of the treatment	Primary
	Headache intensity measured on Numeric rating scale or 100mm VAS	At the end of the treatment	Secondary
	Compliance	At the end of the treatment	Secondary
	Frequency of tension-type headache, days per month	At the end of the treatment	Secondary
	Functioning	Follow-up after the end of treatment	Secondary
	Depression	At the end of the treatment	Secondary
	Anxiety	At the end of the treatment	Secondary
	Stress symptoms	At the end of the treatment	Secondary
	Serious adverse events	At the end of the treatment	Secondary
• Patient education	Headache frequency, days per month	At the end of the treatment	Primary
	Quality of life	At the end of the treatment	Primary
	Degree of increased knowledge about illness and treament	At the end of the treatment	Primary
	Frequency of tension-type headache, days per month	At the end of the treatment	Secondary
	Headache intensity measured on Numeric rating scale or 100mm VAS	At the end of the treatment	Secondary
	Self-rated health	At the end of the treatment	Secondary
	General pain	At the end of the treatment	Secondary
	Burden of symptoms	At the end of the treatment	Secondary
	Serious adverse events	At the end of the treatment	Secondary

Evaluation of the guideline: National Institute for Health and Care Excellence. "Headaches in over 12s: diagnosis and management [CG150]." (2012).

Domain 1. Scope and Purpose		
	Appraiser 1	Appraiser 2
Item 1	6	5
Item 2	5	6
Item 3	7	3

Domain 2. Stakeholder Involvement		
	Appraiser 1	Appraiser 2
Item 4	4	4
Item 5	5	6
Item 6	5	4

Domain 3. Rigour of Development		
	Appraiser 1	Appraiser 2
Item 7	3	6
Item 8	7	6
Item 9	6	6
Item 10	5	6
Item 11	7	6
Item 12	6	6
Item 13	2	1
Item 14	5	4

Domain 4. Clarity of Presentation		
	Appraiser 1	Appraiser 2
Item 15	6	6
Item 16	7	4
Item 17	7	6

Domain 5. Applicability		
	Appraiser 1	Appraiser 2
Item 18	2	4
Item 19	1	3
Item 20	7	5
Item 21	7	2

Domain 6. Editorial Independence		
	Appraiser 1	Appraiser 2
Item 22	7	3
Item 23	4	4

Overall Assessment		
	Appraiser 1	Appraiser 2
OA1	5	5

Fig. 1 AGREE evaluation

Table 2 AMSTAR Evaluation

Name	Was meta-analysis performed for the relevant outcomes?	1. Was an 'a priori' design provided?	2. Was there duplicate study selection and data extraction?	3. Was a comprehensive literature search performed?	4. Was the status of publication (i.e. grey literature) used as an inclusion criterion?	5. Was a list of studies (included and excluded) provided?	6. Were the characteristics of the included studies provided?	7. Was the scientific quality of the included studies assessed and documented?	8. Was the scientific quality of the included studies used appropriately in formulating conclusions?	9. Were the methods used to combine the findings of studies appropriate?	10. Was the likelihood of publication bias assessed?	11. Was the conflict of interest included?
Cerritelli et al. 2017	No	No	Yes	Yes	Yes	Can't answer	Yes	Yes	Yes	Yes	Yes	Yes
Falsiroli Maistrello et al. 2019	Yes	Yes	Yes	Yes	No	No	Yes	Yes	Yes	Yes	Yes	No
Coelho et al. 2019	Yes	Yes	No	Yes	No	No	Yes	Yes	Yes	Yes	Yes	Yes
Falsiroli Maistrello et al. 2018	Yes	Yes	Yes	Yes	No	No	Yes	Yes	Yes	Yes	Yes	No
Anheyer et al. 2019	Yes	No	Yes	Yes	No	Yes	Yes	Yes	Yes	Yes	Yes	Yes
Gu et al. 2018	Yes	No	No	Yes	No	No	Yes	Yes	Yes	Yes	Yes	Yes
Harris et al. 2015	No	No	Yes	Yes	No	Can't answer	Yes	Yes	Yes	No	No	Yes
Lee et al. 2019	Yes	No	No	Yes	No	No	Yes	Yes	Yes	Yes	Yes	Yes
Linde et al. 2016	Yes	No	Yes	Yes	Yes	No	Yes	Yes	Yes	Yes	Yes	No
France et al. 2014	No	No	Yes	Yes	No	No	Yes	Yes	No	No	No	Yes

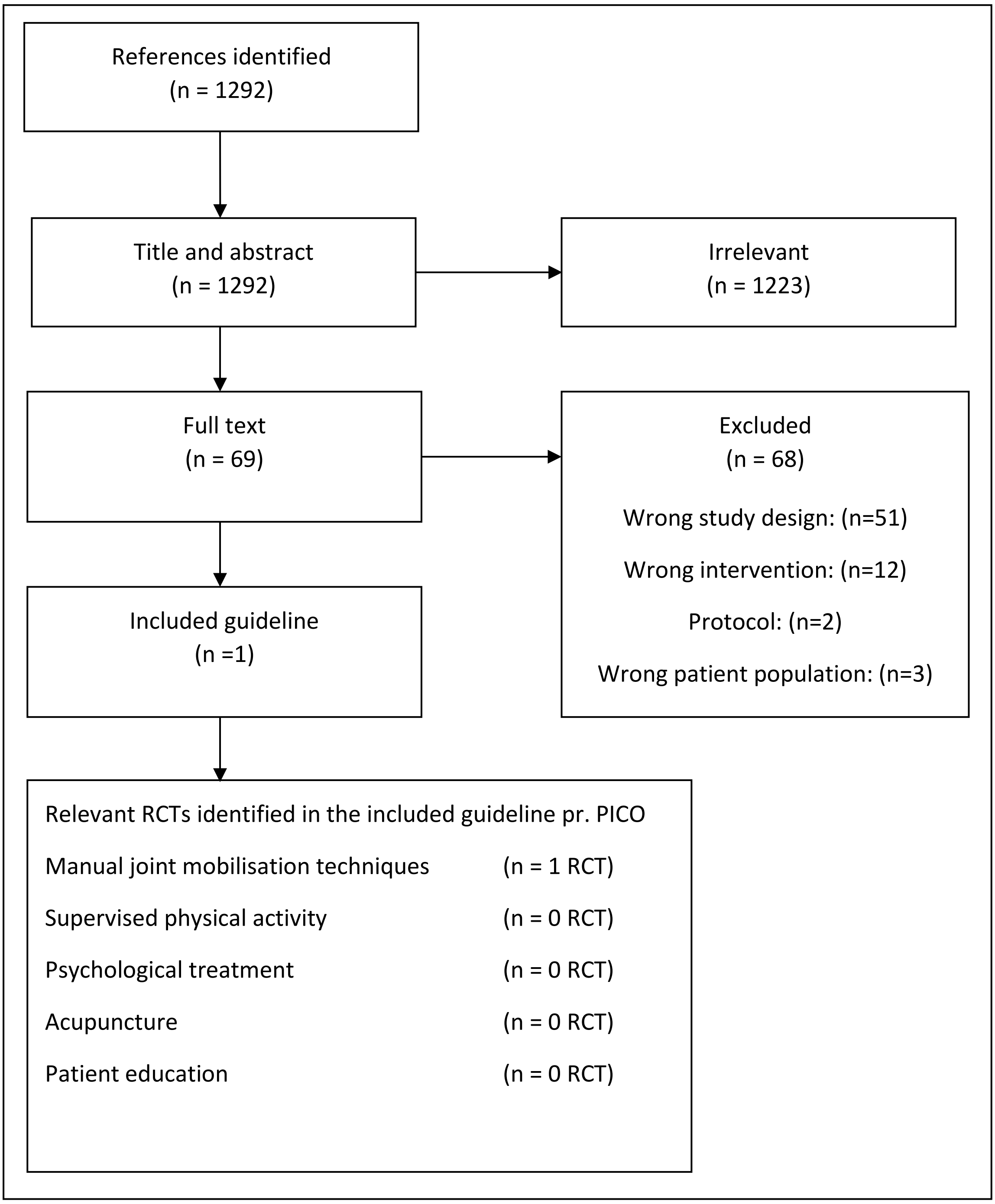

Fig. 2 Flowchart, search for guidelines.

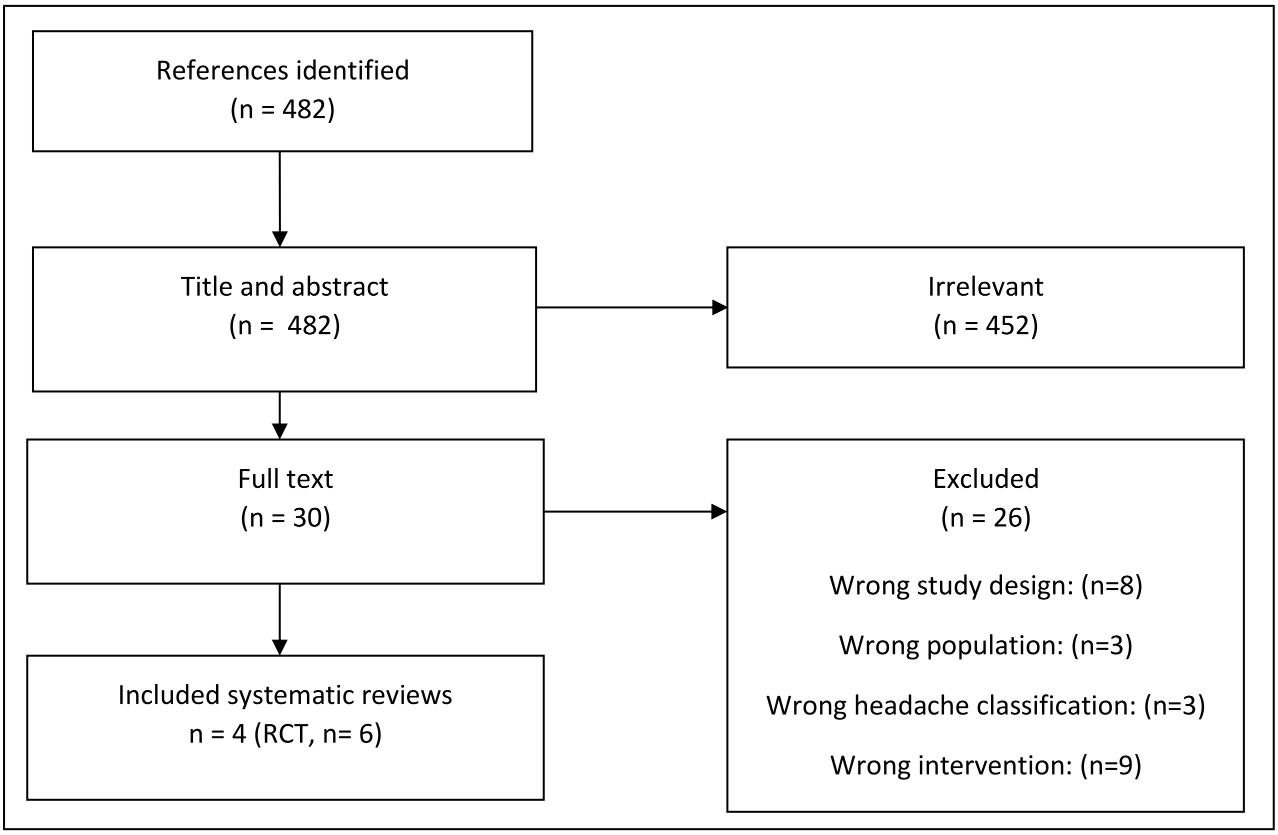

Fig. 3 Flowchart, search for systematic reviews, manual joint mobilisation techniques.

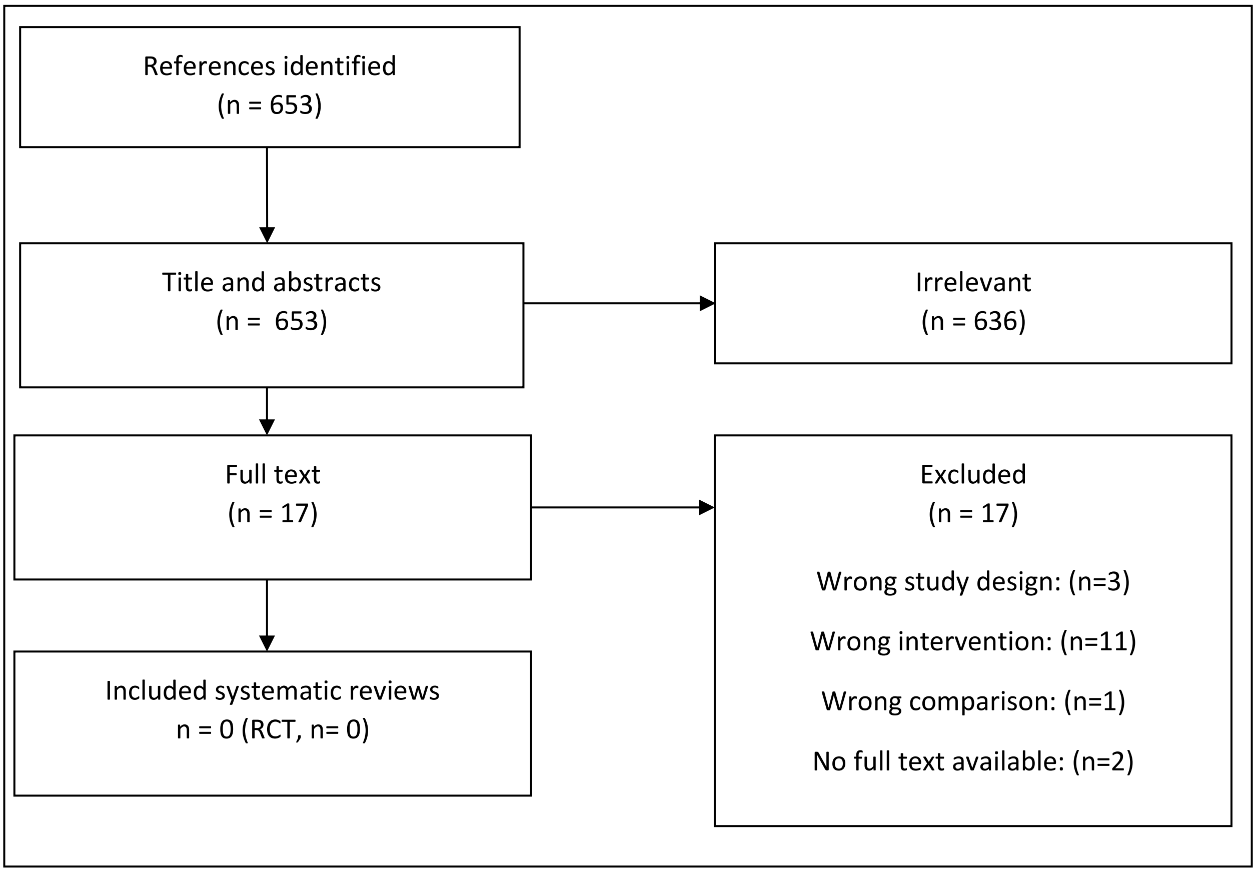

Fig. 4 Flowchart, search for systematic reviews, supervised physical activity.

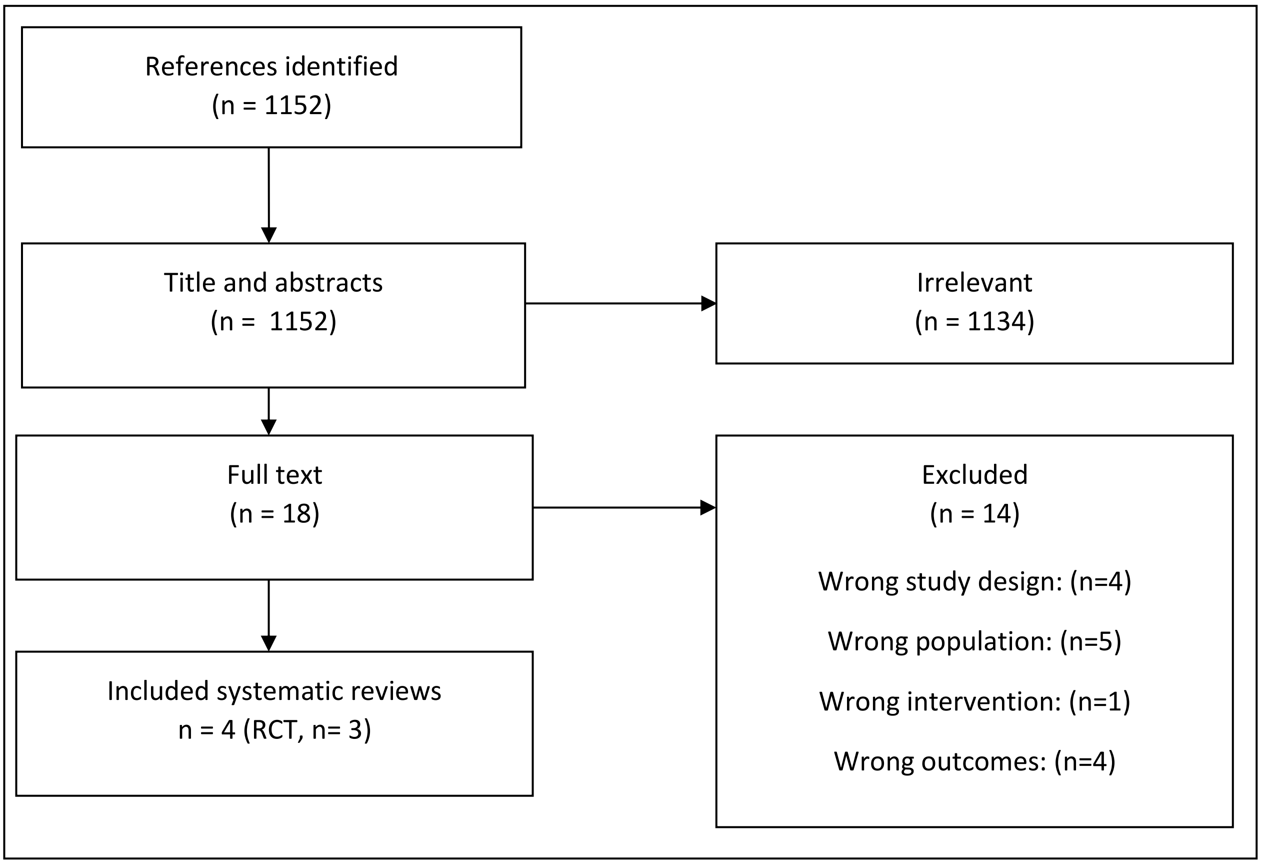

Fig. 5 Flowchart, search for systematic reviews, psychological treatment.

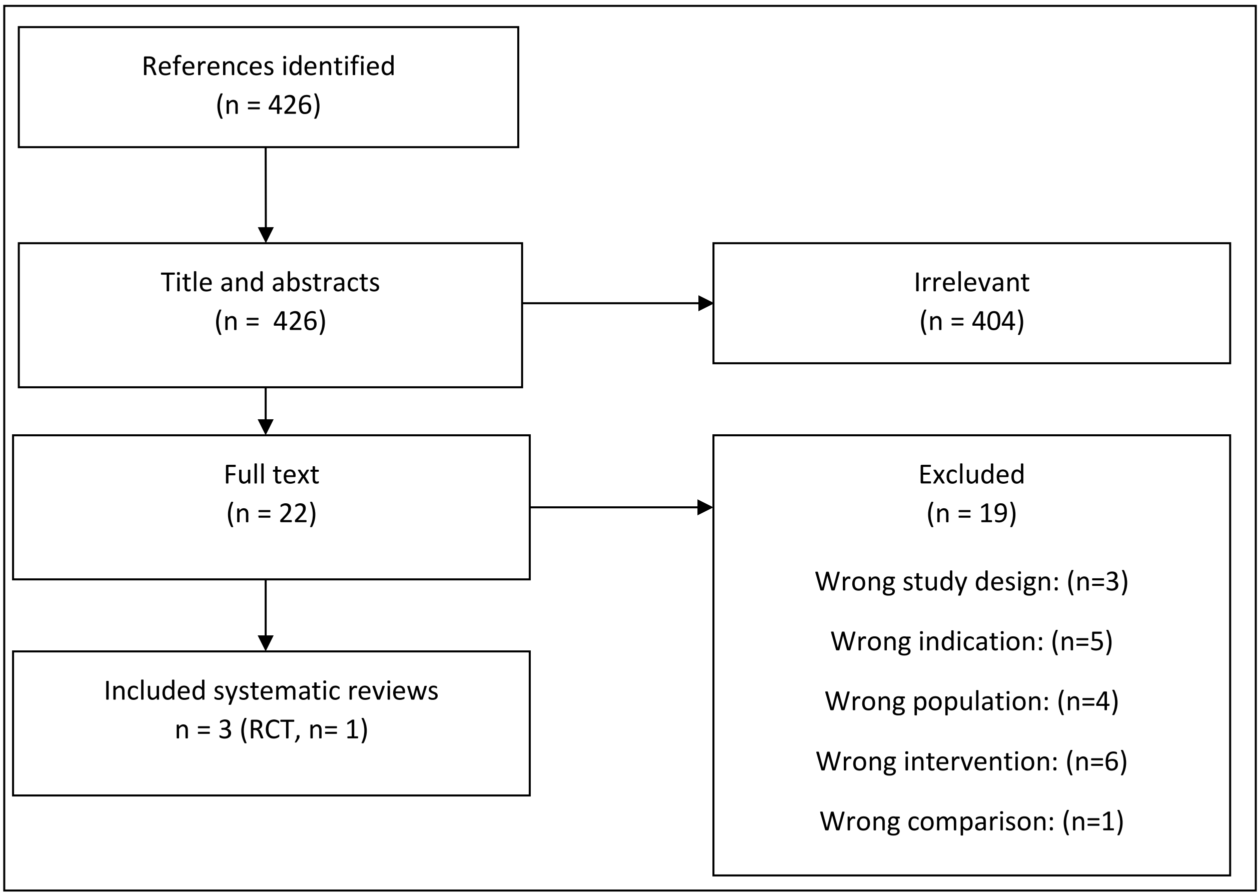

Fig. 6 Flowchart, search for systematic reviews, acupuncture.

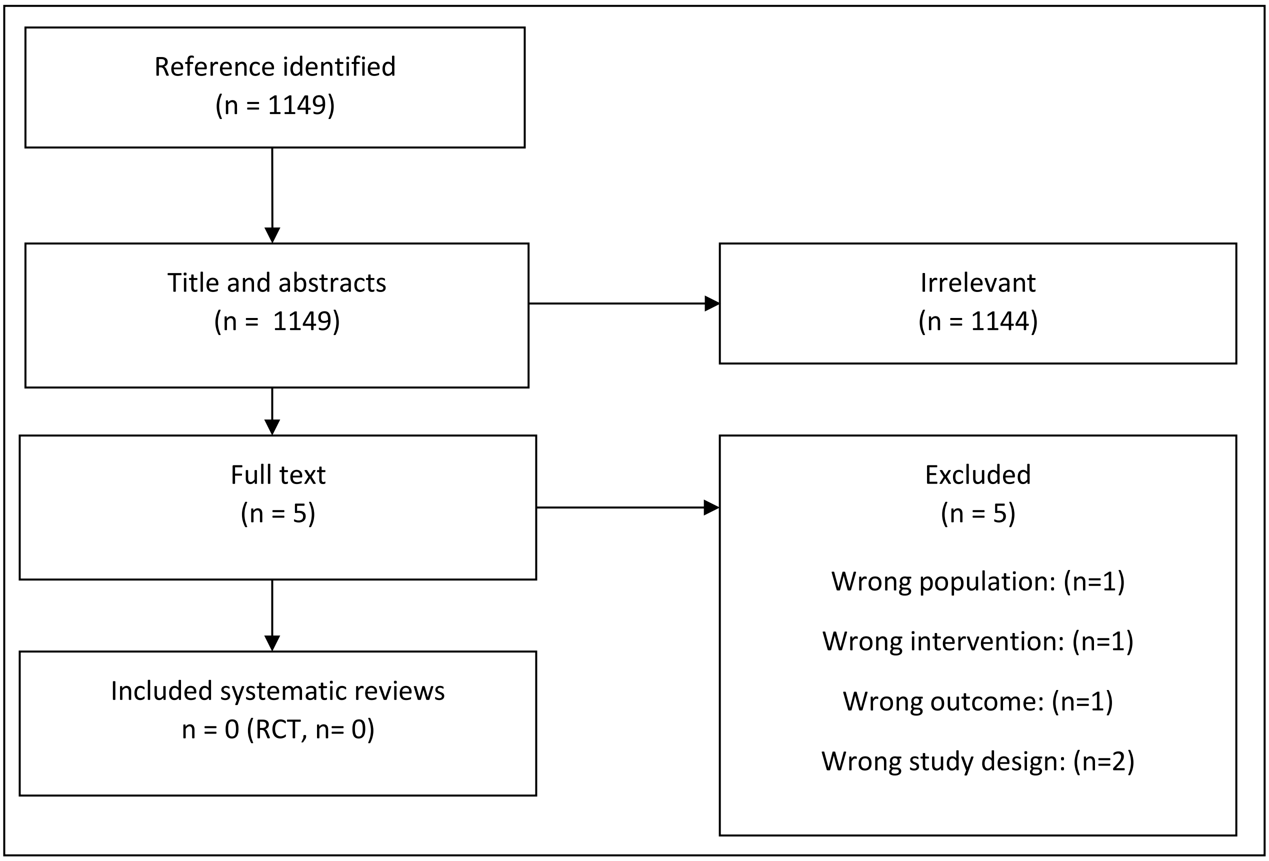

Fig. 7 Flowchart, search for systematic reviews, patient education.

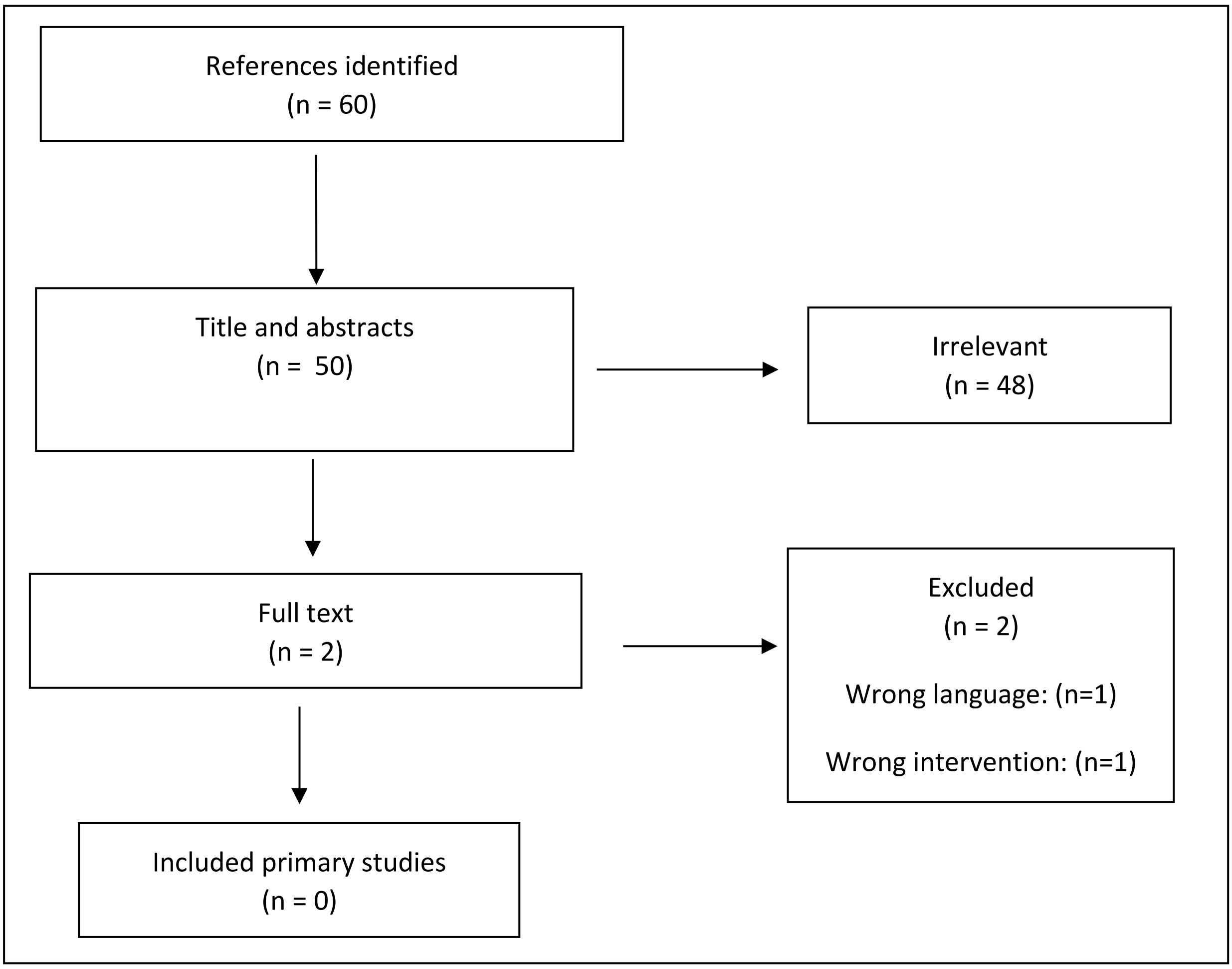

Fig. 8 Flowchart, search for primary studies, manual joint mobilisation techniques.

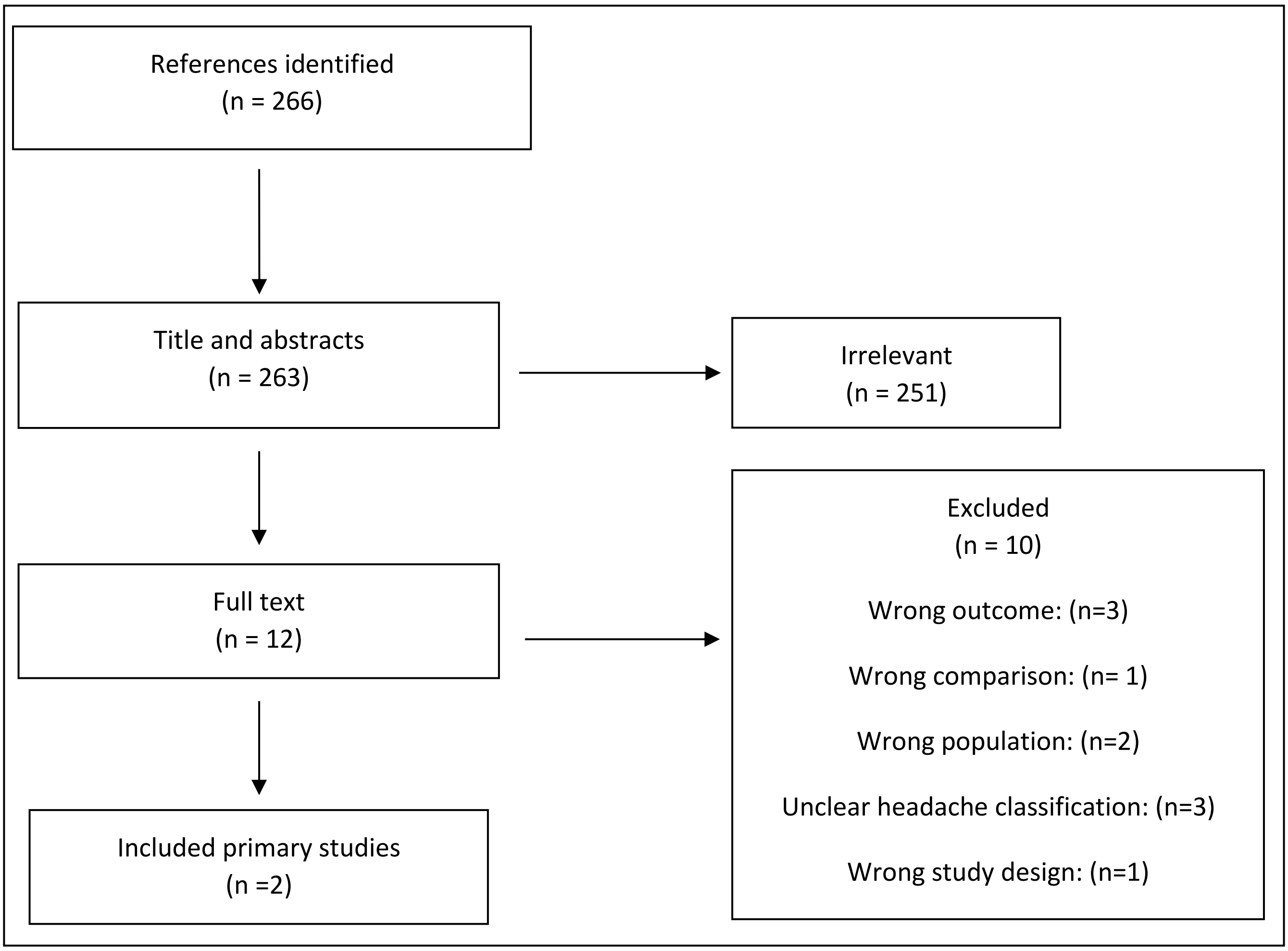

Fig. 9 Flowchart, search for primary studies, supervised physical activity.

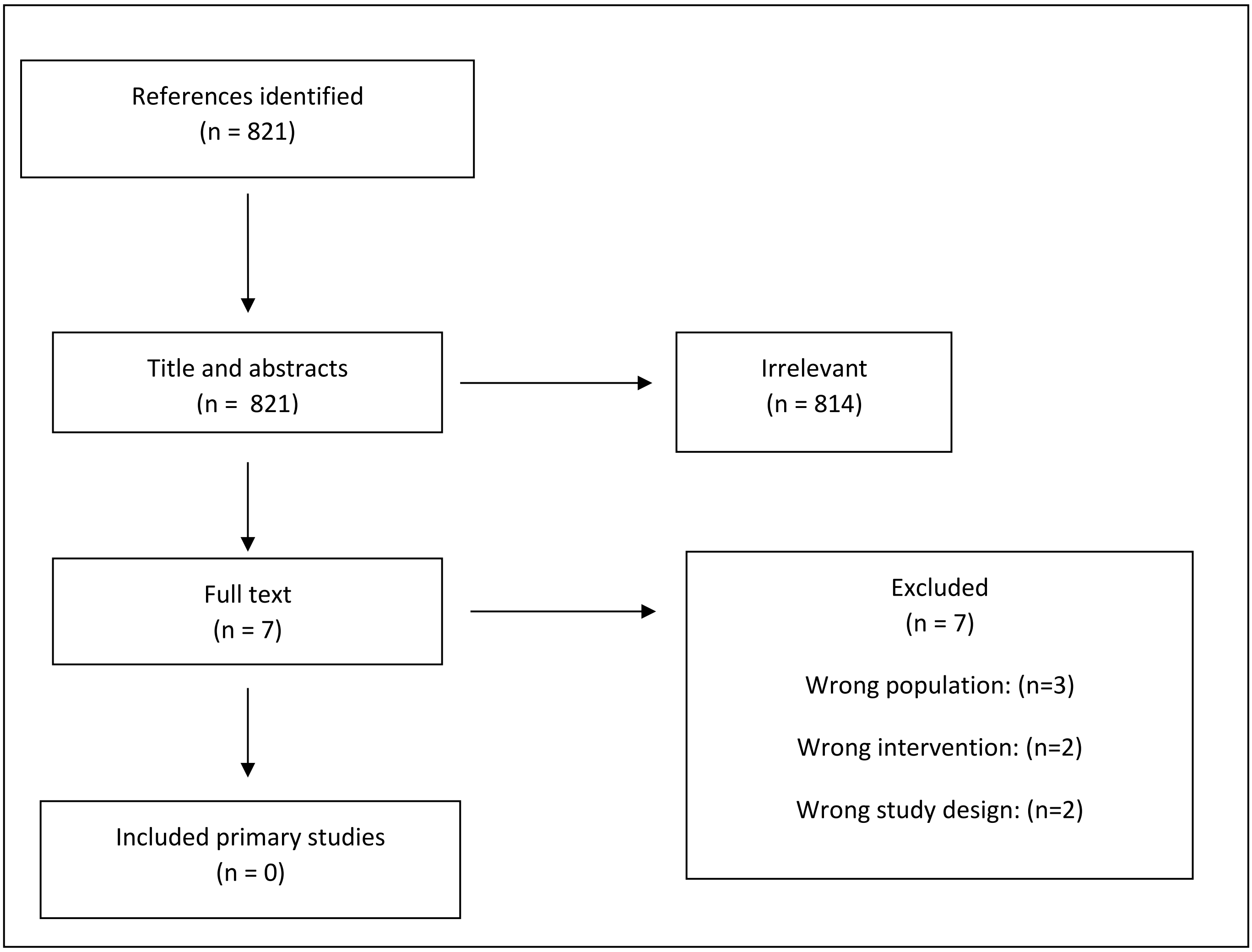

Fig. 10 Flowchart, search for primary studies, psychological treatment.

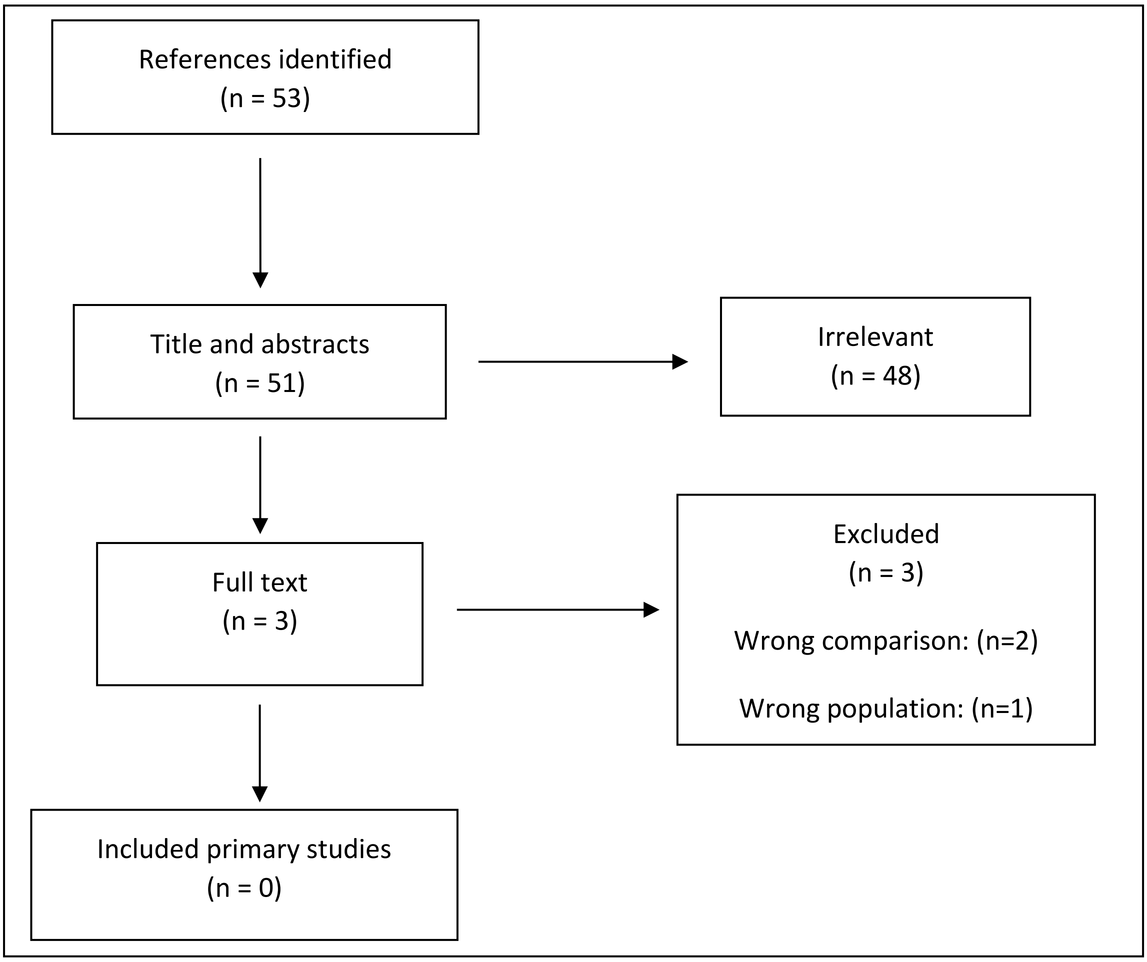

Fig. 11 Flowchart, search for primary studies, acupuncture.

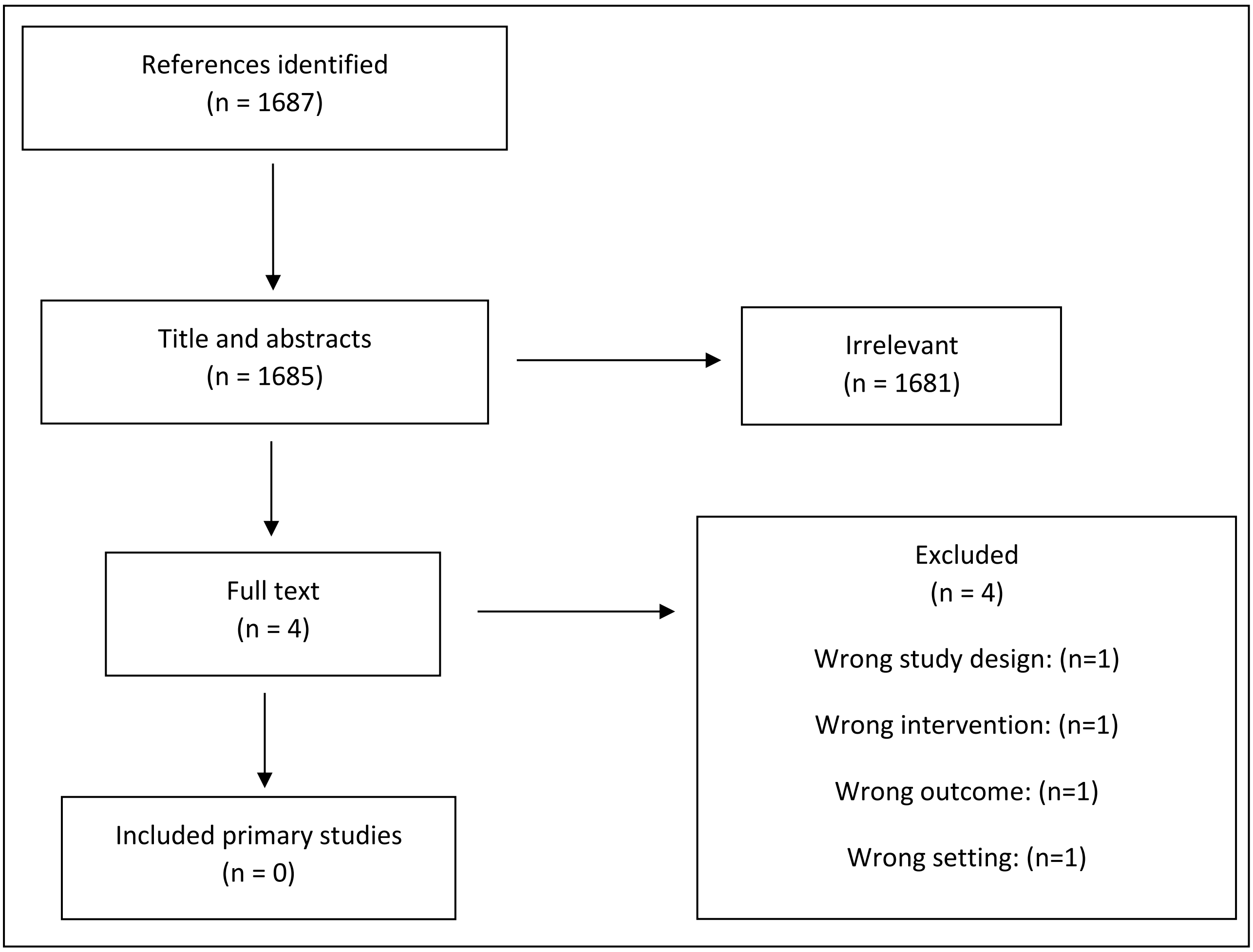

Fig. 12 Flowchart, search for primary studies, patient education.

Headache frequency, follow-up

Study or Subgroup	Intervention Mean	Intervention SD	Intervention Total	Control Mean	Control SD	Control Total	Weight	Mean Difference IV, Fixed, 95% CI	Risk of Bias A B C D E F G
Castien 2011	-9.1	4.2	40	-4.1	4.4	40	100.0%	-5.00 [-6.89, -3.11]	+ + - - + + +
**Total (95% CI)**			**40**			**40**	**100.0%**	**-5.00 [-6.89, -3.11]**	

Heterogeneity: Not applicable
Test for overall effect: Z = 5.20 (P < 0.00001)

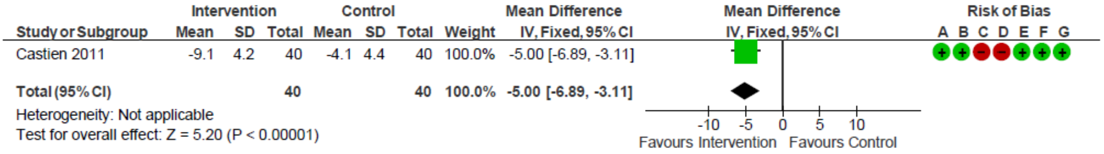

Risk of bias legend
(A) Random sequence generation (selection bias)
(B) Allocation concealment (selection bias)
(C) Blinding of participants and personnel (performance bias)
(D) Blinding of outcome assessment (detection bias)
(E) Incomplete outcome data (attrition bias)
(F) Selective reporting (reporting bias)
(G) Other bias

Quality of life, follow-up

Study or Subgroup	Intervention Mean	Intervention SD	Intervention Total	Control Mean	Control SD	Control Total	Weight	Mean Difference IV, Fixed, 95% CI	Risk of Bias A B C D E F G
Esp Lpez 2016	41.42	2.36	19	39.58	2.43	19	100.0%	1.84 [0.32, 3.36]	+ ? ? + + - +
**Total (95% CI)**			**19**			**19**	**100.0%**	**1.84 [0.32, 3.36]**	

Heterogeneity: Not applicable
Test for overall effect: Z = 2.37 (P = 0.02)

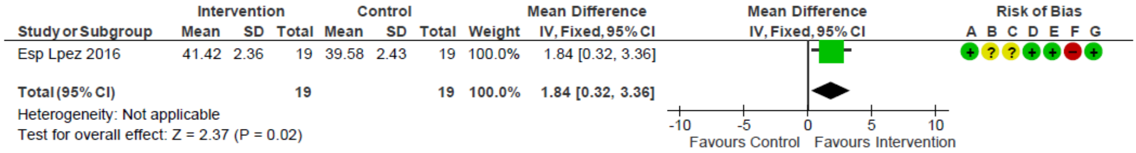

Risk of bias legend
(A) Random sequence generation (selection bias)
(B) Allocation concealment (selection bias)
(C) Blinding of participants and personnel (performance bias)
(D) Blinding of outcome assessment (detection bias)
(E) Incomplete outcome data (attrition bias)
(F) Selective reporting (reporting bias)
(G) Other bias

Headache intensity, end of treatment

Study or Subgroup	Intervention Mean	Intervention SD	Intervention Total	Control Mean	Control SD	Control Total	Weight	Std. Mean Difference IV, Random, 95% CI	Risk of Bias A B C D E F G
Castien 2011	-2.7	0.9	40	-0.9	2.4	40	52.7%	-0.98 [-1.45, -0.52]	+ + - - + + +
Esp Lpez 2014a	3.77	2.51	20	3.95	2.12	20	47.3%	-0.08 [-0.70, 0.54]	+ ? + ? + + +
**Total (95% CI)**			**60**			**60**	**100.0%**	**-0.55 [-1.44, 0.33]**	

Heterogeneity: Tau² = 0.33; Chi² = 5.27, df = 1 (P = 0.02); I² = 81%
Test for overall effect: Z = 1.22 (P = 0.22)

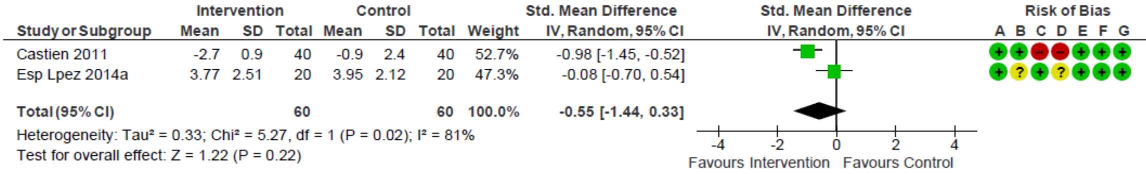

Risk of bias legend
(A) Random sequence generation (selection bias)
(B) Allocation concealment (selection bias)
(C) Blinding of participants and personnel (performance bias)
(D) Blinding of outcome assessment (detection bias)
(E) Incomplete outcome data (attrition bias)
(F) Selective reporting (reporting bias)
(G) Other bias

Functioning, end of treatment

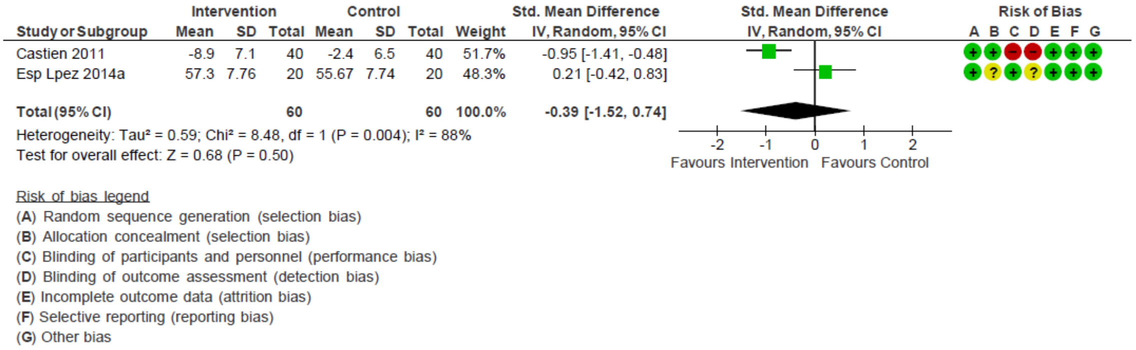

Serious adverse events, end of treatment

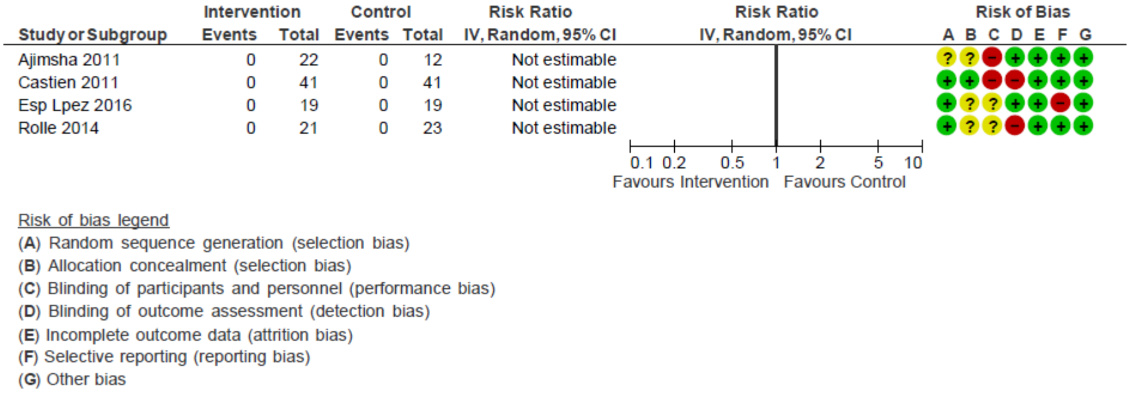

Fig. 13 Meta-analyses of manual joint mobilisation techniques, secondary outcomes.

Headache frequency, follow-up

Study or Subgroup	Intervention Mean	Intervention SD	Intervention Total	Control Mean	Control SD	Control Total	Weight	Mean Difference IV, Fixed, 95% CI	Risk of Bias A	B	C	D	E	F	G
Alvarez Melcon 2018	7.14	3.5	76	8.64	3.59	76	100.0%	-1.50 [-2.63, -0.37]	+	?	−	+	+	+	+
**Total (95% CI)**			**76**			**76**	**100.0%**	**-1.50 [-2.63, -0.37]**							

Heterogeneity: Not applicable
Test for overall effect: Z = 2.61 (P = 0.009)

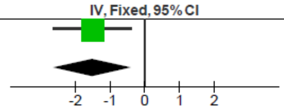

Risk of bias legend
(A) Random sequence generation (selection bias)
(B) Allocation concealment (selection bias)
(C) Blinding of participants and personnel (performance bias)
(D) Blinding of outcome assessment (detection bias)
(E) Incomplete outcome data (attrition bias)
(F) Selective reporting (reporting bias)
(G) Other bias

Headache intensity, end of treatment

Study or Subgroup	Intervention Mean	Intervention SD	Intervention Total	Control Mean	Control SD	Control Total	Weight	Mean Difference IV, Fixed, 95% CI	Risk of Bias A	B	C	D	E	F	G
Alvarez Melcon 2018	4.58	1.53	76	5.19	1.5	76	100.0%	-0.61 [-1.09, -0.13]	+	?	−	+	+	+	+
**Total (95% CI)**			**76**			**76**	**100.0%**	**-0.61 [-1.09, -0.13]**							

Heterogeneity: Not applicable
Test for overall effect: Z = 2.48 (P = 0.01)

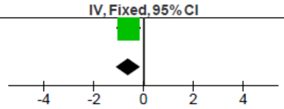

Risk of bias legend
(A) Random sequence generation (selection bias)
(B) Allocation concealment (selection bias)
(C) Blinding of participants and personnel (performance bias)
(D) Blinding of outcome assessment (detection bias)
(E) Incomplete outcome data (attrition bias)
(F) Selective reporting (reporting bias)
(G) Other bias

Acute medication intake in days, end of treatment

Study or Subgroup	Intervention Mean	Intervention SD	Intervention Total	Control Mean	Control SD	Control Total	Weight	Mean Difference IV, Fixed, 95% CI	Risk of Bias A	B	C	D	E	F	G
Alvarez Melcon 2018	5.13	3.76	76	6.13	4.06	76	100.0%	-1.00 [-2.24, 0.24]	+	?	−	+	+	+	+
**Total (95% CI)**			**76**			**76**	**100.0%**	**-1.00 [-2.24, 0.24]**							

Heterogeneity: Not applicable
Test for overall effect: Z = 1.58 (P = 0.12)

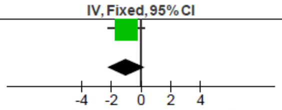

Risk of bias legend
(A) Random sequence generation (selection bias)
(B) Allocation concealment (selection bias)
(C) Blinding of participants and personnel (performance bias)
(D) Blinding of outcome assessment (detection bias)
(E) Incomplete outcome data (attrition bias)
(F) Selective reporting (reporting bias)
(G) Other bias

Fig. 14 Meta-analyses of supervised physical activity, secondary outcomes.

Stress symptoms, end of treatment

	Intervention			Control				Mean Difference	Risk of Bias						
Study or Subgroup	Mean	SD	Total	Mean	SD	Total	Weight	IV, Fixed, 95% CI	A	B	C	D	E	F	G
Omidi 2015	12.7	2.69	30	16.13	2.44	30	100.0%	-3.43 [-4.73, -2.13]	+	?	?	?	+	+	+
**Total (95% CI)**			**30**			**30**	**100.0%**	**-3.43 [-4.73, -2.13]**							

Heterogeneity: Not applicable

Test for overall effect: Z = 5.17 (P < 0.00001)

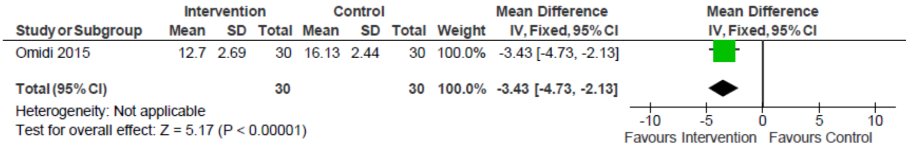

Risk of bias legend

(**A**) Random sequence generation (selection bias)
(**B**) Allocation concealment (selection bias)
(**C**) Blinding of participants and personnel (performance bias)
(**D**) Blinding of outcome assessment (detection bias)
(**E**) Incomplete outcome data (attrition bias)
(**F**) Selective reporting (reporting bias)
(**G**) Other bias

Fig. 15 Meta-analyses of psychological treatment, secondary outcomes.

Serious adverse events, end of treatment

Study or Subgroup	Intervention Events	Intervention Total	Control Events	Control Total	Weight	Risk Ratio IV, Random, 95% CI	Risk of Bias A B C D E F G
Jena 2008	0	1613	0	1569		Not estimable	+ ? ? ? ? + +
**Total (95% CI)**		**1613**		**1569**		**Not estimable**	
Total events	0		0				
Heterogeneity: Not applicable							
Test for overall effect: Not applicable							

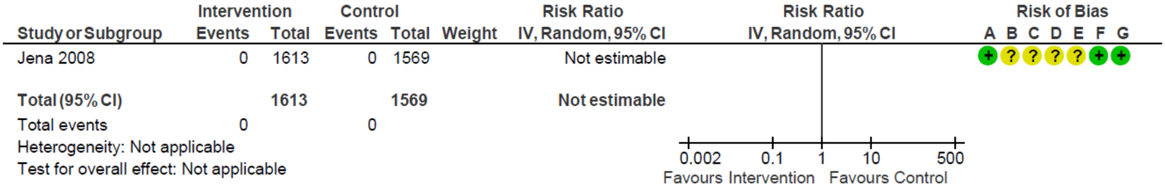

Risk of bias legend
(**A**) Random sequence generation (selection bias)
(**B**) Allocation concealment (selection bias)
(**C**) Blinding of participants and personnel (performance bias)
(**D**) Blinding of outcome assessment (detection bias)
(**E**) Incomplete outcome data (attrition bias)
(**F**) Selective reporting (reporting bias)
(**G**) Other bias

Fig. 16 Meta-analyses of acupuncture, secondary outcomes.

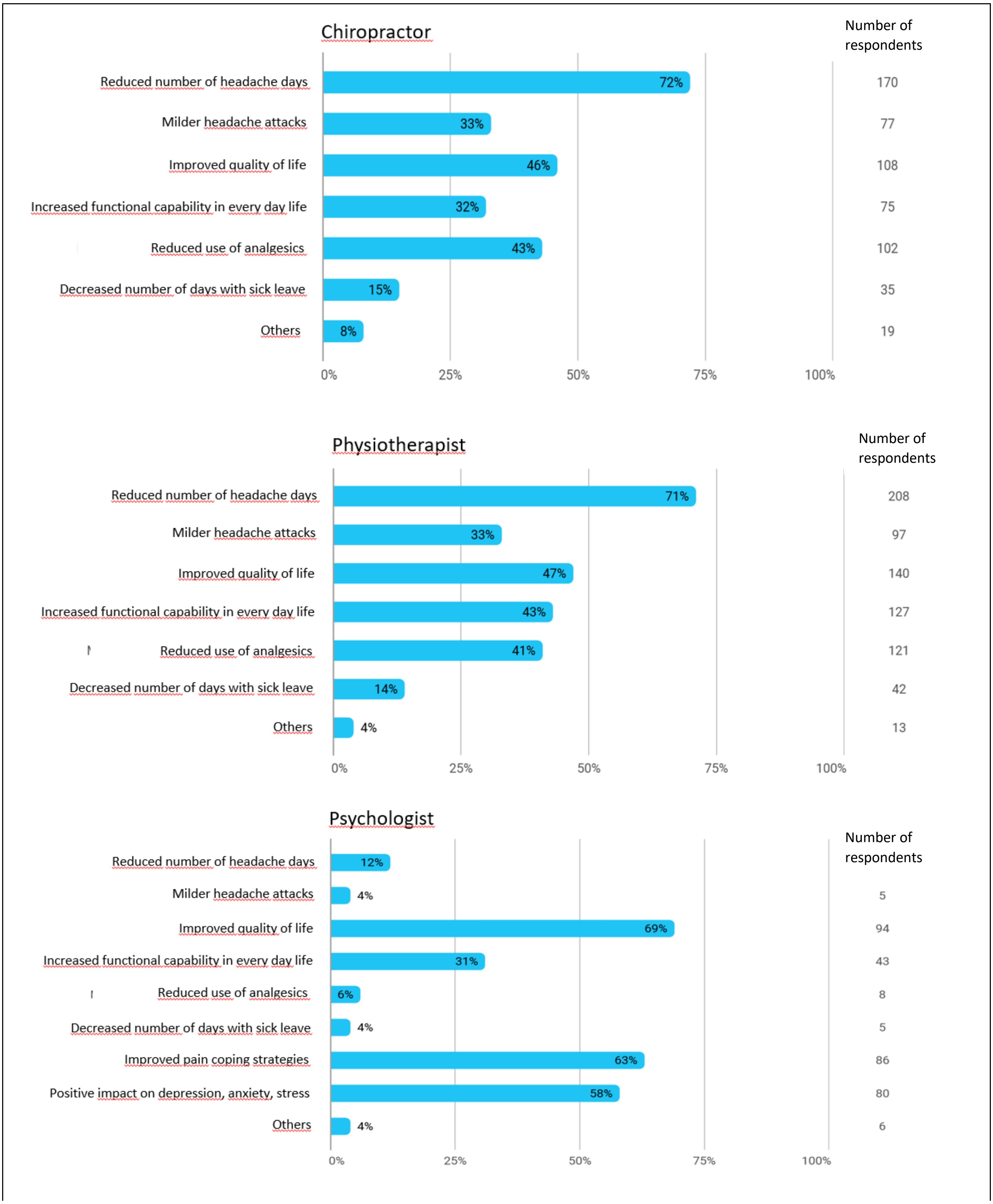

Chiropractor
Number of respondents
Reduced number of headache days 72% 170
Milder headache attacks 33% 77
Improved quality of life 46% 108
Increased functional capability in every day life 32% 75
Reduced use of analgesics 43% 102
Decreased number of days with sick leave 15% 35
Others 8% 19
0% 25% 50% 75% 100%
Physiotherapist
Number of respondents
Reduced number of headache days 71% 208
Milder headache attacks 33% 97
Improved quality of life 47% 140
Increased functional capability in every day life 43% 127
Reduced use of analgesics 41% 121
Decreased number of days with sick leave 14% 42
Others 4% 13
0% 25% 50% 75% 100%
Psychologist
Number of respondents
Reduced number of headache days 12%
Milder headache attacks 4% 5
Improved quality of life 69% 94
Increased functional capability in every day life 31% 43
Reduced use of analgesics 6% 8
Decreased number of days with sick leave 4% 5
Improved pain coping strategies 63% 86
Positive impact on depression, anxiety, stress 58% 80
Others 4% 6
0% 25% 50% 75% 100%

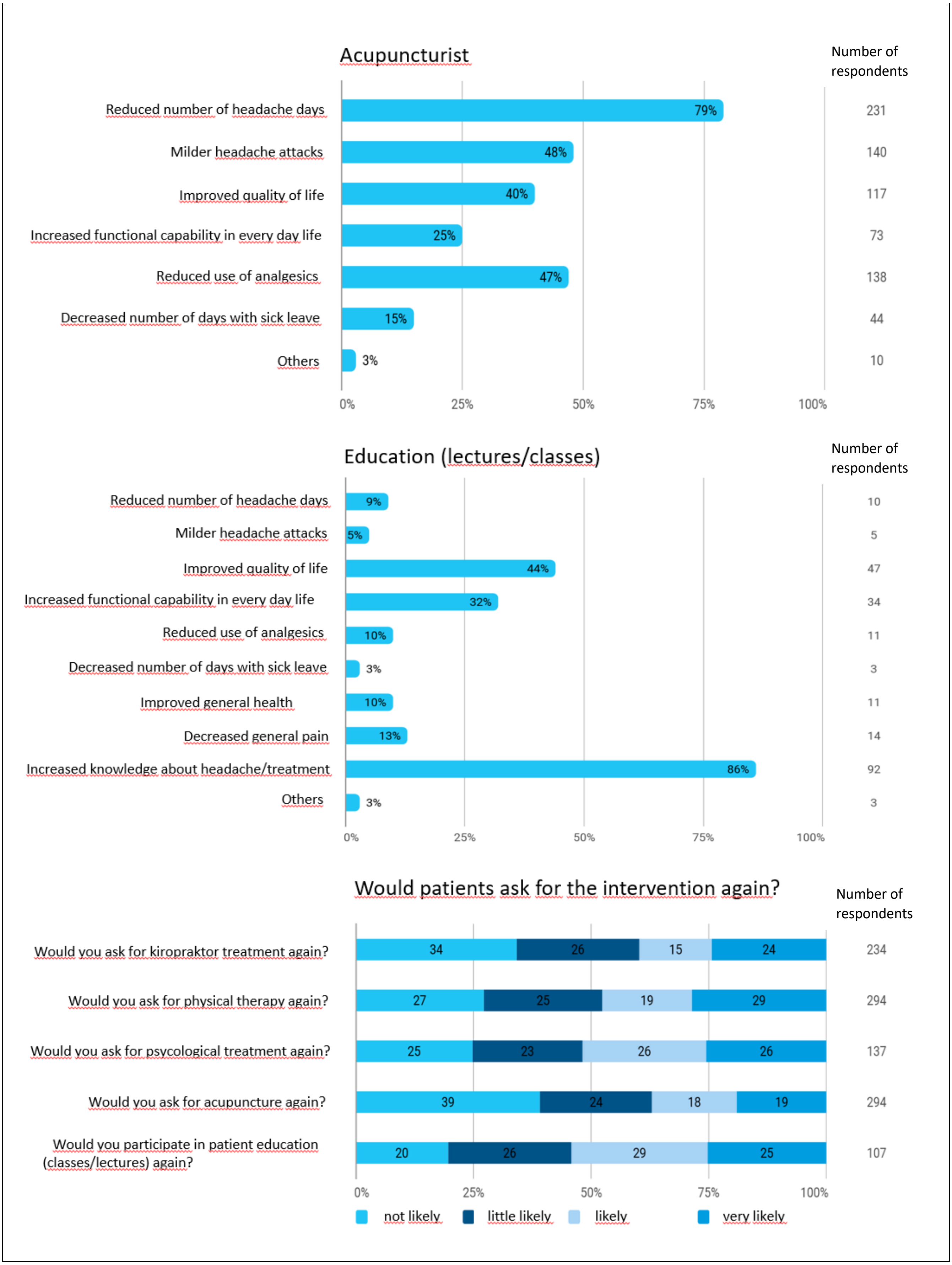

Fig. 17 Survey of patients' expectations for treatment effects in chiropractor, physiotherapist, psychologist, acupuncturist, and education (lectures/classes), and whether patients would ask for these interventions again.
