# Supplementary material for: Manual joint mobilisation techniques, supervised physical activity, psychological treatment, acupuncture and patient education for patients with tension-type headache. A systematic review and meta-analysis
Source: J Headache Pain. 2021 Aug 21;22(1):96. doi: 10.1186/s10194-021-01298-4 (PMC8379845; doi:10.1186/s10194-021-01298-4)
Supplement: Supplementary file 2 — Additional file 2. Search protocol. [file 10194_2021_1298_MOESM2_ESM.pdf]

SEARCH PROTOCOL

Project title	Non-pharmacological treatment of tension-type headache
Project manager	Christel Høst, Nationalt Videnscenter for Hovedpine
Lead	Louise Ninett Carlsen, Nationalt Videnscenter for Hovedpine
Search specialist	Kirsten Birkefoss, Danish Health Authority, StepChange
Last updated	14.07.2020

SEARCH STRATEGY

**Tension-type headache**	Should patients with tension-type headache be offered treatment with manual joint-mobilizing techniques besides usual care?<br><br>Should patients with tension-type headache be offered physical activity?<br><br>Should patients with tension-type headache be offered psychological treatment?<br><br>Should patients with tension-type headache be offered acupuncture?<br><br>Should patients with tension-type headache be offered patient education besides usual treatment?
**Search terms**	See search terms in the search protocol below
**Inclusion and exclusion criterias**	Language: English, Danish, Norwegian, Swedish<br>Pub Year: 2010-2020<br>Population: Adults 18 and older<br>Publ.types: Guidelines, systematic reviews, meta analyses, randomized controlled trials or controlled trals

Information sources

DATABASE	INTERFACE
**Medline (incl. Cochrane Reviews)**	OVID
**EMBASE (incl. Cochrane Reviews)**	OVID
**PsycINFO**	OVID
**CINAHL**	EBSCO
**PEDRO**	Internet
**OT Seeker**	Internet

Note

- Search terms and inclusion/exclusion criterias are adapted to the different database languages.
- Duplicates have as thoroughly as possible been removed by the help of RefWorks.
- References found has been transferred to Covidence

Search for systematic reviews and meta analyses

Should patients with tension-type headache be offered treatment with manual joint-mobilizing techniques besides usual care?

Search date: 19.05.2020

Medline

Database(s): **Ovid MEDLINE(R) and Epub Ahead of Print, In-Process & Other Non-Indexed Citations, Daily and Versions(R)** 1946 to May 18, 2020
Search Strategy:

#	Searches
1	Headache/
2	Headache disorders/
3	Headache Disorders, Primary/
4	exp Migraine Disorders/
5	exp Tension-Type Headache/
6	(headache* or head ache* or head pain or cephalalgia* or cephalgia* or migraine* or Cephalodynia* or cranial pain or hemicranias* or cerebral pain or cranialgia*).ti,bt,ab,kw,kf.
7	or/1-6
8	exp Musculoskeletal Manipulations/
9	Manipulation, Chiropractic/
10	Manipulation, Orthopedic/
11	Manipulation, Osteopathic/
12	Manipulation, Spinal/
13	Osteopathic Medicine/
14	exp Physical therapy modalities/
15	(adjust* adj6 (chiropract* or spinal or spine or lumbar or cervical or neck or thoracic or instrument* or tool* or electric)).ti,bt,ab,kf.
16	(HVLA or high velocity low amplitude).ti,bt,ab,kw.
17	(manipulat* and (chiropract* or naprapath* or osteopath* or oesteopath* or orthopedic* or orthopaedic*)).ti,bt,ab,kf.
18	(manipulat* and (spinal or spine or low* back or joint* or lumbar or neck or thoracic or cervical or cervix or MSK or musculoskeletal or musculo-skeletal or vertebr*)).ti,bt,ab,kf.

19	(manipulat* and (physiotherap* or physical therap*)).ti,bt,ab,kf.
20	(manipulat* and technique*).ti,bt,ab,kw,kf.
21	(mobili#at* and (chiropract* or naprapath* or osteopath* or oesteopath* or orthopedic* or orthopaedic* or physical therap* or physiotherap*)).ti,bt,ab,kf.
22	(mobili#at* and (spinal or spine or low* back or joint* or lumbar or neck or thoracic or cervical or cervix or MSK or musculoskeletal or musculo-skeletal or vertebr*)).ti,bt,ab,kf.
23	((manual or manipulat* or mobili#at* or MSK or movement or musculoskeletal or musculo-skeletal or neurorehabilitati* or neuro-rehabilitati* or physical) adj6 (therap* or treat* or intervention* or action* or program* or strateg* or protocol* or support* or approach* or evaluation)).ti,bt,ab,kf.
24	OMT.ti,bt,ab,kw,kf.
25	((trigger point or motion or passive or cpm) adj2 therap*).ti,bt,ab,kw,kf.
26	(traction adj3 (manual or passive or mechanical or non-surgical or nonsurgical)).ti,bt,ab,kf.
27	(flexion-distraction or flexion distraction).ti,bt,ab,kf.
28	(activator adj3 (method* or technique* or instrument*)).ti,bt,ab,kf.
29	((mechanical or Alexander or Atlas Orthogonal or Atlas Specific or Bilateral Nasal Specific or Blair Upper Cervical or Chiropractic Manipulative Reflex or Cox or Derifield-Thompson or Directional Non-Force or Diversified or Endo-Nasal or feldenkrais or William or Flexion-Distraction or Full Spine or Gonstead or Grostic or Harrison or Koren Specific or Logan Basic or McTimoney or Maitland or Mulligan or muligan or Neuro Emotional or NUCCA or Palmer or Pierce-Stillwagon or Sacro Occipital or Thompson Terminal Point or Toftness or Toggle Recoil or Torque Release or Webster or muscle energy) adj1 (Technique* or model* or tool* or exercise* or therapy)).ti,bt,ab,kw,kf.
30	(reflexolog* or cyriax or High velocity or low amplitude or Subluxation or Joint dysfunction or hypomobility or muscle energy technique* or therapeutic touch or stretching or acupressure).ti,bt,ab,kw,kf.
31	(vibration adj5 (therap* or treatment*)).ti,bt,ab,kw,kf.
32	((non-pharma* or nonpharma* or non pharma* or non-drug or nondrug or non drug or non-surgical or nonsurgical or non surgical or non-surgery or nonsurgery or non surgery or non-invasive or noninvasive or non invasive or complimentary or integrat* or holistic or multi-modal* or multimodal* or Multidisciplinary or Multi-disciplinary or Interdisciplinary or Inter-disciplinary or complex or multimodal* or multi-modal* or cross-disciplinary or crossdisciplinary or multi-dimensional or multidimensional or biopsychosocial or bio-psycho-social or multi-facet* or multifacet* or comprehensive or multiple or addition* or physiotherapy or physical therap*) adj6 (therap* or treat* or intervention* or action* or program* or strateg* or protocol* or support* or approach* or evaluation or rehabilitation or care or session*)).ti,bt,kw,kf.
33	or/8-32
34	7 and 33

35	limit 34 to (guideline or practice guideline or systematic reviews or meta analysis)
36	(((systematic or rapid or integrative or umbrella) adj3 (review* or overview* or study or studies or search* or approach* or analys*)) or guideline* or recommendations or meta analy* or meta-analy* or metaanaly*).ti,bt,ab,kw,kf.
37	((pool* or combin*) adj1 (data or analys*)).ti,ab.
38	(pubmed or medline or embase or cochrane or "web of science" or psycinfo or psychinfo or scopus).ti,ab.
39	cochrane.jw.
40	Network meta-analysis/
41	(((multiple or mixed) adj1 (treatment* or therap*) adj1 comparison*) or (indirect adj1 comparison*)).ti,bt,ab,kw,kf.
42	or/36-41
43	34 and 42
44	35 or 43

Embase

Database(s): **Embase** 1974 to 2020 May 18
Search Strategy:

#	Searches
1	Headache/
2	"headache and facial pain"/
3	exp migraine/
4	exp tension headache/
5	exp chronic daily headache/
6	primary headache/
7	stabbing headache/
8	(headache* or head ache* or head pain or cephalalgia* or cephalgia* or migraine* or Cephalodynia* or cranial pain or hemicranias* or cerebral pain or cranialgia* or trigeminal-autonomic-cephalalgia*).ti,ab,kw.
9	or/1-8
10	exp musculoskeletal manipulation/
11	exp physiotherapy/
12	(adjust* adj6 (chiropract* or spinal or spine or lumbar or cervical or neck or thoracic or instrument* or tool* or electric)).ti,ab,kw.

13	(HVLA or high velocity low amplitude).ti,ab,kw.
14	(manipulat* and (chiropract* or naprapath* or osteopath* or oesteopath* or orthopedic* or orthopaedic*)).ti,ab,kw.
15	(manipulat* and (spinal or spine or low* back or joint* or lumbar or neck or thoracic or cervical or cervix or MSK or musculoskeletal or musculo-skeletal or vertebr*)).ti,ab,kw.
16	(manipulat* and (physiotherap* or physical therap*)).ti,ab,kw.
17	(manipulat* and technique*).ti,ab,kw.
18	(mobili?at* and (chiropract* or naprapath* or osteopath* or orthopedic* or orthopaedic* or physical therap* or physiotherap*)).ti,ab,kw.
19	(mobili?at* and (spinal or spine or low* back or joint* or lumbar or neck or thoracic or cervical or MSK or musculoskeletal or vertebr*)).ti,ab,kw.
20	((manual or manipulat* or mobili?at* or MSK or musculoskeletal or musculo-skeletal or neurorehabilitati* or neuro-rehabilitati* or physical) adj6 (therap* or treat* or intervention* or action* or program* or strateg* or protocol* or support* or approach* or evaluation)).ti,ab,kw.
21	(traction adj3 (manual or passive or mechanical or non-surgical or nonsurgical)).ti,ab,kw.
22	(flexion-distraction or flexion distraction).ti,ab,kw.
23	((activator or physiotherapy or physical therapy) adj3 (method* or technique* or instrument*)).ti,ab,kw.
24	((mechanical or Alexander or Atlas Orthogonal or Atlas Specific or Bilateral Nasal Specific or Blair Upper Cervical or Chiropractic Manipulative Reflex or Cox or Derifield-Thompson or Directional Non-Force or Diversified or Endo-Nasal or feldenkrais or William or Flexion-Distraction or Full Spine or Gonstead or Grostic or Harrison or Koren Specific or Logan Basic or McTimoney or Maitland or Mulligan or muligan or Neuro Emotional or NUCCA or Palmer or Pierce-Stillwagon or Sacro Occipital or Thompson Terminal Point or Toftness or Toggle Recoil or Torque Release or Webster or muscle energy) adj1 (Technique* or model* or tool* or exercise* or therapy)).ti,ab,kw.
25	(reflexolog* or cyriax or High velocity or low amplitude or Subluxation or Joint dysfunction or hypomobility or muscle energy technique* or therapeutic touch or stretching or acupressure).ti,ab,kw.
26	OMT.ti,ab,kw.
27	(vibration adj5 (therap* or treatment*)).ti,ab,kw.
28	((non-pharma* or nonpharma* or non pharma* or non-drug or nondrug or non drug or non-surgical or nonsurgical or non surgical or non-surgery or nonsurgery or non surgery or non-invasive or noninvasive or non invasive or complimentary or integrat* or holistic or multi-modal* or multimodal* or Multidisciplinary or Multi-disciplinary or Interdisciplinary or Inter-disciplinary or complex or multimodal* or multi-modal* or cross-disciplinary or crossdisciplinary or multi-dimensional or multidimensional or biopsychosocial or bio-psycho-social or multi-facet* or multifacet* or comprehensive or multiple or addition* or physiotherapy or physical therap*) adj6 (therap* or treat*

	or intervention* or action* or program* or strateg* or protocol* or support* or approach* or evaluation or rehabilitation or care or session*)).ti,kw.
29	or/10-28
30	9 and 29
31	limit 30 to ("systematic review" or meta analysis)
32	(((systematic or rapid or integrative or umbrella) adj3 (review* or overview* or study or studies or search* or approach* or analys*)) or guideline* or recommendations or meta analy* or meta-analy* or metaanaly*).ti,ab,kw.
33	((pool* or combin*) adj1 (data or analys*)).ti,ab.
34	(pubmed or medline or embase or cochrane or "web of science" or psycinfo or psychinfo or scopus).ti,ab.
35	cochrane.jx.
36	Network meta-analysis/
37	(((multiple or mixed) adj1 (treatment* or therap*) adj1 comparison*) or (indirect adj1 comparison*)).ti,ab,kw.
38	or/32-37
39	30 and 38
40	31 or 39

Cinahl

#	Query	Limiters/Expanders
S22	S16 AND S21	Language: Danish, English, Norwegian, Swedish
S21	S17 OR S18 OR S19 OR S20	
S20	(((multiple or mixed) adj1 (treatment* or therap*) N1 comparison*) or (indirect N1 comparison*))	
S19	(pool* N1 (data or analys*))	
S18	(((systematic or rapid or integrative or umbrella) N3 (review* or overview* or study or studies or search* or approach* or analys*)) or guideline* or recommendations or meta analy* or meta-analy* or metaanaly*)	
S17	PT (Systematic Review or Meta Analysis) or MH ("Systematic Review+" or "Meta Analysis+")	
S16	S4 AND S15	

S15	S5 OR S6 OR S7 OR S8 OR S9 OR S10 OR S11 OR S12 OR S13 OR S14	
S14	((non-pharma* or nonpharma* or non pharma* or non-drug or nondrug or non drug or non-surgical or nonsurgical or non surgical or non-surgery or nonsurgery or non surgery or non-invasive or noninvasive or non invasive or complimentary or integrat* or holistic or multi-modal* or multimodal* or Multidisciplinary or Multi-disciplinary or Interdisciplinary or Inter-disciplinary or complex or multimodal* or multi-modal* or cross-disciplinary or crossdisciplinary or multi-dimensional or multidimensional or biopsychosocial or bio-psycho-social or multi-facet* or multifacet* or comprehensive or multiple or physiotherapy or physical therap*) N6 (therap* or treat* or intervention* or action* or program* or strateg* or protocol* or support* or approach* or evaluation or rehabilitation or care or session*))	
S13	(vibration N5 (therap* or treatment*))	
S12	reflexolog* or cyriax or High velocity or low amplitude or Subluxation or Joint dysfunction or hypomobility or muscle energy technique* or therapeutic touch or stretching or acupressure	
S11	((mechanical or Alexander or Atlas Orthogonal or Atlas Specific or Bilateral Nasal Specific or Blair Upper Cervical or Chiropractic Manipulative Reflex or Cox or Derifield-Thompson or Directional Non-Force or Diversified or Endo-Nasal or feldenkrais or William or Flexion-Distraction or Full Spine or Gonstead or Grostic or Harrison or Koren Specific or Logan Basic or McTimoney or Maitland or Mulligan or muligan or Neuro Emotional or NUCCA or Palmer or Pierce-Stillwagon or Sacro Occipital or Thompson Terminal Point or Toftness or Toggle Recoil or Torque Release or Webster or muscle energy or physical therapy or physiotherapy) N1 (Technique* or model* or tool* or exercise* or therapy))	
S10	((manual or manipulat* or mobili?at* or MSK or musculoskeletal or musculo-skeletal or neurorehabilitati* or neuro-rehabilitati* or physical) N6 (therap* or treat* or intervention* or action* or program* or strateg* or protocol* or support* or approach* or evaluation or session*)) or ((traction N3 (manual or passive or mechanical or non-surgical or nonsurgical))) or (activator N3 (method* or technique* or instrument*))	
S9	(mobili?at* and (chiropract* or naprapath* or osteopath* or oesteopath* or orthopedic* or orthopaedic* or spinal or spine or low* back or joint* or lumbar or neck or thoracic or cervical or cervix or MSK or musculoskeletal or musculo-skeletal or vertebr*))	

S8	(manipulat* and (chiropract* or naprapath* or osteopath* or oesteopath* or orthopedic* or orthopaedic* or spinal or spine or low* back or joint* or lumbar or neck or thoracic or cervical or cervix or MSK or musculoskeletal or musculo-skeletal or vertebr* or physiotherap* or physical therap* or technique*))	
S7	HVLA or high velocity low amplitude or flexion-distraction or flexion distraction	
S6	(adjust* N6 (chiropract* or spinal or spine or lumbar or cervical or neck or thoracic or instrument* or tool* or electric))	
S5	MH "Physical therapy" or MH "Manual therapy+" or MH "Joint mobilization"	
S4	S1 OR S2 OR S3	
S3	SU (headache* or head ache* or head pain or cephalalgia* or cephalgia* or migraine* or Cephalodynia* or cranial pain or hemicranias* or cerebral pain or cranialgia*)	
S2	(headache* or head ache* or head pain or cephalalgia* or cephalgia* or migraine* or Cephalodynia* or cranial pain or hemicranias* or cerebral pain or cranialgia*)	
S1	MH "Headache" or MH "Headache, Primary" or MH "Migraine" or MH "Tension headache"	

PEDRO

Abstract & Title: headache or migraine*

Method: Practice guideline or Systematic review

Should patients with tension-type headache be offered physical activity?

Search date: 19.05.2020

Medline

Database(s): **Ovid MEDLINE(R) and Epub Ahead of Print, In-Process & Other Non-Indexed Citations, Daily and Versions(R)** 1946 to May 18, 2020
Search Strategy:

#	Searches
1	Headache/
2	Headache disorders/
3	Headache Disorders, Primary/
4	exp Migraine Disorders/
5	exp Tension-Type Headache/
6	(headache* or head ache* or head pain or cephalalgia* or cephalgia* or migraine* or Cephalodynia* or cranial pain or hemicranias* or cerebral pain or cranialgia*).ti,bt,ab,kw,kf.
7	or/1-6
8	exp exercise/
9	exp exercise therapy/
10	exp Sports/
11	exp Physical Exertion/
12	exp Muscle strength/
13	exp Exercise Movement Techniques/
14	exp "Physical Education and Training"/
15	Weight-Bearing/
16	Tai Ji/
17	Yoga/
18	Qigong/
19	physical therapy modalities/
20	(exercis* or train* or stepped care or physical therap* or physiotherap* or kinesiotherapy).ti,bt,kw,kf.
21	(physical adj1 (education or program*)).ti,bt,ab,kw,kf.
22	(exercise adj3 (session* or training or technique* or physical or isometric or therap* or program* or class*)).ti,bt,ab,kw,kf.

23	((muscl* or resistance or physical or endurance or strength* or balance or postur* or gait or threshold* or treadmill* or aquatic* or trunk or graded or gradual or progressive or controlled or incremented or symptom-based or symptombased or symptom based or guided or supervis* or coached or coaching or stepped care or stepwise or step-wise) adj3 (train* or retrain* or re-train* or exercise* or rehabilitation or strength* or fit* or condition* or exposure or activit* or exertion or load* or physical therap* or physiotherap*)).ti,bt,ab,kw,kf.
24	(active rehabilitation or aerobic* or anaerobic* or movement therapy or watergym* or water gym* or aquagym or gym* or hydrotherap* or (warm water adj4 (exercis* or train* or rehab*)) or pilates).ti,bt,ab,kw,kf.
25	(running or Skate* or skating or skiing or Jog* or zumba or kettlebelt or Swim* or Bicycl* or cycling or bicycling or fitness bike* or exercise bike* or spinning or walk* or rowing or rower or water-rower* or waterrower* or cross-train* or crosstrain* or pacing or paced or titreted or (weight* adj3 (lift* or train* or exercis*))).ti,bt,ab,kw,kf.
26	(yoga or tai ji or tai chi or qigong or qi-gong or pilates*).ti,bt,ab,kw,kf.
27	((mckenzie or mechanical or Alexander or William or Feldenkrais) adj2 (technique* or model* or tool* or exercise* or therapy)).ti,bt,ab,kw,kf.
28	(neurorehabilitati* or neuro-rehabilitati*).ti,bt,ab,kw,kf.
29	((non-pharma* or nonpharma* or non pharma* or non-drug or nondrug or non drug or non-surgical or nonsurgical or non surgical or non-surgery or nonsurgery or non surgery or non-invasive or noninvasive or non invasive or complimentary or integrat* or holistic or multi-modal* or multimodal* or Multidisciplinary or Multi-disciplinary or Interdisciplinary or Inter-disciplinary or complex or multimodal* or multi-modal* or cross-disciplinary or crossdisciplinary or multi-dimensional or multidimensional or biopsychosocial or bio-psycho-social or multi-facet* or multifacet* or comprehensive or multiple or addition* or physiotherapy or physical therap*) adj6 (therap* or treat* or intervention* or action* or program* or strateg* or protocol* or support* or approach* or evaluation or rehabilitation or care)).ti,bt,kw,kf.
30	or/8-29
31	7 and 30
32	limit 31 to (guideline or practice guideline or systematic reviews or meta analysis)
33	(((systematic or rapid or integrative or umbrella) adj3 (review* or overview* or study or studies or search* or approach* or analys*)) or guideline* or recommendations or meta analy* or meta-analy* or metaanaly*).ti,bt,ab,kw,kf.
34	(pool* adj1 (data or analys*)).ti,ab.
35	(pubmed or medline or embase or cochrane or "web of science" or psycinfo or psychinfo or scopus).ti,ab.
36	cochrane.jw.
37	Network meta-analysis/

38	(((multiple or mixed) adj1 (treatment* or therap*) adj1 comparison*) or (indirect adj1 comparison*)).ti,bt,ab,kw,kf.
39	or/33-38
40	31 and 39
41	32 or 40

Embase

Database(s): **Embase** 1974 to 2020 May 18
Search Strategy:

#	Searches
1	Headache/
2	"headache and facial pain"/
3	exp migraine/
4	exp tension headache/
5	exp chronic daily headache/
6	primary headache/
7	stabbing headache/
8	(headache* or head ache* or head pain or cephalalgia* or cephalgia* or migraine* or Cephalodynia* or cranial pain or hemicranias* or cerebral pain or cranialgia*).ti,ab,kw.
9	or/1-8
10	exp exercise/
11	exp kinesiotherapy/
12	exp Physical Exertion/
13	exp Muscle strength/
14	exp Exercise Movement Techniques/
15	exp "Physical Education and Training"/
16	Weight-Bearing/
17	Tai Ji/
18	Yoga/
19	Qigong/
20	physiotherapy/
21	(exercis* or train* or stepped care or physical therap* or physiotherap* or kinesiotherapy).ti,kw.

22	(physical adj1 (education or program*)).ti,ab,kw.
23	(exercise adj3 (session* or training or technique* or physical or isometric or therap* or program* or class*)).ti,ab,kw.
24	((muscl* or resistance or physical or endurance or strength* or balance or postur* or gait or threshold* or treadmill* or aquatic* or trunk or graded or gradual or progressive or controlled or incremented or symptom-based or symptombased or symptom based or guided or supervis* or coached or coaching or stepped care or stepwise or step-wise) adj3 (train* or retrain* or re-train* or exercise* or rehabilitation or strength* or fit* or condition* or exposure or activit* or exertion or load* or physical therap* or physiotherap*)).ti,ab,kw.
25	(active rehabilitation or aerobic* or anaerobic* or movement therapy or watergym* or water gym* or aquagym or gym* or hydrotherap* or (warm water adj4 (exercis* or train* or rehab))).ti,ab,kw.
26	(running or Skate* or skating or skiing or Jog* or zumba or kettlebelt or Swim* or Bicycl* or cycling or bicycling or fitness bike* or exercise bike* or spinning or walk* or rowing or rower or water-rower* or waterrower* or cross-train* or crosstrain* or pacing or paced or titreted or (weight* adj3 (lift* or train* or exercis*))).ti,ab,kw.
27	(yoga or tai ji or tai chi or qigong or qi-gong or pilates).ti,ab,kw.
28	((mckenzie or mechanical or Alexander or William or Feldenkrais) adj2 (technique* or model* or tool* or exercise* or therapy)).ti,ab,kw.
29	(neurorehabilitati* or neuro-rehabilitati*).ti,ab,kw.
30	((non-pharma* or nonpharma* or non pharma* or non-drug or nondrug or non drug or non-surgical or nonsurgical or non surgical or non-surgery or nonsurgery or non surgery or non-invasive or noninvasive or non invasive or complimentary or integrat* or holistic or multi-modal* or multimodal* or Multidisciplinary or Multi-disciplinary or Interdisciplinary or Inter-disciplinary or complex or multimodal* or multi-modal* or cross-disciplinary or crossdisciplinary or multi-dimensional or multidimensional or biopsychosocial or bio-psycho-social or multi-facet* or multifacet* or comprehensive or multiple or addition* or physiotherapy or physical therap*) adj3 (therap* or treat* or intervention* or action* or program* or strateg* or protocol* or support* or approach* or evaluation or rehabilitation or care or session*)).ti,kw.
31	or/10-30
32	9 and 31
33	limit 32 to ("systematic review" or meta analysis)
34	(((systematic or rapid or integrative or umbrella) adj3 (review* or overview* or study or studies or search* or approach* or analys*)) or guideline* or recommendations or meta analy* or meta-analy* or metaanaly*).ti,ab,kw.
35	((pool* or combin*) adj1 (data or analys*)).ti,ab.
36	(pubmed or medline or embase or cochrane or "web of science" or psycinfo or psychinfo or scopus).ti,ab.

37	cochrane.jx.
38	Network meta-analysis/
39	(((multiple or mixed) adj1 (treatment* or therap*) adj1 comparison*) or (indirect adj1 comparison*)).ti,ab,kw.
40	or/34-39
41	32 and 40
42	33 or 41

Cinahl

#	Query	Limiters/Expanders
S22	S16 AND S21	Language: Danish, English, Norwegian, Swedish
S21	S17 OR S18 OR S19 OR S20	
S20	(((multiple or mixed) adj1 (treatment* or therap*) N1 comparison*) or (indirect N1 comparison*))	
S19	(pool* N1 (data or analys*))	
S18	(((systematic or rapid or integrative or umbrella) N3 (review* or overview* or study or studies or search* or approach* or analys*)) or guideline* or recommendations or meta analy* or meta-analy* or metaanaly*)	
S17	PT (Systematic Review or Meta Analysis) or MH ("Systematic Review+" or "Meta Analysis+")	
S16	S4 AND S15	
S15	S5 OR S6 OR S7 OR S8 OR S9 OR S10 OR S11 OR S12 OR S13 OR S14	
S14	((non-pharma* or nonpharma* or non pharma* or non-drug or nondrug or non drug or non-surgical or nonsurgical or non surgical or non-surgery or nonsurgery or non surgery or non-invasive or noninvasive or non invasive or complimentary or integrat* or holistic or multi-modal* or multimodal* or Multidisciplinary or Multi-disciplinary or Interdisciplinary or Inter-disciplinary or complex or multimodal* or multi-modal* or cross-disciplinary or crossdisciplinary or multi-dimensional or multidimensional or biopsychosocial or bio-psycho-social or multi-facet* or multifacet* or comprehensive or multiple or physiotherapy or physical therap*) N3 (therap* or treat* or intervention*	

	or action* or program* or strateg* or protocol* or support* or approach* or evaluation or rehabilitation or care or session*))	
S13	neurorehabilitati* or neuro-rehabilitati*	
S12	(mckenzie or mechanical or Alexander or William or Feldenkrais) N2 (technique* or model* or tool* or exercise* or therapy)	
S11	(yoga or tai ji or tai chi or qigong or qi-gong or pilates*)	
S10	running or Skate* or skating or skiing or Jog* or zumba or kettlebelt or Swim* or Bicycl* or cycling or bicycling or fitness bike* or exercise bike* or spinning or walk* or rowing or rower or water-rower* or waterrower* or cross-train* or crosstrain* or pacing or paced or titreted or (weight* N3 (lift* or train* or exercis*))	
S9	(active rehabilitation or aerobic* or anaerobic* or movement therapy or watergym* or water gym* or aquagym or gym* or hydrotherap* or pilates)	
S8	(muscl* or resistance or physical or endurance or strength* or balance or postur* or gait or threshold* or treadmill* or aquatic* or trunk or graded or gradual or progressive or controlled or incremented or symptom-based or symptombased or symptom based or guided or supervis* or coached or coaching or stepped care or stepwise or step-wise) N3 (train* or retrain* or re-train* or exercise* or rehabilitation or strength* or fit* or condition* or exposure or activit* or exertion or load* or physical therap* or physiotherap*)	
S7	(physical N1 (education or program*)) or (exercise N3 (session* or training or technique* or physical or isometric or therap* or program* or class*))	
S6	TI (exercis* or train* or stepped care or physical therap* or physiotherap* or kinesiotherapy)	
S5	MH "Physical therapy" or MH "Exercise+" or MH "Exercise therapy+" or MH Sports+ or MH "Physical Exertion+" or MH "Muscle strength+" or MH "Exercise Movement Techniques+" or MH "physical therapy modalities+ or MH "Physical Education and Training+" or MH "Weight-Bearing" or MH "Ta ji+" OR MH "Yoga+"	
S4	S1 OR S2 OR S3	
S3	SU (headache* or head ache* or head pain or cephalalgia* or cephalgia* or migraine* or Cephalodynia* or cranial pain or hemicranias* or cerebral pain or cranialgia*)	

S2	(headache* or head ache* or head pain or cephalalgia* or cephalgia* or migraine* or Cephalodynia* or cranial pain or hemicranias* or cerebral pain or cranialgia*)	
S1	MH "Headache" or MH "Headache, Primary" or MH "Migraine" or MH "Tension headache"	

PEDRO

<u>Abstract & Title</u>: headache or migraine*

<u>Method</u>: Practice guideline or Systematic review

Should patients with tension-type headache be offered psychological treatment? Should patients with tension-type headache be offered patient education besides usual treatment?

Combined search

<u>Search date: 20.05.2020</u>

Medline

Database(s): **Ovid MEDLINE(R) and Epub Ahead of Print, In-Process & Other Non-Indexed Citations, Daily and Versions(R)** 1946 to May 19, 2020
Search Strategy:

#	Searches
1	Headache/
2	Headache disorders/
3	Headache Disorders, Primary/
4	exp Migraine Disorders/
5	exp Tension-Type Headache/
6	(headache* or head ache* or head pain or cephalalgia* or cephalgia* or migraine* or Cephalodynia* or cranial pain or hemicranias* or cerebral pain or cranialgia*).ti,bt,ab,kw,kf.
7	or/1-6
8	Health education/
9	exp Patient Education as Topic/
10	exp Programmed Instruction as Topic/
11	Hotlines/
12	exp Counseling/
13	(psychoeducat* or psycho-educat*).ti,bt,ab,kw,kf.
14	((information* or educat* or guid* or counsel?ing or school* or clinic*1 or Course* or Coach* or instruction or class* or advic* or advis* or handout* or hand-out* or pamphlet* or leaflet* or booklet* or brochure* or website) adj3 (health or headache og migraine or treatment* or therapy or intervention or management or patient* or tailor* or individual* or Patient-centred or Patient-centered or Patient-oriented or Patient-focused or Patient-based or Patient-tailored or client-cent* or client cent* or client-focus* or client focus* or Client-oriented or Client-based or Client-tailored or person-centered or person-centred or person centered or person centred or person-focus* or person focus* or Person-oriented or Person-based or Person-tailored or Individual-centred or Individual-centered or Individual-focused or Individual-oriented or Individual-based or Individual-tailored)).ti,bt,ab,kw,kf.

15	(self-management or self-instruction* or selfmanagement or selfinstruction* or self management or self instruction* or self-guid* or self guid* or selfguid* or self care or self-care or selfcare or guidance).ti,bt,ab,kw,kf.
16	((information* or instruction* or educat*) adj3 (material or advic* or advis* or handout* or hand-out* or pamphlet* or leaflet* or booklet* or brochure* or guid* or resource*)).ti,bt,ab,kw,kf.
17	((education* or learn* or training or teach*) adj2 (program* or patient* or consumer* or material* or resource* or aid*)).ti,bt,ab,kw,kf.
18	(patient adj (information or knowledge or website*)).ti,bt,ab,kw,kf.
19	(workshop* or seminar* or ((discussion or support) adj group*)).ti,bt,ab,kw,kf.
20	(factsheet* or fact sheet* or advice line* or advice-line* or hotline or help line* or help-line* or helpline*).ti,bt,ab,kw,kf.
21	((tele* or tele-based or tele-support* or telehealth or tele-health or telemedicine or tele-medicine or internet* or internet-based or internetbased or internet-delivered or internet-support* or internet-assisted or web or webbased or web-based or web based or web-support* or web-assisted or WWW or email* or e-mail or telephone or phone or computer* or computer-based or computerbased or computer-support* or computer-assisted or PC or PCs or PC-based or pc-support* or pc-assisted or skype or skype-based or skype-delivered or skype-assisted or apps or app-based or app based or app-assisted or smartphone* or ipad* or tablet* or IOS or android* or online* or on-line or mobile or ehealth or e-health or e-therap* or distance or remote or video-conferenc* or videoconference* or chatroom* or electronic or digital* or technology or technology-based or technology-delivered or technology-supported or technology-assisted or distance) adj3 (counsel* or support* or guide* or guidance or education or school* or course* or coach* or teach* or session*)).ti,bt,ab,kw,kf.
22	(tele-counsel?ing or tele-education or tele-instruction or tele-session* or tele-class* or tele-course* or tele-guid* or tele-coaching or internet-counsel?ing or internet-education or internet-instruction or internet-session* or internet-class* or internet-course* or internet-coaching or internet-guid* or web-counsel?ing or web-education or web-instruction or web-session* or web-class* or web-course* or web-coaching or web-guid* or e-counsel?ing).ti,bt,ab,kw,kf.
23	exp Psychotherapy/
24	exp Cognitive Behavioral Therapy/
25	exp Adaptation, Psychological/
26	Motivational Interviewing/
27	exp Mindfulness/
28	Breathing Exercises/
29	exp Biofeedback, Psychology/ or feedback/ or feedback, psychological/ or autogenic training/
30	Relaxation therapy/ or Muscle relaxation/
31	"Imagery (Psychotherapy)"/

32	(cbt or act or (acceptance adj2 therapy)).ti,bt,ab,kw,kf.
33	(cognitive-behavio?r-therapy or cognitive-behavio?ral-therapy).ti,bt,ab,kw,kf.
34	((cognitive or cognitive-behavio?r* or behavio?r or individual or group or Schema* or asserti* or network or communit* or family or carer* or parent* or imagery or marital or spous* or Transference* or cognitive or behavio?r* or mentali#ation* or visuali#ation or dynamic or cris#s or emotion* or psychodynamic* or Interpersonal or Conversational or psychiatric or Aversive or Biofeedback or neurofeedback or Desensiti#ation or Relaxation or Feedback or Gestalt or Hypnosis or Music or Narrative or Psychoanalytic or Psychotherapeutic or Association or Reality or Neurolinguistic Programming or NPL or mindful* or meditation or attention control or stress) adj3 (therap* or treatment* or intervention* or management* or model* or program* or modal* or treat* or interview* or training or rehabilitation or neuro-rehabilitation or neurorehabilitation or support* or counseling or counselling or remediation or session* or technique*)).ti,bt,ab,kw,kf.
35	(psychotherap* or psycho-therap* or counsel?ing).ti,bt,ab,kw,kf.
36	((psychologic* or neuro-psycholog* or neuropsycholog* or psychosocial or psycho-social) adj3 (therap* or treat* or intervention* or program* or plan* or approach* or action* or model* or modal* or multimodal* or multi-modal* or interview* or management or training or rehabilitation or neuro-rehabilitation or neurorehabilitation or support* or counseling or counselling or remediation or session* or coaching)).ti,bt,ab,kw,kf.
37	((controlled or paced or therap* or exercise*) adj2 breathing).ti,bt,ab,kw,kf.
38	(respirat* adj2 (training or exercise* or therap*)).ti,bt,ab,kw,kf.
39	(guided adj2 (imagery or visuali*)).ti,bt,ab,kw,kf.
40	(problem solving or coping behavio?r* or social coping or stress management or stress handling or adaptation or adaptive behavio?r*).ti,bt,ab,kw,kf.
41	(empower* or neuro-rehabilitati* or neurorehabilitati*).ti,bt,ab,kw,kf.
42	((non-pharma* or nonpharma* or non pharma* or non-drug or nondrug or non drug or non-surgical or nonsurgical or non surgical or non-surgery or nonsurgery or non surgery or non-invasive or noninvasive or non invasive or complimentary or integrat* or holistic or multi-modal* or multimodal* or Multidisciplinary or Multi-disciplinary or Interdisciplinary or Inter-disciplinary or complex or multimodal* or multi-modal* or cross-disciplinary or crossdisciplinary or multi-dimensional or multidimensional or biopsychosocial or bio-psycho-social or multi-facet* or multifacet* or comprehensive or multiple or addition* or physiotherapy or physical therap*) adj6 (therap* or treat* or intervention* or action* or program* or strateg* or protocol* or support* or approach* or evaluation or rehabilitation or care or session*)).ti,bt,kw,kf.
43	or/8-42
44	7 and 43
45	limit 44 to (guideline or practice guideline or systematic reviews or meta analysis)

46	(((systematic or rapid or integrative or umbrella) adj3 (review* or overview* or study or studies or search* or approach* or analys*)) or guideline* or recommendations or meta analy* or meta-analy* or metaanaly*).ti,bt,ab,kw,kf.
47	((pool* or combin*) adj1 (data or analys*)).ti,ab.
48	(pubmed or medline or embase or cochrane or "web of science" or psycinfo or psychinfo or scopus).ti,ab.
49	cochrane.jw.
50	Network meta-analysis/
51	(((multiple or mixed) adj1 (treatment* or therap*) adj1 comparison*) or (indirect adj1 comparison*)).ti,bt,ab,kw,kf.
52	or/46-51
53	44 and 52
54	45 or 53

Embase

Database(s): **Embase** 1974 to 2020 May 19
Search Strategy:

#	Searches
1	Headache/
2	"headache and facial pain"/
3	exp migraine/
4	exp tension headache/
5	exp chronic daily headache/
6	primary headache/
7	stabbing headache/
8	(headache* or head ache* or head pain or cephalalgia* or cephalgia* or migraine* or Cephalodynia* or cranial pain or hemicranias* or cerebral pain or cranialgia* or trigeminal-autonomic-cephalalgia*).ti,ab,kw.
9	or/1-8
10	Health education/
11	exp Patient Education/
12	exp Psychoeducation/
13	teaching/

14	hotline/
15	counseling/ or anticipatory guidance/ or directive counseling/ or e-counseling/ or motivational interviewing/ or patient counseling/ or patient guidance/
16	(psychoeducat* or psycho-educat*).ti,ab,kw.
17	((information* or educat* or guid* or counsel?ing or school* or clinic*1 or Course* or Coach* or instruction or class* or advic* or advis* or handout* or hand-out* or pamphlet* or leaflet* or booklet* or brochure* or website) adj3 (health or headache og migraine or treatment* or therapy or intervention or management or patient* or tailor* or individual* or Patient-centred or Patient-centered or Patient-oriented or Patient-focused or Patient-based or Patient-tailored or client-cent* or client cent* or client-focus* or client focus* or Client-oriented or Client-based or Client-tailored or person-centered or person-centred or person centered or person centred or person-focus* or person focus* or Person-oriented or Person-based or Person-tailored or Individual-centred or Individual-centered or Individual-focused or Individual-oriented or Individual-based or Individual-tailored)).ti,ab,kw.
18	(self-management or self-instruction* or selfmanagement or selfinstruction* or self management or self instruction* or self-guid* or self guid* or selfguid* or self care or self-care or selfcare or guidance).ti,ab,kw.
19	((information* or instruction* or educat*) adj3 (material or advic* or advis* or handout* or hand-out* or pamphlet* or leaflet* or booklet* or brochure* or guid* or resource* or e-book*)).ti,ab,kw.
20	((education* or learn* or training or teach*) adj2 (program* or patient* or consumer* or material* or resource* or aid*)).ti,ab,kw.
21	(patient adj (information or knowledge or website*)).ti,ab,kw.
22	(workshop* or seminar* or ((discussion or support) adj group*)).ti,ab,kw.
23	(factsheet* or advice line* or advice-line* or hotline or help line* or help-line* or helpline*).ti,ab,kw.
24	((tele* or tele-based or tele-support* or telehealth or tele-health or telemedicine or tele-medicine or internet* or internet-based or internetbased or internet-delivered or internet-support* or internet-assisted or web or webbased or web-based or web based or web-support* or web-assisted or WWW or email* or e-mail or telephone or phone or computer* or computer-based or computerbased or computer-support* or computer-assisted or PC or PCs or PC-based or pc-support* or pc-assisted or skype or skype-based or skype-delivered or skype-assisted or app*1 or app-based or app based or app-assisted or smartphone* or ipad* or tablet* or IOS or android* or online* or on-line or mobile or ehealth or e-health or e-therap* or distance or remote or video-conferenc* or videoconference* or chatroom* or electronic or digital* or technology or technology-based or technology-delivered or technology-supported or technology-assisted or distance) adj3 (counsel* or support* or guide* or guidance or education or school* or course* or coach* or teach* or session*)).ti,ab,kw.
25	(tele-counsel?ing or tele-education or tele-instruction or tele-session* or tele-class* or tele-course* or tele-guid* or tele-coaching or internet-counsel?ing or internet-education or internet-instruction or

	internet-session* or internet-class* or internet-course* or internet-coaching or internet-guid* or web-counsel?ing or web-education or web-instruction or web-session* or web-class* or web-course* or web-coaching or web-guid* or e-counsel?ing).ti,ab,kw.
26	exp Psychotherapy/
27	exp Cognitive Behavioral Therapy/
28	psychological aspect/
29	social adaptation/
30	exp biofeedback/
31	exp relaxation training/
32	exp mindfulness/
33	(cbt or act or (acceptance adj2 therapy)).ti,ab,kw.
34	(cognitive-behavio?r-therapy or cognitive-behavio?ral-therapy).ti,ab,kw.
35	((cognitive or cognitive-behavio?r* or individual or group or Schema* or asserti* or network or communit* or family or carer* or parent* or imagery or marital or spous* or Transference* or cognitive or behavio?r* or mentali#ation* or visuali#ation or dynamic or cris#s or emotion* or psychodynamic* or Interpersonal or Conversational or psychiatric or Aversive or Biofeedback or neurofeedback or Desensiti#ation or Relaxation or Feedback or Gestalt or Hypnosis or Music or Narrative or Psychoanalytic or Psychotherapeutic or Association or Reality or Neurolinguistic Programming or NPL or mindful* or meditation or attention control or stress) adj3 (therap* or treatment* or intervention* or management* or model* or program* or modal* or treat* or interview* or training or rehabilitation or neuro-rehabilitation or neurorehabilitation or support* or counseling or counselling or remediation or session* or technique*)).ti,ab,kw.
36	(psychotherap* or psycho-therap* or counsel?ing).ti,ab,kw.
37	((psychologic* or neuro-psycholog* or neuropsycholog* or psychosocial or psycho-social) adj3 (therap* or treat* or intervention* or program* or plan* or approach* or action* or model* or modal* or multimodal* or multi-modal* or interview* or management or training or rehabilitation or neuro-rehabilitation or neurorehabilitation or support* or counseling or counselling or remediation or session* or coaching)).ti,ab,kw.
38	((controlled or paced or therap* or exercise*) adj2 breathing).ti,ab,kw.
39	(respirat* adj2 (training or exercise* or therap*)).ti,ab,kw.
40	(guided adj2 (imagery or visuali*)).ti,ab,kw.
41	(problem solving or coping behavio?r* or social coping or stress management or stress handling or adaptation or adaptive behavio?r*).ti,ab,kw.
42	(empower* or neuro-rehabilitati* or neurorehabilitati*).ti,ab,kw.
43	((non-pharma* or nonpharma* or non pharma* or non-drug or nondrug or non drug or non-surgical or nonsurgical or non surgical or non-surgery or nonsurgery or non surgery or non-invasive or

	noninvasive or non invasive or complimentary or integrat* or holistic or multi-modal* or multimodal* or Multidisciplinary or Multi-disciplinary or Interdisciplinary or Inter-disciplinary or complex or multimodal* or multi-modal* or cross-disciplinary or crossdisciplinary or multi-dimensional or multidimensional or biopsychosocial or bio-psycho-social or multi-facet* or multifacet* or comprehensive or multiple or addition* or physiotherapy or physical therap*) adj6 (therap* or treat* or intervention* or action* or program* or strateg* or protocol* or support* or approach* or evaluation or rehabilitation or care or session*)).ti,kw.
44	or/10-43
45	9 and 44
46	limit 45 to ("systematic review" or meta analysis)
47	(((systematic or rapid or integrative or umbrella) adj3 (review* or overview* or study or studies or search* or approach* or analys*)) or guideline* or recommendations or meta analy* or meta-analy* or metaanaly*).ti,ab,kw.
48	((pool* or combin*) adj1 (data or analys*)).ti,ab.
49	(pubmed or medline or embase or cochrane or "web of science" or psycinfo or psychinfo or scopus).ti,ab.
50	cochrane.jx.
51	Network meta-analysis/
52	(((multiple or mixed) adj1 (treatment* or therap*) adj1 comparison*) or (indirect adj1 comparison*)).ti,ab,kw.
53	or/47-52
54	45 and 53
55	46 or 54

PsycINFO

Database(s): **APA PsycInfo** 1806 to May Week 3 2020
Search Strategy:

#	Searches
1	exp Headache/
2	(headache* or head ache* or head pain or cephalalgia* or cephalgia* or migraine* or Cephalodynia* or cranial pain or hemicranias* or cerebral pain or cranialgia*).ti,ab,id.
3	or/1-2
4	Health education/
5	exp Client Education/

6	exp Psychoeducation/
7	Programmed Instruction/
8	Computer Assisted Instruction/
9	Individualized Instruction/
10	Distance Education/
11	exp Educational Programs/
12	Hot Line Services/
13	exp Counseling/
14	exp Self-Help Techniques/
15	(psychoeducat* or psycho-educat*).ti,ab,id.
16	((information* or educat* or guid* or counsel?ing or school* or Course* or Coach* or instruction or class* or advic* or advis* or handout* or hand-out* or pamphlet* or leaflet* or booklet* or brochure* or website) adj3 (health or headache og migraine or treatment* or therapy or intervention or management or patient* or tailor* or individual* or Patient-centred or Patient-centered or Patient-oriented or Patient-focused or Patient-based or Patient-tailored or client-cent* or client cent* or client-focus* or client focus* or Client-oriented or Client-based or Client-tailored or person-centered or person-centred or person centered or person centred or person-focus* or person focus* or Person-oriented or Person-based or Person-tailored or Individual-centred or Individual-centered or Individual-focused or Individual-oriented or Individual-based or Individual-tailored)).ti,ab,id.
17	(self-management or self-instruction* or selfmanagement or selfinstruction* or self management or self instruction* or self-guid* or self guid* or selfguid* or self care or self-care or selfcare or guidance).ti,ab,id.
18	((information* or instruction* or educat*) adj3 (material or advic* or advis* or handout* or hand-out* or pamphlet* or leaflet* or booklet* or brochure* or guid* or resource*)).ti,ab,id.
19	((education* or learn* or training or teach*) adj2 (program* or patient* or consumer* or material* or resource* or aid*)).ti,ab,id.
20	(patient adj (information or knowledge or website*)).ti,ab,id.
21	(workshop* or seminar* or ((discussion or support) adj group*)).ti,ab,id.
22	(factsheet* or advice line* or advice-line* or hotline or help line* or help-line* or helpline*).ti,ab,id.
23	((tele* or tele-based or tele-support* or telehealth or tele-health or telemedicine or tele-medicine or internet* or internet-based or internetbased or internet-delivered or internet-support* or internet-assisted or web or webbased or web-based or web based or web-support* or web-assisted or WWW or email* or e-mail or telephone or phone or computer* or computer-based or computerbased or computer-support* or computer-assisted or PC or PCs or PC-based or pc-support* or pc-assisted or skype or skype-based or skype-delivered or skype-assisted or app*1 or app-based or app based or app-assisted or smartphone* or ipad* or tablet* or IOS or android* or online* or on-line or mobile or

	ehealth or e-health or e-therap* or distance or remote or video-conferenc* or videoconference* or chatroom* or electronic or digital* or technology or technology-based or technology-delivered or technology-supported or technology-assisted or distance) adj3 (counsel* or support* or guide* or guidance or education or school* or course* or coach* or teach* or session*)).ti,ab,id.
24	(tele-counsel?ing or tele-education or tele-instruction or tele-session* or tele-class* or tele-course* or tele-guid* or tele-coaching or internet-counsel?ing or internet-education or internet-instruction or internet-session* or internet-class* or internet-course* or internet-coaching or internet-guid* or web-counsel?ing or web-education or web-instruction or web-session* or web-class* or web-course* or web-coaching or web-guid* or e-counsel?ing).ti,ab,id.
25	exp Psychotherapy/
26	exp cognitive therapy/
27	exp Cognitive Techniques/
28	Therapeutic Camps/
29	Psychological stress/
30	Anxiety Management/
31	exp Behavior Modification/
32	"Stress and Coping Measures"/
33	Problem Solving/ or Group Problem solving/
34	psychosocial rehabilitation/
35	Biopsychosocial Approach/
36	Motivational Interviewing/
37	exp Reinforcement/
38	exp Mindfulness/ or mindfulness-based interventions/
39	exp Meditation/
40	exp Biofeedback/ or feedback/
41	Relaxation therapy/ or Muscle relaxation/
42	exp Imagery/
43	Interdisciplinary Treatment Approach/ or Multimodal Treatment Approach/
44	(cbt or act or (acceptance adj2 therapy)).ti,ab,id.
45	(cognitive-behavio?r-therapy or cognitive-behavio?ral-therapy).ti,ab,id.
46	((cognitive or cognitive-behavio?r* or behavio?r or individual or group or Schema* or asserti* or network or communit* or family or carer* or parent* or imagery or marital or spous* or Transference* or cognitive or behavio?r* or mentali#ation* or visuali#ation or dynamic or cris#s or emotion* or psychodynamic* or Interpersonal or Conversational or psychiatric or Aversive or Biofeedback or neurofeedback or Desensiti#ation or Relaxation or Feedback or Gestalt or Hypnosis or Music or Narrative or Psychoanalytic or Psychotherapeutic or Association or Reality or Neurolinguistic

	Programming or NPL or mindful* or meditation or attention control or stress) adj3 (therap* or treatment* or intervention* or management* or model* or program* or modal* or treat* or interview* or training or rehabilitation or neuro-rehabilitation or neurorehabilitation or support* or counseling or counselling or remediation or session* or technique*)).ti,ab,id.
47	(psychotherap* or psycho-therap* or counsel?ing).ti,ab,id.
48	((psychologic* or neuro-psycholog* or neuropsycholog* or psychosocial or psycho-social) adj3 (therap* or treat* or intervention* or program* or plan* or approach* or action* or model* or modal* or multimodal* or multi-modal* or interview* or management or training or rehabilitation or neuro-rehabilitation or neurorehabilitation or support* or counseling or counselling or remediation or session* or coaching)).ti,ab,id.
49	((controlled or paced or therap* or exercise*) adj2 breathing).ti,ab,id.
50	(respirat* adj2 (training or exercise* or therap*)).ti,ab,id.
51	((guided adj2 (imagery or visuali*)) or mindful eating or mindful walking).ti,ab,id.
52	(problem solving or coping behavio?r* or social coping or stress management or stress handling or adaptation or adaptive behavio?r*).ti,ab,id.
53	(empower* or neuro-rehabilitati* or neurorehabilitati* or ((lifestyle or life-style or life style) adj1 (modification* or change*))).ti,ab,id.
54	((non-pharma* or nonpharma* or non pharma* or non-drug or nondrug or non drug or non-surgical or nonsurgical or non surgical or non-surgery or nonsurgery or non surgery or non-invasive or noninvasive or non invasive or complimentary or integrat* or holistic or multi-modal* or multimodal* or Multidisciplinary or Multi-disciplinary or Interdisciplinary or Inter-disciplinary or complex or multimodal* or multi-modal* or cross-disciplinary or crossdisciplinary or multi-dimensional or multidimensional or biopsychosocial or bio-psycho-social or multi-facet* or multifacet* or comprehensive or multiple) adj6 (therap* or treat* or intervention* or action* or program* or strateg* or protocol* or support* or approach* or evaluation or rehabilitation or care or session*)).ti,id.
55	or/4-54
56	3 and 55
57	limit 56 to (systematic reviews or meta analysis)
58	(((systematic or rapid or integrative or umbrella) adj3 (review* or overview* or study or studies or search* or approach* or analys*)) or guideline* or recommendations or meta analy* or meta-analy* or metaanaly*).ti,ab,id.
59	((pool* or combin*) adj1 (data or analys*)).ti,ab.
60	or/58-59
61	56 and 60
62	57 or 61

Cinahl

#	Query	Limiters/Expanders
S46	S40 AND S45	Language: Danish, English, Norwegian, Swedish
S45	S41 OR S42 OR S43 OR S44	
S44	(((multiple or mixed) adj1 (treatment* or therap*) N1 comparison*) or (indirect N1 comparison*))	
S43	(pool* N1 (data or analys*))	
S42	(((systematic or rapid or integrative or umbrella) N3 (review* or overview* or study or studies or search* or approach* or analys*)) or guideline* or recommendations or meta analy* or meta-analy* or metaanaly*)	
S41	PT (Systematic Review or Meta Analysis) or MH ("Systematic Review+" or "Meta Analysis+")	
S40	S4 and S39	
S39	S5 OR S6 OR S7 OR S8 OR S9 OR S10 OR S11 OR S12 OR S13 OR S14 OR S15 OR S16 OR S17 OR S18 OR S19 OR S20 OR S21 OR S22 OR S23 OR S24 OR S25 OR S26 OR S27 OR S28 OR S29 OR S30 OR S31 OR S32 OR S33 OR S34 OR S35 OR S36 OR S37 OR S38	
S38	((non-pharma* or nonpharma* or non pharma* or non-drug or nondrug or non drug or non-surgical or nonsurgical or non surgical or non-surgery or nonsurgery or non surgery or non-invasive or noninvasive or non invasive or complimentary or integrat* or holistic or multi-modal* or multimodal* or Multidisciplinary or Multi-disciplinary or Interdisciplinary or Inter-disciplinary or complex or multimodal* or multi-modal* or cross-disciplinary or crossdisciplinary or multi-dimensional or multidimensional or biopsychosocial or bio-psycho-social or multi-facet* or multifacet* or comprehensive or multiple or physiotherapy or physical therap*) N6 (therap* or treat* or intervention* or action* or program* or strateg* or protocol* or support* or approach* or evaluation or rehabilitation or care or session*))	
S37	(empower* or neuro-rehabilitati* or neurorehabilitati*)	
S36	(problem solving or coping behavio#r* or social coping or stress management or stress handling or adaptation or adaptive behavio#r*)	
S35	(guided N2 (imagery or visuali*))	

S34	(respirat* N2 (training or exercise* or therap*))	
S33	((controlled or paced or therap* or exercise*) N2 breathing)	
S32	((psychologic* or neuro-psycholog* or neuropsycholog* or psychosocial or psycho-social) N3 (therap* or treat* or intervention* or program* or plan* or approach* or action* or model* or modal* or multimodal* or multi-modal* or interview* or management or training or rehabilitation or neuro-rehabilitation or neurorehabilitation or support* or counseling or counselling or remediation or session* or coaching))	
S31	(psychotherap* or psycho-therap* or counsel#ing)	
S30	((cognitive or cognitive-behavio#r* or behavio#r or individual or group or Schema* or asserti* or network or communit* or family or carer* or parent* or imagery or marital or spous* or Transference* or cognitive or behavio#r* or mentali?ation* or visuali?ation or dynamic or cris?s or emotion* or psychodynamic* or Interpersonal or Conversational or psychiatric or Aversive or Biofeedback or neurofeedback or Desensiti?ation or Relaxation or Feedback or Gestalt or Hypnosis or Music or Narrative or Psychoanalytic or Psychotherapeutic or Association or Reality or Neurolinguistic Programming or NPL or mindful* or meditation or attention control or stress) N3 (therap* or treatment* or intervention* or management* or model* or program* or modal* or treat* or interview* or training or rehabilitation or neuro-rehabilitation or neurorehabilitation or support* or counseling or counselling or remediation or session* or technique*))	
S29	(cognitive-behavio#r-therapy or cognitive-behavio#ral-therapy)	
S28	(cbt or act or (acceptance adj2 therapy))	
S27	(MH "Guided Imagery")	
S26	(MH "Biofeedback")	
S25	(MH "Breathing Exercises+")	
S24	(MH "Adaptation, Psychological+")	
S23	(MH "Cognitive Therapy+")	
S22	MH Neuropsychology	
S21	(MH "Support, Psychosocial+") OR (MH "Socioenvironmental Therapy+" or MH "Recreational therapy") or (MH "Rehabilitation, Psychosocial+")	

S20	MH (Psychotherapy+ or Mindfulness+ or Counseling+ or "Relaxation Techniques")	
S19	(tele-counsel#ing or tele-education or tele-instruction or tele-session* or tele-class* or tele-course* or tele-guid* or tele-coaching or internet-counsel#ing or internet-education or internet-instruction or internet-session* or internet-class* or internet-course* or internet-coaching or internet-guid* or web-counsel#ing or web-education or web-instruction or web-session* or web-class* or web-course* or web-coaching or web-guid* or e-counsel#ing)	
S18	((tele* or tele-based or tele-support* or telehealth or tele-health or telemedicine or tele-medicine or internet* or internet-based or internetbased or internet-delivered or internet-support* or internet-assisted or web or webbased or web-based or web based or web-support* or web-assisted or WWW or email* or e-mail or telephone or phone or computer* or computer-based or computerbased or computer-support* or computer-assisted or PC or PCs or PC-based or pc-support* or pc-assisted or skype or skype-based or skype-delivered or skype-assisted or apps or app-based or app based or app-assisted or smartphone* or ipad* or tablet* or IOS or android* or online* or on-line or mobile or ehealth or e-health or e-therap* or distance or remote or video-conferenc* or videoconference* or chatroom* or electronic or digital* or technology or technology-based or technology-delivered or technology-supported or technology-assisted or distance) N3 (counsel* or support* or guide* or guidance or education or school* or course* or coach* or teach* or session*))	
S17	(factsheet* or fact sheet* or advice line* or advice-line* or hotline or help line* or help-line* or helpline*)	
S16	(workshop* or seminar* or ((discussion or support) N1 group*))	
S15	(patient N1 (information or knowledge or website*))	
S14	((education* or learn* or training or teach*) N2 (program* or patient* or consumer* or material* or resource* or aid*))	
S13	((information* or instruction* or educat*) N3 (material or advic* or advis* or handout* or hand-out* or pamphlet* or leaflet* or booklet* or brochure* or guid* or resource*))	
S12	(self-management or self-instruction* or selfmanagement or selfinstruction* or self management or self	

	instruction* or self-guid* or self guid* or selfguid* or self care or self-care or selfcare or guidance)	
S11	((information* or educat* or guid* or counsel#ing or school* or clinic* or Course* or Coach* or instruction or class* or advic* or advis* or handout* or hand-out* or pamphlet* or leaflet* or booklet* or brochure* or website) N3 (health or headache og migraine or treatment* or therapy or intervention or management or patient* or tailor* or individual* or Patient-centred or Patient-centered or Patient-oriented or Patient-focused or Patient-based or Patient-tailored or client-cent* or client cent* or client-focus* or client focus* or Client-oriented or Client-based or Client-tailored or person-centered or person-centred or person centered or person centred or person-focus* or person focus* or Person-oriented or Person-based or Person-tailored or Individual-centred or Individual-centered or Individual-focused or Individual-oriented or Individual-based or Individual-tailored))	
S10	(MH "Psychoeducation")	
S9	(psychoeducat* or psycho-educat*)	
S8	(MH "Counseling") OR (MH "Anticipatory Guidance") OR (MH "Motivational Interviewing") OR (MH "Peer Counseling")	
S7	(MH "Telephone Information Services")	
S6	(MH "Programmed Instruction+")	
S5	MH ("Health education" or "Patient Education" or "Patient Discharge Education" or "Psychoeducation")	
S4	S1 OR S2 OR S3	
S3	SU (headache* or head ache* or head pain or cephalalgia* or cephalgia* or migraine* or Cephalodynia* or cranial pain or hemicranias* or cerebral pain or cranialgia*)	
S2	(headache* or head ache* or head pain or cephalalgia* or cephalgia* or migraine* or Cephalodynia* or cranial pain or hemicranias* or cerebral pain or cranialgia*)	
S1	MH "Headache" or MH "Headache, Primary" or MH "Migraine" or MH "Tension headache"	

PEDRO

Abstract & Title: headache or migraine*

Method: Practice guideline or Systematic review

OT Seeker

Any Field: headache or migraine*
Method: Systematic Review

Should patients with tension-type headache be offered acupuncture?

Search date: 19.05.2020

Medline

Database(s): **Ovid MEDLINE(R) and Epub Ahead of Print, In-Process & Other Non-Indexed Citations, Daily and Versions(R)** 1946 to May 18, 2020
Search Strategy:

#	Searches
1	Headache/
2	Headache disorders/
3	Headache Disorders, Primary/
4	exp Migraine Disorders/
5	exp Tension-Type Headache/
6	(headache* or head ache* or head pain or cephalalgia* or cephalgia* or migraine or Cephalodynia* or cranial pain or hemicranias* or cerebral pain or cranialgia*).ti,bt,ab,kw,kf.
7	or/1-6
8	exp Acupuncture therapy/ or exp Acupuncture/
9	(mox#bustion or acupuncture* or acu-puncture* or needle* or needling or electroacupuncture or electro-acupuncture or acupressure).ti,bt,ab,kw,kf.
10	((Trigger adj2 point*) or (auricul adj2 acupuncture) or (warm adj2 acupuncture) or (dry adj (needle* or needling))).ti,bt,ab,kw,kf.
11	(neuro-rehabilitati* or neurorehabilitati*).ti,bt,ab,kw,kf.
12	((non-pharma* or nonpharma* or non pharma* or non-drug or nondrug or non drug or non-surgical or nonsurgical or non surgical or non-surgery or nonsurgery or non surgery or non-invasive or noninvasive or non invasive or complimentary or integrat* or holistic or multi-modal* or multimodal* or Multidisciplinary or Multi-disciplinary or Interdisciplinary or Inter-disciplinary or complex or multimodal* or multi-modal* or cross-disciplinary or crossdisciplinary or multi-dimensional or multidimensional or biopsychosocial or bio-psycho-social or multi-facet* or multifacet* or comprehensive or multiple or addition* or physiotherapy or physical therap*) adj6 (therap* or treat* or intervention* or action* or program* or strateg* or protocol* or support* or approach* or evaluation or rehabilitation or care or session*)).ti,bt,kw,kf.
13	or/8-12
14	7 and 13
15	limit 14 to (guideline or practice guideline or systematic reviews or meta analysis)

16	(((systematic or rapid or integrative or umbrella) adj3 (review* or overview* or study or studies or search* or approach* or analys*)) or guideline* or recommendations or meta analy* or meta-analy* or metaanaly*).ti,bt,ab,kw,kf.
17	(pool* adj1 (data or analys*)).ti,ab,kw.
18	(pubmed or medline or embase or cochrane or "web of science" or psycinfo or psychinfo or scopus).ti,ab.
19	cochrane.jw.
20	Network meta-analysis/
21	(((multiple or mixed) adj1 (treatment* or therap*) adj1 comparison*) or (indirect adj1 comparison*)).ti,bt,ab,kw,kf.
22	or/16-21
23	14 and 22
24	15 or 23

Embase

Database(s): **Embase** 1974 to 2020 May 18
Search Strategy:

#	Searches
1	Headache/
2	"headache and facial pain"/
3	exp migraine/
4	exp tension headache/
5	exp chronic daily headache/
6	stabbing headache/
7	primary headache/
8	(headache* or head ache* or head pain or cephalalgia* or cephalgia* or migraine or Cephalodynia* or cranial pain or hemicranias* or cerebral pain or cranialgia*).ti,ab,kw.
9	or/1-8
10	exp Acupuncture/
11	(mox#bustion or acupuncture* or acu-puncture* or needle* or needling or electroacupuncture or electro-acupuncture or acupressure).ti,ab,kw.
12	((Trigger adj2 point*) or (auricul adj2 acupuncture) or (warm adj2 acupuncture) or (dry adj (needle* or needling))).ti,ab,kw.
13	(neuro-rehabilitati* or neurorehabilitati*).ti,ab,kw.

14	((non-pharma* or nonpharma* or non pharma* or non-drug or nondrug or non drug or non-surgical or nonsurgical or non surgical or non-surgery or nonsurgery or non surgery or non-invasive or noninvasive or non invasive or complimentary or integrat* or holistic or multi-modal* or multimodal* or Multidisciplinary or Multi-disciplinary or Interdisciplinary or Inter-disciplinary or complex or multimodal* or multi-modal* or cross-disciplinary or crossdisciplinary or multi-dimensional or multidimensional or biopsychosocial or bio-psycho-social or multi-facet* or multifacet* or comprehensive or multiple or addition* or physiotherapy or physical therap*) adj6 (therap* or treat* or intervention* or action* or program* or strateg* or protocol* or support* or approach* or evaluation or rehabilitation or care or session*)).ti,kw.
15	or/10-14
16	9 and 15
17	limit 16 to ("systematic review" or meta analysis)
18	(((systematic or rapid or integrative or umbrella) adj3 (review* or overview* or study or studies or search* or approach* or analys*)) or guideline* or recommendations or meta analy* or meta-analy* or metaanaly*).ti,ab,kw.
19	(pool* adj1 (data or analys*)).ti,ab,kw.
20	(pubmed or medline or embase or cochrane or "web of science" or psycinfo or psychinfo or scopus).ti,ab.
21	cochrane.jx.
22	Network meta-analysis/
23	(((multiple or mixed) adj1 (treatment* or therap*) adj1 comparison*) or (indirect adj1 comparison*)).ti,ab,kw.
24	or/18-23
25	16 and 24
26	17 or 25

Cinahl

#	Query	Limiters/Expanders
S18	S12 AND S17	Language: Danish, English, Norwegian, Swedish
S17	S13 OR S14 OR S15 OR S16	
S16	(((multiple or mixed) adj1 (treatment* or therap*) N1 comparison*) or (indirect N1 comparison*))	

S15	(pool* N1 (data or analys*))	
S14	(((systematic or rapid or integrative or umbrella) N3 (review* or overview* or study or studies or search* or approach* or analys*)) or guideline* or recommendations or meta analy* or meta-analy* or metaanaly*)	
S13	PT (Systematic Review or Meta Analysis) or MH ("Systematic Review+" or "Meta Analysis+")	
S12	S4 AND S11	
S11	S5 OR S6 OR S7 OR S8 OR S9 OR S10	
S10	((non-pharma* or nonpharma* or non pharma* or non-drug or nondrug or non drug or non-surgical or nonsurgical or non surgical or non-surgery or nonsurgery or non surgery or non-invasive or noninvasive or non invasive or complimentary or integrat* or holistic or multi-modal* or multimodal* or Multidisciplinary or Multi-disciplinary or Interdisciplinary or Inter-disciplinary or complex or multimodal* or multi-modal* or cross-disciplinary or crossdisciplinary or multi-dimensional or multidimensional or biopsychosocial or bio-psycho-social or multi-facet* or multifacet* or comprehensive or multiple or physiotherapy or physical therap*) N3 (therap* or treat* or intervention* or action* or program* or strateg* or protocol* or support* or approach* or evaluation or rehabilitation or care or session*))	
S9	((Trigger N2 point*) or (auricul N2 acupuncture) or (warm N2 acupuncture) or (dry N1 (needle* or needling)))	
S8	(mox?bustion or acupuncture* or acu-puncture* or needle* or needling or electroacupuncture or electro-acupuncture or acupressure)	
S7	MH "Dry needling"	
S6	MH Acupressure	
S5	MH "Acupuncture+"	
S4	S1 OR S2 OR S3	
S3	SU (headache* or head ache* or head pain or cephalalgia* or cephalgia* or migraine* or Cephalodynia* or cranial pain or hemicranias* or cerebral pain or cranialgia*)	
S2	(headache* or head ache* or head pain or cephalalgia* or cephalgia* or migraine* or Cephalodynia* or cranial pain or hemicranias* or cerebral pain or cranialgia*)	
S1	MH "Headache" or MH "Headache, Primary" or MH "Migraine" or MH "Tension headache"	

PEDRO

Abstract & Title: headache or migraine*

Method: Practice guideline or Systematic review

Search for randomized controlled trials/controlled trials

Should patients with tension-type headache be offered treatment with manual joint-mobilizing techniques besides usual care?

Search date: 08.07.2020

Medline

Database(s): **Ovid MEDLINE(R) and Epub Ahead of Print, In-Process & Other Non-Indexed Citations, Daily and Versions(R)** 1946 to July 07, 2020
Search Strategy:

#	Searches
1	Headache Disorders, Primary/
2	exp Tension-Type Headache/
3	((tension* or tension-type) adj2 (headache* or head ache*)).ti,bt,ab,kw,kf.
4	or/1-3
5	exp Musculoskeletal Manipulations/
6	Manipulation, Chiropractic/
7	Manipulation, Orthopedic/
8	Manipulation, Osteopathic/
9	Manipulation, Spinal/
10	Osteopathic Medicine/
11	exp Physical therapy modalities/
12	(adjust* adj6 (chiropract* or spinal or spine or lumbar or cervical or neck or thoracic or instrument* or tool* or electric)).ti,bt,ab,kf.
13	(HVLA or high velocity low amplitude).ti,bt,ab,kw.
14	(manipulat* and (chiropract* or naprapath* or osteopath* or oesteopath* or orthopedic* or orthopaedic*)).ti,bt,ab,kf.
15	(manipulat* and (spinal or spine or low* back or joint* or lumbar or neck or thoracic or cervical or cervix or MSK or musculoskeletal or musculo-skeletal or vertebr*)).ti,bt,ab,kf.
16	(manipulat* and (physiotherap* or physical therap*)).ti,bt,ab,kf.
17	(manipulat* and technique*).ti,bt,ab,kw,kf.
18	(mobili#at* and (chiropract* or naprapath* or osteopath* or oesteopath* or orthopedic* or orthopaedic* or physical therap* or physiotherap*)).ti,bt,ab,kf.

19	(mobili#at* and (spinal or spine or low* back or joint* or lumbar or neck or thoracic or cervical or cervix or MSK or musculoskeletal or musculo-skeletal or vertebr*)).ti,bt,ab,kf.
20	((manual or manipulat* or mobili#at* or MSK or movement or musculoskeletal or musculo-skeletal or neurorehabilitati* or neuro-rehabilitati* or physical) adj6 (therap* or treat* or intervention* or action* or program* or strateg* or protocol* or support* or approach* or evaluation)).ti,bt,ab,kf.
21	OMT.ti,bt,ab,kw,kf.
22	((trigger point or motion or passive or cpm) adj2 therap*).ti,bt,ab,kw,kf.
23	(traction adj3 (manual or passive or mechanical or non-surgical or nonsurgical)).ti,bt,ab,kf.
24	(flexion-distraction or flexion distraction).ti,bt,ab,kf.
25	(activator adj3 (method* or technique* or instrument*)).ti,bt,ab,kf.
26	((mechanical or Alexander or Atlas Orthogonal or Atlas Specific or Bilateral Nasal Specific or Blair Upper Cervical or Chiropractic Manipulative Reflex or Cox or Derifield-Thompson or Directional Non-Force or Diversified or Endo-Nasal or feldenkrais or William or Flexion-Distraction or Full Spine or Gonstead or Grostic or Harrison or Koren Specific or Logan Basic or McTimoney or Maitland or Mulligan or muligan or Neuro Emotional or NUCCA or Palmer or Pierce-Stillwagon or Sacro Occipital or Thompson Terminal Point or Toftness or Toggle Recoil or Torque Release or Webster or muscle energy) adj1 (Technique* or model* or tool* or exercise* or therapy)).ti,bt,ab,kw,kf.
27	(reflexolog* or cyriax or High velocity or low amplitude or Subluxation or Joint dysfunction or hypomobility or muscle energy technique* or therapeutic touch or stretching or acupressure or o?steopath*).ti,bt,ab,kw,kf.
28	(vibration adj5 (therap* or treatment*)).ti,bt,ab,kw,kf.
29	((non-pharma* or nonpharma* or non pharma* or non-drug or nondrug or non drug or non-surgical or nonsurgical or non surgical or non-surgery or nonsurgery or non surgery or non-invasive or noninvasive or non invasive or complimentary or integrat* or holistic or multi-modal* or multimodal* or Multidisciplinary or Multi-disciplinary or Interdisciplinary or Inter-disciplinary or complex or multimodal* or multi-modal* or cross-disciplinary or crossdisciplinary or multi-dimensional or multidimensional or biopsychosocial or bio-psycho-social or multi-facet* or multifacet* or comprehensive or multiple or addition* or physiotherapy or physical therap*) adj6 (therap* or treat* or intervention* or action* or program* or strateg* or protocol* or support* or approach* or evaluation or rehabilitation or care or session*)).ti,bt,kw,kf.
30	or/5-29
31	4 and 30
32	limit 31 to (randomized controlled trial or controlled clinical trial)
33	(((random* or cluster-random* or quasi-random* or control?ed or crossover or cross-over or blind* or mask*) adj4 (trial*1 or study or studies or analy*)) or rct).ti,bt,ab,kf.
34	(placebo* or single-blind* or double-blind* or triple-blind*).ti,bt,ab,kf.

35	((single or double or triple) adj2 (blind* or mask*)).ti,bt,ab,kf.
36	((patient* or person* or participant* or population* or allocate* or assign*) adj3 (random* or blind* or mask*)).tw.
37	or/33-36
38	31 and 37
39	32 or 38
40	limit 39 to (yr="2017-2020" and (english or danish or norwegian or swedish))

Embase

Database(s): **Embase** 1974 to 2020 July 07
Search Strategy:

#	Searches
1	exp tension headache/
2	primary headache/
3	((tension* or tension-type) adj2 (headache* or head ache*)).ti,ab,kw.
4	or/1-3
5	exp musculoskeletal manipulation/
6	exp physiotherapy/
7	exp Osteopathy/
8	(adjust* adj6 (chiropract* or spinal or spine or lumbar or cervical or neck or thoracic or instrument* or tool* or electric)).ti,ab,kw.
9	(HVLA or high velocity low amplitude).ti,ab,kw.
10	(manipulat* and (chiropract* or naprapath* or osteopath* or oesteopath* or orthopedic* or orthopaedic*)).ti,ab,kw.
11	(manipulat* and (spinal or spine or low* back or joint* or lumbar or neck or thoracic or cervical or cervix or MSK or musculoskeletal or musculo-skeletal or vertebr*)).ti,ab,kw.
12	(manipulat* and (physiotherap* or physical therap*)).ti,ab,kw.
13	(manipulat* and technique*).ti,ab,kw.
14	(mobili?at* and (chiropract* or naprapath* or osteopath* or orthopedic* or orthopaedic* or physical therap* or physiotherap*)).ti,ab,kw.
15	(mobili?at* and (spinal or spine or low* back or joint* or lumbar or neck or thoracic or cervical or MSK or musculoskeletal or vertebr*)).ti,ab,kw.

16	((manual or manipulat* or mobili?at* or MSK or musculoskeletal or musculo-skeletal or neurorehabilitati* or neuro-rehabilitati* or physical) adj6 (therap* or treat* or intervention* or action* or program* or strateg* or protocol* or support* or approach* or evaluation)).ti,ab,kw.
17	(traction adj3 (manual or passive or mechanical or non-surgical or nonsurgical)).ti,ab,kw.
18	(flexion-distraction or flexion distraction).ti,ab,kw.
19	((activator or physiotherapy or physical therapy) adj3 (method* or technique* or instrument*)).ti,ab,kw.
20	((mechanical or Alexander or Atlas Orthogonal or Atlas Specific or Bilateral Nasal Specific or Blair Upper Cervical or Chiropractic Manipulative Reflex or Cox or Derifield-Thompson or Directional Non-Force or Diversified or Endo-Nasal or feldenkrais or William or Flexion-Distraction or Full Spine or Gonstead or Grostic or Harrison or Koren Specific or Logan Basic or McTimoney or Maitland or Mulligan or muligan or Neuro Emotional or NUCCA or Palmer or Pierce-Stillwagon or Sacro Occipital or Thompson Terminal Point or Toftness or Toggle Recoil or Torque Release or Webster or muscle energy) adj1 (Technique* or model* or tool* or exercise* or therapy)).ti,ab,kw.
21	(reflexolog* or cyriax or High velocity or low amplitude or Subluxation or Joint dysfunction or hypomobility or muscle energy technique* or therapeutic touch or stretching or acupressure or o?steopath*).ti,ab,kw.
22	OMT.ti,ab,kw.
23	(vibration adj5 (therap* or treatment*)).ti,ab,kw.
24	((non-pharma* or nonpharma* or non pharma* or non-drug or nondrug or non drug or non-surgical or nonsurgical or non surgical or non-surgery or nonsurgery or non surgery or non-invasive or noninvasive or non invasive or complimentary or integrat* or holistic or multi-modal* or multimodal* or Multidisciplinary or Multi-disciplinary or Interdisciplinary or Inter-disciplinary or complex or multimodal* or multi-modal* or cross-disciplinary or crossdisciplinary or multi-dimensional or multidimensional or biopsychosocial or bio-psycho-social or multi-facet* or multifacet* or comprehensive or multiple or addition* or physiotherapy or physical therap*) adj6 (therap* or treat* or intervention* or action* or program* or strateg* or protocol* or support* or approach* or evaluation or rehabilitation or care)).ti,kw.
25	or/5-24
26	4 and 25
27	limit 26 to (randomized controlled trial or controlled clinical trial)
28	(((random* or cluster-random* or quasi-random* or control?ed or crossover or cross-over or blind* or mask*) adj4 (trial*1 or study or studies or analy*)) or rct).ti,ab,kw.
29	(placebo* or single-blind* or double-blind* or triple-blind*).ti,ab,kw.
30	((single or double or triple) adj2 (blind* or mask*)).ti,ab,kw.
31	((patient* or person* or participant* or population* or allocate* or assign*) adj3 (random* or blind* or mask*)).ti,ab,kw.

32	or/28-31
33	26 and 32
34	27 or 33
35	limit 34 to (yr="2017-2020" and (danish or english or norwegian or swedish))

Cochrane Central

ID	Search Hits
#1	MeSH descriptor: [Headache Disorders, Primary] this term only
#2	MeSH descriptor: [Tension-Type Headache] explode all trees
#3	((tension* or tension-type) NEAR/2 (headache* or head ache*)):ti,ab,kw
#4	#1 or #2 #3
#5	MeSH descriptor: [Musculoskeletal Manipulations] explode all trees
#6	(adjust* NEAR/6 (chiropract* or spinal or lumbar or cervical or thoracic or instrument* or tool* or electric)):ti,ab,kw
#7	(HVLA or high velocity low amplitude):ti,ab,kw
#8	(manipulat* and (chiropract* or naprapath* or oesteopath* or osteopath* or osteopath* or orthopedic* or orthopaedic*)):ti,ab,kw
#9	(manipulat* and (spinal or spine or low* back or joint* or lumbar or neck or thoracic or cervical or MSK or musculoskeletal or vertebr*)):ti,ab,kw
#10	(manipulat* and (physiotherap* or physical therap*)):ti,ab,kw
#11	(manipulat* and technique*):ti,ab,kw
#12	(mobili?at* and (chiropract* or naprapath* or osteopath* or osteopath* or orthopedic* or orthopaedic*)):ti,ab,kw
#13	((therap* or treat* or intervention* or manag* or rehabilit* or neurorehabilitation or strateg*) NEAR/6 (manual or manipulat* or mobili?at* or MSK or musculoskeletal)):ti,ab,kw
#14	(traction NEAR/3 (manual or passive or mechanical or non-surgical or nonsurgical)):ti,ab,kw
#15	(flexion-distraction or flexion distraction):ti,ab,kw
#16	(activator NEAR/3 (method* or technique* or instrument*)):ti,ab,kw
#17	((mechanical or Alexander or Atlas Orthogonal or Atlas Specific or Bilateral Nasal Specific or Blair Upper Cervical or Chiropractic Manipulative Reflex or Cox or Derifield-Thompson or Directional Non-Force or Diversified or Endo-Nasal or feldenkrais or William or Flexion-Distraction or Full Spine or Gonstead or Grostic or Harrison or Koren Specific or Logan Basic or McTimoney or Maitland or Mulligan or muligan or Neuro Emotional or NUCCA or Palmer or Pierce-Stillwagon or Sacro Occipital or Thompson Terminal Point or Toftness or Toggle Recoil or Torque Release or Webster or muscle energy) NEAR/1 (Technique* or model* or tool* or exercise* or therapy)):ti,ab,kw
#18	(reflexolog* or cyriax or High velocity or low amplitude or Subluxation or Joint dysfunction or hypomobility or muscle energy technique* or therapeutic touch or stretching):ti,ab,kw
#19	#5 or #6 or #7 or #8 or #9 or #10 or #11 or #12 or #13 or #14 or #15 or #16 or #17 or #18
#20	#4 AND #19 with Publication Year from 2017 to 2020, in Trials

---

Cinahl

#	Query	Limiters/Expanders
S22	S16 AND S21	Limiters - Published Date: 20170101-20200731; Language: Danish, English, Norwegian, Swedish
S21	S17 OR S18 OR S19 OR S20	
S20	((patient* or person* or participant* or population* or allocate* or assign*) N3 (random* or blind* or mask*))	
S19	(placebo* or single-blind* or double-blind* or triple-blind* or ((single or double or triple) N1 (blind* or mask*))	
S18	(((random* or cluster-random* or quasi-random* or control#ed or crossover or cross-over or blind* or mask*) N4 (trial* or study or studies or analy*)) or rct)	
S17	PT Randomized Controlled Trial	
S16	S4 AND S15	
S15	S5 OR S6 OR S7 OR S8 OR S9 OR S10 OR S11 OR S12 OR S13 OR S14	
S14	((non-pharma* or nonpharma* or non pharma* or non-drug or nondrug or non drug or non-surgical or nonsurgical or non surgical or non-surgery or nonsurgery or non surgery or non-invasive or noninvasive or non invasive or complimentary or integrat* or holistic or multi-modal* or multimodal* or Multidisciplinary or Multi-disciplinary or Interdisciplinary or Inter-disciplinary or complex or multimodal* or multi-modal* or cross-disciplinary or crossdisciplinary or multi-dimensional or multidimensional or biopsychosocial or bio-psycho-social or multi-facet* or multifacet* or comprehensive or multiple or physiotherapy or physical therap*) N3 (therap* or treat* or intervention* or action* or program* or strateg* or protocol* or support* or approach* or evaluation or rehabilitation or care or session*))	
S13	(vibration N5 (therap* or treatment*))	
S12	reflexolog* or cyriax or High velocity or low amplitude or Subluxation or Joint dysfunction or hypomobility or muscle energy technique* or therapeutic touch or stretching or acupressure or o#steopath*	
S11	((mechanical or Alexander or Atlas Orthogonal or Atlas Specific or Bilateral Nasal Specific or Blair Upper Cervical or Chiropractic Manipulative Reflex or Cox or Derifield-Thompson or Directional	

	Non-Force or Diversified or Endo-Nasal or feldenkrais or William or Flexion-Distraction or Full Spine or Gonstead or Grostic or Harrison or Koren Specific or Logan Basic or McTimoney or Maitland or Mulligan or muligan or Neuro Emotional or NUCCA or Palmer or Pierce-Stillwagon or Sacro Occipital or Thompson Terminal Point or Toftness or Toggle Recoil or Torque Release or Webster or muscle energy or physical therapy or physiotherapy) N1 (Technique* or model* or tool* or exercise* or therapy))	
S10	((manual or manipulat* or mobili?at* or MSK or musculoskeletal or musculo-skeletal or neurorehabilitati* or neuro-rehabilitati* or physical) N6 (therap* or treat* or intervention* or action* or program* or strateg* or protocol* or support* or approach* or evaluation or session*)) or ((traction N3 (manual or passive or mechanical or non-surgical or nonsurgical))) or (activator N3 (method* or technique* or instrument*))	
S9	(mobili?at* and (chiropract* or naprapath* or osteopath* or oesteopath* or orthopedic* or orthopaedic* or spinal or spine or low* back or joint* or lumbar or neck or thoracic or cervical or cervix or MSK or musculoskeletal or musculo-skeletal or vertebr*))	
S8	(manipulat* and (chiropract* or naprapath* or osteopath* or oesteopath* or orthopedic* or orthopaedic* or spinal or spine or low* back or joint* or lumbar or neck or thoracic or cervical or cervix or MSK or musculoskeletal or musculo-skeletal or vertebr* or physiotherap* or physical therap* or technique*))	
S7	HVLA or high velocity low amplitude or flexion-distraction or flexion distraction	
S6	(adjust* N6 (chiropract* or spinal or spine or lumbar or cervical or neck or thoracic or instrument* or tool* or electric))	
S5	MH "Physical therapy" or MH "Manual therapy+" or MH "Joint mobilization"	
S4	S1 OR S2 OR S3	
S3	SU ((tension* or tension-type) N2 headache*)	
S2	(tension* or tension-type) N2 headache*	
S1	MH "Headache, Primary" or MH "Tension Headache"	

PEDRO

<u>Abstract & Title</u>: tension-type headache* or tension headache*

Therapy: stretching, mobilization, manipulation, massage
Method: Clinical Trial
Published since: 2017

Should patients with tension-type headache be offered physical activity?

Search date: 08.07.2020

Medline

Database(s): **Ovid MEDLINE(R) and Epub Ahead of Print, In-Process & Other Non-Indexed Citations, Daily and Versions(R)** 1946 to July 07, 2020
Search Strategy:

#	Searches
1	Headache Disorders, Primary/
2	exp Tension-Type Headache/
3	((tension* or tension-type) adj2 (headache* or head ache*)).ti,bt,ab,kw,kf.
4	or/1-3
5	exp exercise/
6	exp exercise therapy/
7	exp Sports/
8	exp Physical Exertion/
9	exp Muscle strength/
10	exp Exercise Movement Techniques/
11	exp "Physical Education and Training"/
12	Weight-Bearing/
13	Tai Ji/
14	Yoga/
15	Qigong/
16	physical therapy modalities/
17	(exercis* or train* or stepped care or physical therap* or physiotherap* or kinesiotherapy).ti,bt,kw,kf.
18	(physical adj1 (education or program*)).ti,bt,ab,kw,kf.
19	(exercise adj3 (session* or training or technique* or physical or isometric or therap* or program* or class*)).ti,bt,ab,kw,kf.
20	((muscl* or resistance or physical or endurance or strength* or balance or postur* or gait or threshold* or treadmill* or aquatic* or trunk or graded or gradual or progressive or controlled or incremented or symptom-based or symptombased or symptom based or guided or supervis* or coached or coaching or stepped care or stepwise or step-wise) adj3 (train* or retrain* or re-train* or exercise* or rehabilitation or strength* or fit* or condition* or exposure or activit* or exertion or load* or physical therap* or physiotherap*)).ti,bt,ab,kw,kf.

21	(active rehabilitation or aerobic* or anaerobic* or movement therapy or watergym* or water gym* or aquagym or gym* or hydrotherap* or (warm water adj4 (exercis* or train* or rehab*)) or pilates).ti,bt,ab,kw,kf.
22	(running or Skate* or skating or skiing or Jog* or zumba or kettlebelt or Swim* or Bicycl* or cycling or bicycling or fitness bike* or exercise bike* or spinning or walk* or rowing or rower or water-rower* or waterrower* or cross-train* or crosstrain* or pacing or paced or titreted or (weight* adj3 (lift* or train* or exercis*))).ti,bt,ab,kw,kf.
23	(yoga or tai ji or tai chi or qigong or qi-gong or pilates*).ti,bt,ab,kw,kf.
24	((mckenzie or mechanical or Alexander or William or Feldenkrais) adj2 (technique* or model* or tool* or exercise* or therapy)).ti,bt,ab,kw,kf.
25	(neurorehabilitati* or neuro-rehabilitati*).ti,bt,ab,kw,kf.
26	((non-pharma* or nonpharma* or non pharma* or non-drug or nondrug or non drug or non-surgical or nonsurgical or non surgical or non-surgery or nonsurgery or non surgery or non-invasive or noninvasive or non invasive or complimentary or integrat* or holistic or multi-modal* or multimodal* or Multidisciplinary or Multi-disciplinary or Interdisciplinary or Inter-disciplinary or complex or multimodal* or multi-modal* or cross-disciplinary or crossdisciplinary or multi-dimensional or multidimensional or biopsychosocial or bio-psycho-social or multi-facet* or multifacet* or comprehensive or multiple or addition* or physiotherapy or physical therap*) adj6 (therap* or treat* or intervention* or action* or program* or strateg* or protocol* or support* or approach* or evaluation or rehabilitation or care)).ti,bt,kw,kf.
27	or/5-26
28	4 and 27
29	limit 28 to (randomized controlled trial or controlled clinical trial)
30	(((random* or cluster-random* or quasi-random* or control?ed or crossover or cross-over or blind* or mask*) adj4 (trial*1 or study or studies or analy*)) or rct).ti,bt,ab,kf.
31	(placebo* or single-blind* or double-blind* or triple-blind*).ti,ab,kf,hw.
32	((single or double or triple) adj2 (blind* or mask*)).ti,ab,kf,hw.
33	((patient* or person* or participant* or population* or allocate* or assign*) adj3 (random* or blind* or mask*)).ti,ab,kf.
34	or/30-33
35	28 and 34
36	29 or 35
37	limit 36 to (yr="2004-2020" and (danish or english or norwegian or swedish))

Embase

Database(s): **Embase** 1974 to 2020 July 07
Search Strategy:

#	Searches
1	exp tension headache/
2	primary headache/
3	((tension* or tension-type) adj2 (headache* or head ache*)).ti,ab,kw.
4	or/1-3
5	exp exercise/
6	exp kinesiotherapy/
7	exp Physical Exertion/
8	exp Muscle strength/
9	exp Exercise Movement Techniques/
10	exp "Physical Education and Training"/
11	Weight-Bearing/
12	Tai Ji/
13	Yoga/
14	Qigong/
15	physiotherapy/
16	(exercis* or train* or stepped care or physical therap* or physiotherap* or kinesiotherapy).ti,kw.
17	(physical adj1 (education or program*)).ti,ab,kw.
18	(exercise adj3 (session* or training or technique* or physical or isometric or therap* or program* or class*)).ti,ab,kw.
19	((muscl* or resistance or physical or endurance or strength* or balance or postur* or gait or threshold* or treadmill* or aquatic* or trunk or graded or gradual or progressive or controlled or incremented or symptom-based or symptombased or symptom based or guided or supervis* or coached or coaching or stepped care or stepwise or step-wise) adj3 (train* or retrain* or re-train* or exercise* or rehabilitation or strength* or fit* or condition* or exposure or activit* or exertion or load* or physical therap* or physiotherap*)).ti,ab,kw.
20	(active rehabilitation or aerobic* or anaerobic* or movement therapy or watergym* or water gym* or aquagym or gym* or hydrotherap* or (warm water adj4 (exercis* or train* or rehab))).ti,ab,kw.
21	(running or Skate* or skating or skiing or Jog* or zumba or kettlebelt or Swim* or Bicycl* or cycling or bicycling or fitness bike* or exercise bike* or spinning or walk* or rowing or rower or water-rower* or waterrower* or cross-train* or crosstrain* or pacing or paced or titreted or (weight* adj3 (lift* or train* or exercis*))).ti,ab,kw.

22	(yoga or tai ji or tai chi or qigong or qi-gong or pilates).ti,ab,kw.
23	((mckenzie or mechanical or Alexander or William or Feldenkrais) adj2 (technique* or model* or tool* or exercise* or therapy)).ti,ab,kw.
24	(neurorehabilitati* or neuro-rehabilitati*).ti,ab,kw.
25	((non-pharma* or nonpharma* or non pharma* or non-drug or nondrug or non drug or non-surgical or nonsurgical or non surgical or non-surgery or nonsurgery or non surgery or non-invasive or noninvasive or non invasive or complimentary or integrat* or holistic or multi-modal* or multimodal* or Multidisciplinary or Multi-disciplinary or Interdisciplinary or Inter-disciplinary or complex or multimodal* or multi-modal* or cross-disciplinary or crossdisciplinary or multi-dimensional or multidimensional or biopsychosocial or bio-psycho-social or multi-facet* or multifacet* or comprehensive or multiple or addition* or physiotherapy or physical therap*) adj3 (therap* or treat* or intervention* or action* or program* or strateg* or protocol* or support* or approach* or evaluation or rehabilitation or care or session*)).ti,kw.
26	or/5-25
27	4 and 26
28	limit 27 to (randomized controlled trial or controlled clinical trial)
29	(((random* or cluster-random* or quasi-random* or control?ed or crossover or cross-over or blind* or mask*) adj4 (trial*1 or study or studies or analy*)) or rct).ti,ab,kw.
30	(placebo* or single-blind* or double-blind* or triple-blind*).ti,ab,kw.
31	((single or double or triple) adj2 (blind* or mask*)).ti,ab,kw.
32	((patient* or person* or participant* or population* or allocate* or assign*) adj3 (random* or blind* or mask*)).ti,ab,kw.
33	or/29-32
34	27 and 33
35	28 or 34
36	limit 35 to (yr="2004-2020" and (danish or english or norwegian or swedish))

Cochrane Central

ID	Search	Hits
#1	MeSH descriptor: [Headache Disorders, Primary] this term only	
#2	MeSH descriptor: [Tension-Type Headache] explode all trees	
#3	((tension* or tension-type) NEAR/2 (headache* or head ache*)):ti,ab,kw	
#4	#1 or #2 or #3	
#5	MeSH descriptor: [Exercise Therapy] explode all trees	
#6	MeSH descriptor: [Exercise] explode all trees	
#7	MeSH descriptor: [Sports] explode all trees	
#8	MeSH descriptor: [Physical Therapy Modalities] explode all trees	
#9	MeSH descriptor: [Physical Exertion] explode all trees	
#10	MeSH descriptor: [Muscle Strength] explode all trees	
#11	MeSH descriptor: [Exercise Movement Techniques] explode all trees	
#12	MeSH descriptor: [Weight-Bearing] explode all trees	
#13	MeSH descriptor: [Tai Ji] explode all trees	
#14	MeSH descriptor: [Yoga] explode all trees	
#15	(exercis* or train* or stepped care or physical therap* or physiotherap* or kinesiotherapy):ti,kw	
#16	(physical NEAR/1 (education or program*)):ti,ab,kw	
#17	(exercise NEAR/3 (session* or training or technique* or physical or isometric or therap* or program* or class*)):ti,ab,kw	
#18	((muscl* or resistance or physical or endurance or strength* or balance or aquatic* or trunk or graded or gradual or progressive or incremented or symptom-based or symptombased or symptom based or guided or supervis* or stepped care or stepwise or step-wise) NEAR/3 (train* or retrain* or re-train* or exercise* or rehabilitation or strength* or fit* or condition* or exposure or activit* or exertion or load* or activit*)):ti,ab,kw	
#19	(aerobic* or anaerobic* or movement therapy or watergym* or water gym* or aquagym or gym* or (warm water NEAR/4 (exercis* or train* or rehab*))):ti,ab,kw	
#20	(running or Skate* or skating or skiing or Jog* or zumba or kettlebelt or Swim* or Bicycl* or cycling or bicycling or fitness bike* or exercise bike* or spinning or walk* or rowing or rower or water-rower* or waterrower* or cross-train* or crosstrain* or pacing or paced or titreted or (weight* NEAR/3 (lift* or train* or exercis*))):ti,ab,kw	
#21	(yoga or tai ji or tai chi or qigong or qi-gong or pilates):ti,ab,kw	
#22	((mckenzie or mechanical or Alexander or William or Feldenkrais) NEAR/2 (technique* or model* or tool* or exercise* or therapy)):ti,ab,kw	
#23	#5 or #6 or #7 or #8 or #9 or #10 or #11 #12 or #13 or #14 or #15 or #16 or #17 or #18 or #19 or #20 or #21 or #22	
#24	#4 AND #23 with Publication Year from 2004 to 2020, in Trials	

Cinahl

#	Query	Limiters/Expanders
S22	S16 AND S21	Limiters - Published Date: 20040101-20200731; Language: Danish, English, Norwegian, Swedish
S21	S17 OR S18 OR S19 OR S20	
S20	((patient* or person* or participant* or population* or allocate* or assign*) N3 (random* or blind* or mask*))	
S19	(placebo* or single-blind* or double-blind* or triple-blind* or ((single or double or triple) N1 (blind* or mask*))	
S18	(((random* or cluster-random* or quasi-random* or control#ed or crossover or cross-over or blind* or mask*) N4 (trial* or study or studies or analy*)) or rct)	
S17	PT Randomized Controlled Trial	
S16	S4 AND S15	
S15	S5 OR S6 OR S7 OR S8 OR S9 OR S10 OR S11 OR S12 OR S13 OR S14	
S14	((non-pharma* or nonpharma* or non pharma* or non-drug or nondrug or non drug or non-surgical or nonsurgical or non surgical or non-surgery or nonsurgery or non surgery or non-invasive or noninvasive or non invasive or complimentary or integrat* or holistic or multi-modal* or multimodal* or Multidisciplinary or Multi-disciplinary or Interdisciplinary or Inter-disciplinary or complex or multimodal* or multi-modal* or cross-disciplinary or crossdisciplinary or multi-dimensional or multidimensional or biopsychosocial or bio-psycho-social or multi-facet* or multifacet* or comprehensive or multiple or physiotherapy or physical therap*) N3 (therap* or treat* or intervention* or action* or program* or strateg* or protocol* or support* or approach* or evaluation or rehabilitation or care or session*))	
S13	neurorehabilitati* or neuro-rehabilitati*	

S12	(mckenzie or mechanical or Alexander or William or Feldenkrais) N2 (technique* or model* or tool* or exercise* or therapy)	
S11	(yoga or tai ji or tai chi or qigong or qi-gong or pilates*)	
S10	running or Skate* or skating or skiing or Jog* or zumba or kettlebelt or Swim* or Bicycl* or cycling or bicycling or fitness bike* or exercise bike* or spinning or walk* or rowing or rower or water-rower* or waterrower* or cross-train* or crosstrain* or pacing or paced or titreted or (weight* N3 (lift* or train* or exercis*))	
S9	(active rehabilitation or aerobic* or anaerobic* or movement therapy or watergym* or water gym* or aquagym or gym* or hydrotherap* or pilates)	
S8	(muscl* or resistance or physical or endurance or strength* or balance or postur* or gait or threshold* or treadmill* or aquatic* or trunk or graded or gradual or progressive or controlled or incremented or symptom-based or symptombased or symptom based or guided or supervis* or coached or coaching or stepped care or stepwise or step-wise) N3 (train* or retrain* or re-train* or exercise* or rehabilitation or strength* or fit* or condition* or exposure or activit* or exertion or load* or physical therap* or physiotherap*)	
S7	(physical N1 (education or program*)) or (exercise N3 (session* or training or technique* or physical or isometric or therap* or program* or class*))	
S6	TI (exercis* or train* or stepped care or physical therap* or physiotherap* or kinesiotherapy)	
S5	MH "Physical therapy" or MH "Exercise+" or MH "Exercise therapy+" or MH Sports+ or MH "Physical Exertion+" or MH "Muscle strength+" or MH "Exercise Movement Techniques+" or MH "physical therapy modalities+ or MH "Physical Education and Training+" or MH "Weight-Bearing" or MH "Ta ji+" OR MH "Yoga+"	

S4	S1 OR S2 OR S3	
S3	SU (tension* or tension-type) N2 (headache* or head ache*)	
S2	(tension* or tension-type) N2 (headache* or head ache*)	
S1	MH "Headache, Primary" or MH "Tension Headache"	

PEDRO

Abstract & Title: (tension-type headache* or tension headache*) and exercise
Method: Clinical trial
Published since: 2004

Should patients with tension-type headache be offered psychological treatment?

Search date: 10.07.2020

Medline

Database(s): **Ovid MEDLINE(R) and Epub Ahead of Print, In-Process & Other Non-Indexed Citations, Daily and Versions(R)** 1946 to July 08, 2020
Search Strategy:

#	Searches
1	Headache/
2	Headache disorders/
3	Headache Disorders, Primary/
4	
5	exp Tension-Type Headache/
6	(headache* or head ache* or migraine*).ti,bt,ab,kw,kf.
7	or/1-6
8	exp Counseling/
9	(psychoeducat* or psycho-educat*).ti,bt,ab,kw,kf.
10	(self-management or self-instruction* or selfmanagement or selfinstruction* or self management or self instruction* or self-guid* or self guid* or selfguid* or self care or self-care or selfcare or guidance).ti,bt,ab,kw,kf.
11	exp Psychotherapy/
12	exp Cognitive Behavioral Therapy/
13	exp Adaptation, Psychological/
14	Motivational Interviewing/
15	exp Mindfulness/
16	Breathing Exercises/
17	exp Biofeedback, Psychology/ or feedback/ or feedback, psychological/ or autogenic training/
18	Relaxation therapy/ or Muscle relaxation/
19	"Imagery (Psychotherapy)"/
20	(cbt or act or (acceptance adj2 therapy)).ti,bt,ab,kw,kf.
21	(cognitive-behavio?r-therapy or cognitive-behavio?ral-therapy).ti,bt,ab,kw,kf.
22	((cognitive or cognitive-behavio?r* or behavio?r or individual or group or Schema* or asserti* or network or communit* or family or carer* or parent* or imagery or marital or spous* or Transference* or cognitive or behavio?r* or mentali#ation* or visuali#ation or dynamic or cris#s or

	emotion* or psychodynamic* or Interpersonal or Conversational or psychiatric or Aversive or Biofeedback or neurofeedback or Desensiti#ation or Relaxation or Feedback or Gestalt or Hypnosis or Music or Narrative or Psychoanalytic or Psychotherapeutic or Association or Reality or Neurolinguistic Programming or NPL or mindful* or meditation or attention control or stress) adj3 (therap* or treatment* or intervention* or management* or model* or program* or modal* or treat* or interview* or training or rehabilitation or neuro-rehabilitation or neurorehabilitation or support* or counseling or counselling or remediation or session* or technique*)).ti,bt,ab,kw,kf.
23	(psychotherap* or psycho-therap* or counsel?ing).ti,bt,ab,kw,kf.
24	((psychologic* or neuro-psycholog* or neuropsycholog* or psychosocial or psycho-social) adj3 (therap* or treat* or intervention* or program* or plan* or approach* or action* or model* or modal* or multimodal* or multi-modal* or interview* or management or training or rehabilitation or neuro-rehabilitation or neurorehabilitation or support* or counseling or counselling or remediation or session* or coaching)).ti,bt,ab,kw,kf.
25	((controlled or paced or therap* or exercise*) adj2 breathing).ti,bt,ab,kw,kf.
26	(respirat* adj2 (training or exercise* or therap*)).ti,bt,ab,kw,kf.
27	(guided adj2 (imagery or visuali*)).ti,bt,ab,kw,kf.
28	(problem solving or coping behavio?r* or social coping or stress management or stress handling or adaptation or adaptive behavio?r* or motivational interviewing).ti,bt,ab,kw,kf.
29	(counsel?ing or telecounsel?ing or tele-counsel?ing or internet-counsel?ing or webcounsel?ing or web-counsel?ing or videocounsel?ing or video-counsel?ing or coaching or telecoaching or tele-coaching or internet-coaching or webcoaching or web-coaching or videocoaching or video-coaching).ti,bt,ab,kf.
30	empower*.ti,bt,ab,kw,kf.
31	or/8-30
32	7 and 31
33	limit 32 to (randomized controlled trial or controlled clinical trial)
34	(((random* or cluster-random* or quasi-random* or control?ed or crossover or cross-over or blind* or mask*) adj4 (trial*1 or study or studies or analy*)) or rct).ti,bt,ab,kf.
35	(placebo* or single-blind* or double-blind* or triple-blind*).ti,ab,kf,hw.
36	((single or double or triple) adj2 (blind* or mask*)).ti,ab,kf,hw.
37	((patient* or person* or participant* or population* or allocate* or assign*) adj3 (random* or blind* or mask*)).ti,ab,kf.
38	or/34-37
39	32 and 38
40	33 or 39
41	limit 40 to (yr="2017-2020" and (english or danish or norwegian or swedish))

Embase

Database(s): **Embase** 1974 to 2020 July 09
Search Strategy:

#	Searches
1	
2	exp tension headache/
3	exp chronic daily headache/
4	primary headache/
5	stabbing headache/
6	(headache* or head ache* or migraine*).ti,ab,kw.
7	or/1-6
8	exp Psychoeducation/
9	counseling/ or e-counseling/ or motivational interviewing/
10	(psychoeducat* or psycho-educat*).ti,ab,kw.
11	exp Psychotherapy/
12	exp Cognitive Behavioral Therapy/
13	psychological aspect/
14	social adaptation/
15	exp biofeedback/
16	exp relaxation training/
17	exp mindfulness/
18	(acceptance adj2 therapy).ti,ab,kw.
19	(cognitive-behavio?r-therapy or cognitive-behavio?ral-therapy).ti,ab,kw.
20	((cognitive or cognitive-behavio?r* or individual or group or Schema* or asserti* or network or communit* or family or carer* or parent* or imagery or marital or spous* or Transference* or cognitive or behavio?r* or mentali#ation* or visuali#ation or dynamic or cris#s or emotion* or psychodynamic* or Interpersonal or Conversational or psychiatric or Aversive or Biofeedback or neurofeedback or Desensiti#ation or Relaxation or Feedback or Gestalt or Hypnosis or Music or Narrative or Psychoanalytic or Psychotherapeutic or Association or Reality or Neurolinguistic Programming or NPL or mindful* or meditation or attention control or stress) adj3 (therap* or treatment* or intervention* or management* or model* or program* or modal* or treat* or interview* or training or rehabilitation or neuro-rehabilitation or neurorehabilitation or support* or counseling or counselling or remediation or session* or technique*)).ti,ab,kw.
21	(psychotherap* or psycho-therap*).ti,ab,kw.

22	((psychologic* or neuro-psycholog* or neuropsycholog* or psychosocial or psycho-social) adj3 (therap* or treat* or intervention* or program* or plan* or approach* or action* or model* or modal* or multimodal* or multi-modal* or interview* or management or training or rehabilitation or neuro-rehabilitation or neurorehabilitation or support* or counseling or counselling or remediation or session* or coaching)).ti,ab,kw.
23	((controlled or paced or therap* or exercise*) adj2 breathing).ti,ab,kw.
24	(respirat* adj2 (training or exercise* or therap*)).ti,ab,kw.
25	(guided adj2 (imagery or visuali*)).ti,ab,kw.
26	(problem solving or coping behavio?r* or social coping or stress management or stress handling or adaptation or adaptive behavio?r*).ti,ab,kw.
27	(counsel?ing or telecounsel?ing or tele-counsel?ing or internet-counsel?ing or webcounsel?ing or web-counsel?ing or videocounsel?ing or video-counsel?ing or coaching or telecoaching or tele-coaching or internet-coaching or webcoaching or web-coaching or videocoaching or video-coaching).ti,ab,kw.
28	empower*.ti,ab,kw.
29	or/8-28
30	7 and 29
31	limit 30 to (randomized controlled trial or controlled clinical trial)
32	(((random* or cluster-random* or quasi-random* or control?ed or crossover or cross-over or blind* or mask*) adj3 (trial*1 or study or studies or analy*)) or rct).ti,ab,kw.
33	(placebo* or single-blind* or double-blind* or triple-blind*).ti,ab,kw.
34	((single or double or triple) adj2 (blind* or mask*)).ti,ab,kw.
35	((patient* or person* or participant* or population* or allocate* or assign*) adj3 random*).ti,ab,kw.
36	or/32-35
37	30 and 36
38	31 or 37
39	limit 38 to (yr="2017-2020" and (danish or english or norwegian or swedish))

PsycINFO

Database(s): **APA PsycInfo** 1806 to July Week 1 2020
Search Strategy:

#	Searches
1	exp Headache/
2	(headache* or head ache*).ti,ab,id.
3	or/1-2
4	exp Psychoeducation/
5	exp Counseling/
6	(psychoeducat* or psycho-educat*).ti,ab,id.
7	exp Psychotherapy/
8	exp cognitive therapy/
9	exp Cognitive Techniques/
10	Therapeutic Camps/
11	Psychological stress/
12	Anxiety Management/
13	exp Behavior Modification/
14	"Stress and Coping Measures"/
15	Problem Solving/ or Group Problem solving/
16	psychosocial rehabilitation/
17	Biopsychosocial Approach/
18	Motivational Interviewing/
19	exp Reinforcement/
20	exp Mindfulness/ or mindfulness-based interventions/
21	exp Meditation/
22	exp Biofeedback/ or feedback/
23	Relaxation therapy/ or Muscle relaxation/
24	exp Imagery/
25	Interdisciplinary Treatment Approach/ or Multimodal Treatment Approach/
26	(cbt or act or (acceptance adj2 therapy)).ti,ab,id.
27	(cognitive-behavio?r-therapy or cognitive-behavio?ral-therapy).ti,ab,id.
28	((cognitive or cognitive-behavio?r* or behavio?r or individual or group or Schema* or asserti* or network or communit* or family or carer* or parent* or imagery or marital or spous* or Transference* or cognitive or behavio?r* or mentali#ation* or visuali#ation or dynamic or cris#s or emotion* or psychodynamic* or Interpersonal or Conversational or psychiatric or Aversive or

	Biofeedback or neurofeedback or Desensiti#ation or Relaxation or Feedback or Gestalt or Hypnosis or Music or Narrative or Psychoanalytic or Psychotherapeutic or Association or Reality or Neurolinguistic Programming or NPL or mindful* or meditation or attention control or stress) adj3 (therap* or treatment* or intervention* or management* or model* or program* or modal* or treat* or interview* or training or rehabilitation or neuro-rehabilitation or neurorehabilitation or support* or counseling or counselling or remediation or session* or technique*)).ti,ab,id.
29	(psychotherap* or psycho-therap* or counsel?ing).ti,ab,id.
30	((psychologic* or neuro-psycholog* or neuropsycholog* or psychosocial or psycho-social) adj3 (therap* or treat* or intervention* or program* or plan* or approach* or action* or model* or modal* or multimodal* or multi-modal* or interview* or management or training or rehabilitation or neuro-rehabilitation or neurorehabilitation or support* or counseling or counselling or remediation or session* or coaching)).ti,ab,id.
31	((controlled or paced or therap* or exercise*) adj2 breathing).ti,ab,id.
32	(respirat* adj2 (training or exercise* or therap*)).ti,ab,id.
33	((guided adj2 (imagery or visuali*)) or mindful eating or mindful walking).ti,ab,id.
34	(problem solving or coping behavio?r* or social coping or stress management or stress handling or adaptation or adaptive behavio?r* or motivational interviewing).ti,ab,id.
35	(counsel?ing or telecounsel?ing or tele-counsel?ing or internet-counsel?ing or webcounsel?ing or web-counsel?ing or videocounsel?ing or video-counsel?ing or coaching or telecoaching or tele-coaching or internet-coaching or webcoaching or web-coaching or videocoaching or video-coaching).ti,ab,id.
36	(empower* or neuro-rehabilitati* or neurorehabilitati* or ((lifestyle or life-style or life style) adj1 (modification* or change*))).ti,ab,id.
37	or/4-36
38	3 and 37
39	(((random* or cluster-random* or quasi-random* or control?ed or crossover or cross-over or blind* or mask*) adj4 (trial*1 or study or studies or analy*)) or rct).ti,ab,id.
40	(placebo* or single-blind* or double-blind* or triple-blind* or ((single or double or triple) adj2 (blind* or mask*))).ti,ab,id.
41	((patient* or person* or participant* or population* or allocate* or assign*) adj3 (random* or blind* or mask*)).ti,ab,id.
42	or/39-41
43	38 and 42
44	limit 43 to (yr="2017-2020" and (english or danish or norwegian or swedish))

Cochrane Central

ID	Search	Hits
#1	MeSH descriptor: [Headache] this term only	
#2	MeSH descriptor: [Headache Disorders] this term only	
#3	MeSH descriptor: [Headache Disorders, Primary] this term only	
#4		
#5	MeSH descriptor: [Tension-Type Headache] explode all trees	
#6	(headache* or head ache*):ti,kw	
#7	#1 or #2 or #3 or #4 or #5 or #6	
#8	MeSH descriptor: [Counseling] explode all trees	
#9	(psychoeducat* or psycho-educat*):ti,ab,kw	
#10	MeSH descriptor: [Psychotherapy] explode all trees	
#11	MeSH descriptor: [Mindfulness] explode all trees	
#12	MeSH descriptor: [Cognitive Behavioral Therapy] explode all trees	
#13	MeSH descriptor: [Motivational Interviewing] explode all trees	
#14	MeSH descriptor: [Adaptation, Psychological] explode all trees	
#15	MeSH descriptor: [Breathing Exercises] this term only	
#16	MeSH descriptor: [Biofeedback, Psychology] explode all trees	
#17	MeSH descriptor: [Relaxation Therapy] explode all trees	
#18	MeSH descriptor: [Imagery, Psychotherapy] this term only	
#19	(cbt or (acceptance NEAR/2 therapy)):ti,ab,kw	
#20	(cognitive-behavio?r-therapy or cognitive-behavio?ral-therapy):ti,ab,kw	
#21	((cognitive or cognitive-behavio*r* or cognitive-behavio*r* individual or Schema* or asserti* or network or communit* or family or imagery or marital or spous* or Transference* or cognitive or behavio*r* or mentali?ation* or dynamic or cris?s or emotion* or problem solving or problem-solving or psychodynamic* or Interpersonal or Conversational or psychiatric or Aversive or Biofeedback or Desensiti?ation or Relaxation or Feedback or Gestalt or Hypnosis or Music or Narrative or Psychoanalytic or Psychotherapeutic or Association or Reality or Neurolinguistic Programming or NPL or mindful* or meditation) NEAR/3 (therap* or treatment* or intervention* or management* or model* or program* or treat* or training or rehabilitation or neuro-rehabilitation or neurorehabilitation or counseling or counselling or remediation or session* or coaching)):ti,ab,kw	
#22	(psychotherap* or psycho-therap*):ti,ab,kw	
#23	((Psychosocial or psycho-social or psychologic* or neuro-psycholog* or neuropsycholog*) NEAR/3 (therap* or treatment* or intervention* or management* or model* or program* or treat* or training or rehabilitation or neuro-rehabilitation or neurorehabilitation or support* or counseling or counselling or remediation or session* or coaching)):ti,ab,kw	
#24	((controlled or paced or therap* or exercise*) NEAR/2 breathing):ti,ab,kw	
#25	(respirat* NEAR/2 (training or exercise* or therap*)):ti,ab,kw	
#26	(guided NEAR/2 (imagery or visuali*)):ti,ab,kw	
#27	(problem solving or coping behavior* or coping behaviour or social coping or stress management or stress handling or adaptation or adaptive behavior* or adaptive behaviour* or motivational interviewing):ti,ab,kw	
#28	empower*:ti,ab,kw	

#29	(counsel?ing or telecounsel?ing or tele-counsel?ing or internet-counsel?ing or webcounsel?ing or web-counsel?ing or videocounsel?ing or video-counsel?ing or coaching or telecoaching or tele-coaching or internet-coaching or webcoaching or web-coaching or videocoaching or video-coaching):ti,ab,kw
#30	#8 or #9 or #10 or #11 or #12 or #13 or #14 or #15 or #16 or #17 or #18 or #19 or #20 or #21 or #22 or #23 or #24 or #25 or #26 or #27 or #28 or #29
#31	#7 AND #30 with Publication Year from 2017 to 2020, in Trials

---

Cinahl

#	Query	Limiters/Expanders
S34	S28 AND S33	Limiters - Published Date: 20170101-20200731; Language: Danish, English, Norwegian, Swedish
S33	S29 OR S30 OR S31 OR S32	
S32	((patient* or person* or participant* or population* or allocate* or assign*) N3 (random* or blind* or mask*))	
S31	(placebo* or single-blind* or double-blind* or triple-blind* or ((single or double or triple) N1 (blind* or mask*))	
S30	(((random* or cluster-random* or quasi-random* or control#ed or crossover or cross-over or blind* or mask*) N4 (trial* or study or studies or analy*)) or rct)	
S29	PT Randomized Controlled Trial	
S28	S4 and S27	
S27	S5 OR S6 OR S7 OR S8 OR S9 OR S10 OR S11 OR S12 OR S13 OR S14 OR S15 OR S16 OR S17 OR S18 OR S19 OR S20 OR S21 OR S22 OR S23 OR S24 OR S25 OR S26	
S26	(empower*)	
S25	(problem solving or coping behavio#r* or social coping or stress management or stress handling or adaptation or adaptive behavio#r* or motivational interviewing)	
S24	(guided N2 (imagery or visuali*))	
S23	(respirat* N2 (training or exercise* or therap*))	
S22	((controlled or paced or therap* or exercise*) N2 breathing)	
S21	((psychologic* or neuro-psycholog* or neuropsycholog* or psychosocial or psycho-social) N3 (therap* or treat* or	

	intervention* or program* or plan* or approach* or action* or model* or modal* or multimodal* or multi-modal* or interview* or management or training or rehabilitation or neuro-rehabilitation or neurorehabilitation or support* or counseling or counselling or remediation or session* or coaching))	
S20	(psychotherap* or psycho-therap* or counsel#ing)	
S19	((cognitive or cognitive-behavio#r* or behavio#r or individual or group or Schema* or asserti* or network or communit* or family or carer* or parent* or imagery or marital or spous* or Transference* or cognitive or behavio#r* or mentali?ation* or visuali?ation or dynamic or cris?s or emotion* or psychodynamic* or Interpersonal or Conversational or psychiatric or Aversive or Biofeedback or neurofeedback or Desensiti?ation or Relaxation or Feedback or Gestalt or Hypnosis or Music or Narrative or Psychoanalytic or Psychotherapeutic or Association or Reality or Neurolinguistic Programming or NPL or mindful* or meditation or attention control or stress) N3 (therap* or treatment* or intervention* or management* or model* or program* or modal* or treat* or interview* or training or rehabilitation or neuro-rehabilitation or neurorehabilitation or support* or counseling or counselling or remediation or session* or technique*))	
S18	(cognitive-behavio#r-therapy or cognitive-behavio#ral-therapy)	
S17	(cbt or act or (acceptance adj2 therapy))	
S16	(MH "Guided Imagery")	
S15	(MH "Biofeedback")	
S14	(MH "Breathing Exercises+")	
S13	(MH "Adaptation, Psychological+")	
S12	(MH "Cognitive Therapy+")	
S11	MH Neuropsychology	
S10	(MH "Support, Psychosocial+") OR (MH "Socioenvironmental Therapy+" or MH "Recreational therapy") or (MH "Rehabilitation, Psychosocial+")	
S9	MH (Psychotherapy+ or Mindfulness+ or Counseling+ or "Relaxation Techniques")	
S8	(counsel#ing or telecounsel#ing or tele-counsel#ing or internet-counsel#ing or webcounsel#ing or web-counsel#ing or videocounsel#ing or video-counsel#ing or coaching or telecoaching or tele-coaching or internet-coaching or	

	webcoaching or web-coaching or videocoaching or video-coaching)	
S7	(MH "Psychoeducation")	
S6	(psychoeducat* or psycho-educat*)	
S5	(MH "Counseling") OR (MH "Anticipatory Guidance") OR (MH "Motivational Interviewing") OR (MH "Peer Counseling")	
S4	S1 OR S2 OR S3	
S3	SU (headache* or head ache*)	
S2	(headache* or head ache*)	
S1	MH "Headache" or MH "Headache, Primary"	

PEDRO

Abstract & Title: headache*
Method: Clinical trial
Published since: 2017

OT Seeker

Any Field: headache or migraine*
Method: Randomized Controlled Trial
Year published: 2017-2020

Should patients with tension-type headache be offered acupuncture?

Search date: 19.05.2020

Medline

Søgt 13.07.2020

Database(s): **Ovid MEDLINE(R) and Epub Ahead of Print, In-Process & Other Non-Indexed Citations, Daily and Versions(R)** 1946 to July 10, 2020
Search Strategy:

#	Searches
1	Headache Disorders, Primary/
2	exp Tension-Type Headache/
3	((tension* or tension-type) adj2 (headache* or head ache*)).ti,bt,ab,kw,kf.
4	or/1-3
5	exp Acupuncture therapy/
6	exp Acupuncture/
7	(mox#bustion or acupuncture* or acu-puncture* or needle* or needling or electroacupuncture or electro-acupuncture or acupressure).ti,bt,ab,kw,kf.
8	((Trigger adj2 point*) or (auricul adj2 acupuncture) or (warm adj2 acupuncture) or (dry adj (needle* or needling))).ti,bt,ab,kw,kf.
9	or/5-8
10	4 and 9
11	limit 10 to (randomized controlled trial or controlled clinical trial)
12	(((random* or cluster-random* or quasi-random* or control?ed or crossover or cross-over or blind* or mask*) adj4 (trial*1 or study or studies or analy*)) or rct).ti,bt,ab,kf.
13	(placebo* or single-blind* or double-blind* or triple-blind*).ti,bt,ab,kf.
14	((single or double or triple) adj2 (blind* or mask*)).ti,bt,ab,kf.
15	((patient* or person* or participant* or population* or allocate* or assign*) adj3 (random* or blind* or mask*)).tw.
16	or/12-15
17	10 and 16
18	11 or 17
19	limit 18 to (yr="2016-2020" and (english or danish or norwegian or swedish))
20	limit 19 to (systematic review or review or letter)
21	19 not 20

Embase

Database(s): **Embase** 1974 to 2020 July 10
Search Strategy:

#	Searches
1	exp tension headache/
2	primary headache/
3	((tension* or tension-type) adj2 (headache* or head ache*)).ti,ab,kw.
4	or/1-3
5	exp Acupuncture/
6	(mox#bustion or acupuncture* or acu-puncture* or needle* or needling or electroacupuncture or electro-acupuncture or acupressure).ti,ab,kw.
7	((Trigger adj2 point*) or (auricul adj2 acupuncture) or (warm adj2 acupuncture) or (dry adj (needle* or needling))).ti,ab,kw.
8	or/5-7
9	4 and 8
10	limit 9 to (randomized controlled trial or controlled clinical trial)
11	(((random* or cluster-random* or quasi-random* or control?ed or crossover or cross-over or blind* or mask*) adj4 (trial*1 or study or studies or analy*)) or rct).ti,ab,kw.
12	(placebo* or single-blind* or double-blind* or triple-blind*).ti,ab,kw.
13	((single or double or triple) adj2 (blind* or mask*)).ti,ab,kw.
14	((patient* or person* or participant* or population* or allocate* or assign*) adj3 (random* or blind* or mask*)).ti,ab,kw.
15	or/11-14
16	9 and 15
17	10 or 16
18	limit 17 to (yr="2016-2020" and (danish or english or norwegian or swedish))
19	limit 18 to ("systematic review" or review or letter or note)
20	18 not 19

Cochrane Central

ID	Search	Hits
#1	MeSH descriptor: [Headache Disorders, Primary] this term only	
#2	MeSH descriptor: [Tension-Type Headache] explode all trees	
#3	((tension* or tension-type) NEAR/2 (headache* or head ache*)):ti,ab,kw	
#4	#1 or #2 or #3	
#5	MeSH descriptor: [Acupuncture Therapy] explode all trees	
#6	MeSH descriptor: [Acupuncture] explode all trees	
#7	(mox*bustion or acupuncture* or acu-puncture* or needle* or needling or electroacupuncture or electro-acupuncture or acupressure):ti,ab,kw	
#8	((Trigger NEAR/2 point*) or (auricul NEAR/2 acupuncture) or (warm NEAR/2 acupuncture) or (dry NEAR/1 (needle* or needling)))	
#9	#5 or #6 or #7 or #8	
#10	#4 AND #9 with Publication Year from 2016 to 2020, in Trials	

Cinahl

#	Query	Limiters/Expanders
S17	S11 AND S16	Limiters - Published Date: 20160101-20200731; Language: Danish, English, Norwegian, Swedish
S16	S12 OR S13 OR S14 OR S15	
S15	((patient* or person* or participant* or population* or allocat* or assign*) N3 (random* or blind* or mask*))	
S14	(placebo* or single-blind* or double-blind* or triple-blind* or ((single or double or triple) N1 (blind* or mask*))	
S13	(((random* or cluster-random* or quasi-random* or control#ed or crossover or cross-over or blind* or mask*) N4 (trial* or study or studies or analy*)) or rct)	
S12	PT Randomized Controlled Trial	
S11	S4 AND S10	
S10	S5 OR S6 OR S7 OR S8 OR S9	
S9	((Trigger N2 point*) or (auricul N2 acupuncture) or (warm N2 acupuncture) or (dry N1 (needle* or needling)))	
S8	(mox?bustion or acupuncture* or acu-puncture* or needle* or needling or electroacupuncture or electro-acupuncture or acupressure)	

S7	MH "Dry needling"	
S6	MH Acupressure	
S5	MH "Acupuncture+"	
S4	S1 OR S2 OR S3	
S3	SU (tension* or tension-type) N2 (headache* or head ache*)	
S2	(tension* or tension-type) N2 (headache* or head ache*)	
S1	MH "Headache, Primary" or MH "Tension headache"	

PEDRO

Abstract & Title: tension-type headache* or tension headache*
Therapy: Acupuncture
Method: Clinical trial
Published since: 2016

Should patients with tension-type headache be offered patient education besides usual treatment?

Search date: 14.07.2020

Medline

Database(s): **Ovid MEDLINE(R) and Epub Ahead of Print, In-Process & Other Non-Indexed Citations, Daily and Versions(R)** 1946 to July 10, 2020
Search Strategy:

#	Searches
1	Headache/
2	Headache disorders/
3	Headache Disorders, Primary/
4	
5	exp Tension-Type Headache/
6	(headache* or head ache*).ti,bt,ab,kw,kf.
7	or/1-6
8	Health education/
9	exp Patient Education as Topic/
10	exp Programmed Instruction as Topic/
11	Hotlines/
12	exp Counseling/
13	(psychoeducat* or psycho-educat*).ti,bt,ab,kw,kf.
14	((information* or educat* or guid* or counsel?ing or school* or Course* or Coach* or instruction or class* or advic* or advis* or handout* or hand-out* or pamphlet* or leaflet* or booklet* or brochure* or website) adj3 (health or headache og migraine or treatment* or therapy or intervention or management or patient* or tailor* or individual* or Patient-centred or Patient-centered or Patient-oriented or Patient-focused or Patient-based or Patient-tailored or client-cent* or client cent* or client-focus* or client focus* or Client-oriented or Client-based or Client-tailored or person-centered or person-centred or person centered or person centred or person-focus* or person focus* or Person-oriented or Person-based or Person-tailored or Individual-centred or Individual-centered or Individual-focused or Individual-oriented or Individual-based or Individual-tailored)).ti,bt,ab,kw,kf.
15	(self-management or self-instruction* or selfmanagement or selfinstruction* or self management or self instruction* or self-guid* or self guid* or selfguid* or self care or self-care or selfcare or guidance).ti,bt,ab,kw,kf.

16	((information* or instruction* or educat*) adj3 (material or advic* or advis* or handout* or hand-out* or pamphlet* or leaflet* or booklet* or brochure* or guid* or resource* or e-book*)).ti,bt,ab,kw,kf.
17	((education* or learn* or training or teach*) adj2 (program* or patient* or consumer* or material* or resource* or aid*)).ti,bt,ab,kw,kf.
18	(patient adj (information or knowledge or website*)).ti,bt,ab,kw,kf.
19	(workshop* or seminar* or ((discussion or support) adj group*)).ti,bt,ab,kw,kf.
20	(factsheet* or advice line* or advice-line* or hotline or help line* or help-line* or helpline*).ti,bt,ab,kw,kf.
21	((tele* or tele-based or tele-support* or telehealth or tele-health or telemedicine or tele-medicine or internet* or internet-based or internetbased or internet-delivered or internet-support* or internet-assisted or web or webbased or web-based or web based or web-support* or web-assisted or WWW or email* or e-mail or telephone or phone or computer* or computer-based or computerbased or computer-support* or computer-assisted or PC or PCs or PC-based or pc-support* or pc-assisted or skype or skype-based or skype-delivered or skype-assisted or app*1 or app-based or app based or app-assisted or smartphone* or ipad* or tablet* or IOS or android* or online* or on-line or mobile or ehealth or e-health or e-therap* or distance or remote or video-conferenc* or videoconference* or chatroom* or electronic or digital* or technology or technology-based or technology-delivered or technology-supported or technology-assisted or distance) adj3 (counsel* or support* or guide* or guidance or education or school* or course* or coach* or teach* or session* or information)).ti,bt,ab,kw,kf.
22	(tele-counsel?ing or tele-education or tele-instruction or tele-session* or tele-class* or tele-course* or tele-guid* or tele-coaching or internet-counsel?ing or internet-education or internet-instruction or internet-session* or internet-class* or internet-course* or internet-coaching or internet-guid* or web-counsel?ing or web-education or web-instruction or web-session* or web-class* or web-course* or web-coaching or web-guid* or e-counsel?ing).ti,bt,ab,kw,kf.
23	or/8-22
24	7 and 23
25	limit 24 to (randomized controlled trial or controlled clinical trial)
26	(((random* or cluster-random* or quasi-random* or control?ed or crossover or cross-over or blind* or mask*) adj4 (trial*1 or study or studies or analy*)) or rct).ti,bt,ab,kf.
27	(placebo* or single-blind* or double-blind* or triple-blind*).ti,ab,kf,hw.
28	((single or double or triple) adj2 (blind* or mask*)).ti,ab,kf,hw.
29	((patient* or person* or participant* or population* or allocate* or assign*) adj3 (random* or blind* or mask*)).ti,ab,kf.
30	or/26-29
31	24 and 30

32	25 or 31
33	limit 32 to (yr="2004-2020" and (english or danish or norwegian or swedish))

Embase

Database(s): **Embase** 1974 to 2020 July 13
Search Strategy:

#	Searches
1	Headache/
2	"headache and facial pain"/
3	
4	exp tension headache/
5	exp chronic daily headache/
6	primary headache/
7	stabbing headache/
8	(headache* or head ache*).ti,ab,kw.
9	or/1-8
10	Health education/
11	exp Patient Education/
12	exp Psychoeducation/
13	hotline/
14	counseling/ or anticipatory guidance/ or directive counseling/ or e-counseling/ or patient counseling/ or patient guidance/
15	(psychoeducat* or psycho-educat*).ti,ab,kw.
16	((information* or educat* or guid* or counsel?ing or school* or Course* or Coach* or instruction or class* or advic* or advis* or handout* or hand-out* or pamphlet* or leaflet* or booklet* or brochure* or website) adj3 (health or headache og migraine or treatment* or therapy or intervention or management or patient* or tailor* or individual* or Patient-centred or Patient-centered or Patient-oriented or Patient-focused or Patient-based or Patient-tailored or client-cent* or client cent* or client-focus* or client focus* or Client-oriented or Client-based or Client-tailored or person-centered or person-centred or person centered or person centred or person-focus* or person focus* or Person-oriented or Person-based or Person-tailored or Individual-centred or Individual-centered or Individual-focused or Individual-oriented or Individual-based or Individual-tailored)).ti,ab,kw.
17	(self-management or self-instruction* or selfmanagement or selfinstruction* or self management or self instruction* or self-guid* or self guid* or selfguid* or self care or self-care or selfcare).ti,ab,kw.

18	((information* or instruction* or educat*) adj3 (material or advic* or advis* or handout* or hand-out* or pamphlet* or leaflet* or booklet* or brochure* or guid* or resource* or e-book*)).ti,ab,kw.
19	((education* or learn* or training or teach*) adj2 (program* or patient* or consumer* or material* or resource* or aid or aids)).ti,ab,kw.
20	(patient adj (information or knowledge or website*)).ti,ab,kw.
21	(workshop* or seminar* or ((discussion or support) adj group*)).ti,ab,kw.
22	(factsheet* or advice line* or advice-line* or hotline or help line* or help-line* or helpline*).ti,ab,kw.
23	((tele* or tele-based or tele-support* or telehealth or tele-health or telemedicine or tele-medicine or internet* or internet-based or internetbased or internet-delivered or internet-support* or internet-assisted or web or webbased or web-based or web based or web-support* or web-assisted or WWW or email* or e-mail or telephone or phone or computer* or computer-based or computerbased or computer-support* or computer-assisted or PC or PCs or PC-based or pc-support* or pc-assisted or skype or skype-based or skype-delivered or skype-assisted or app*1 or app-based or app based or app-assisted or smartphone* or ipad* or tablet* or IOS or android* or online* or on-line or mobile or ehealth or e-health or e-therap* or distance or remote or video-conferenc* or videoconference* or chatroom* or electronic or digital* or technology or technology-based or technology-delivered or technology-supported or technology-assisted or distance) adj3 (counsel* or support* or guide* or guidance or education or school* or course* or coach* or teach* or session* or information)).ti,ab,kw.
24	(counsel?ing or tele-counsel?ing or tele-education or tele-instruction or tele-session* or tele-class* or tele-course* or tele-guid* or tele-coaching or internet-counsel?ing or internet-education or internet-instruction or internet-session* or internet-class* or internet-course* or internet-coaching or internet-guid* or web-counsel?ing or web-education or web-instruction or web-session* or web-class* or web-course* or web-coaching or web-guid* or e-counsel?ing).ti,ab,kw.
25	or/10-24
26	9 and 25
27	limit 26 to (randomized controlled trial or controlled clinical trial)
28	(((random* or cluster-random* or quasi-random* or control?ed or crossover or cross-over or blind* or mask*) adj3 (trial*1 or study or studies or analy*)) or rct).ti,ab,kw.
29	(placebo* or single-blind* or double-blind* or triple-blind*).ti,ab,kw.
30	((single or double or triple) adj2 (blind* or mask*)).ti,ab,kw.
31	((patient* or person* or participant* or population* or allocat* or assign*) adj3 random*).ti,ab,kw.
32	or/28-31
33	26 and 32
34	27 or 33
35	limit 34 to (yr="2004-2020" and (danish or english or norwegian or swedish))

PsycINFO

Database(s): **APA PsycInfo** 1806 to July Week 1 2020
Search Strategy:

#	Searches
1	exp Headache/
2	(headache* or head ache*).ti,ab,id.
3	or/1-2
4	Health education/
5	exp Client Education/
6	exp Psychoeducation/
7	Programmed Instruction/
8	Computer Assisted Instruction/
9	Individualized Instruction/
10	Distance Education/
11	exp Educational Programs/
12	Hot Line Services/
13	exp Counseling/
14	exp Self-Help Techniques/
15	(psychoeducat* or psycho-educat*).ti,ab,id.
16	((information* or educat* or guid* or counsel?ing or school* or Course* or Coach* or instruction or class* or advic* or advis* or handout* or hand-out* or pamphlet* or leaflet* or booklet* or brochure* or website) adj3 (health or headache og migraine or treatment* or therapy or intervention or management or patient* or tailor* or individual* or Patient-centred or Patient-centered or Patient-oriented or Patient-focused or Patient-based or Patient-tailored or client-cent* or client cent* or client-focus* or client focus* or Client-oriented or Client-based or Client-tailored or person-centered or person-centred or person centered or person centred or person-focus* or person focus* or Person-oriented or Person-based or Person-tailored or Individual-centred or Individual-centered or Individual-focused or Individual-oriented or Individual-based or Individual-tailored)).ti,ab,id.
17	(self-management or self-instruction* or selfmanagement or selfinstruction* or self management or self instruction* or self-guid* or self guid* or selfguid* or self care or self-care or selfcare or guidance).ti,ab,id.
18	((information* or instruction* or educat*) adj3 (material or advic* or advis* or handout* or hand-out* or pamphlet* or leaflet* or booklet* or brochure* or guid* or resource*)).ti,ab,id.
19	((education* or learn* or training or teach*) adj2 (program* or patient* or consumer* or material* or resource* or aid*)).ti,ab,id.

20	(patient adj (information or knowledge or website*)).ti,ab,id.
21	(workshop* or seminar* or ((discussion or support) adj group*)).ti,ab,id.
22	(factsheet* or advice line* or advice-line* or hotline or help line* or help-line* or helpline*).ti,ab,id.
23	((tele* or tele-based or tele-support* or telehealth or tele-health or telemedicine or tele-medicine or internet* or internet-based or internetbased or internet-delivered or internet-support* or internet-assisted or web or webbased or web-based or web based or web-support* or web-assisted or WWW or email* or e-mail or telephone or phone or computer* or computer-based or computerbased or computer-support* or computer-assisted or PC or PCs or PC-based or pc-support* or pc-assisted or skype or skype-based or skype-delivered or skype-assisted or app*1 or app-based or app based or app-assisted or smartphone* or ipad* or tablet* or IOS or android* or online* or on-line or mobile or ehealth or e-health or e-therap* or distance or remote or video-conferenc* or videoconference* or chatroom* or electronic or digital* or technology or technology-based or technology-delivered or technology-supported or technology-assisted or distance) adj3 (counsel* or support* or guide* or guidance or education or school* or course* or coach* or teach* or session* or information)).ti,ab,id.
24	(tele-counsel?ing or tele-education or tele-instruction or tele-session* or tele-class* or tele-course* or tele-guid* or tele-coaching or internet-counsel?ing or internet-education or internet-instruction or internet-session* or internet-class* or internet-course* or internet-coaching or internet-guid* or web-counsel?ing or web-education or web-instruction or web-session* or web-class* or web-course* or web-coaching or web-guid* or e-counsel?ing).ti,ab,id.
25	or/4-24
26	3 and 25
27	(((random* or cluster-random* or quasi-random* or control?ed or crossover or cross-over or blind* or mask*) adj4 (trial*1 or study or studies or analy*)) or rct).ti,ab,id.
28	(placebo* or single-blind* or double-blind* or triple-blind* or ((single or double or triple) adj2 (blind* or mask*))).ti,ab,id.
29	((patient* or person* or participant* or population* or allocate* or assign*) adj3 (random* or blind* or mask*)).ti,ab,id.
30	or/27-29
31	26 and 30
32	limit 31 to (yr="2004-2020" and (english or danish or norwegian or swedish))

Cochrane Central

ID	Search	Hits
#1	MeSH descriptor: [Headache Disorders, Primary] this term only	
#2	MeSH descriptor: [Headache] this term only	
#3	MeSH descriptor: [Headache Disorders] this term only	
#4		
#5	MeSH descriptor: [Tension-Type Headache] explode all trees	
#6	(headache* or head ache*):ti,kw	
#7	#1 or #2 or #3 or #4 or #5 or #6	
#8	MeSH descriptor: [Health Education] this term only	
#9	MeSH descriptor: [Patient Education as Topic] explode all trees	
#10	MeSH descriptor: [Programmed Instructions as Topic] explode all trees	
#11	MeSH descriptor: [Hotlines] explode all trees	
#12	MeSH descriptor: [Counseling] explode all trees	
#13	(psychoeducat* or psycho-educat*):ti,ab,kw	
#14	((information* or educat* or guid* or counsel?ing or school* or Course* or Coach* or instruction or class* or advic* or advis* or handout* or hand-out* or pamphlet* or leaflet* or booklet* or brochure* or website) NEAR/3 (health or headache og migraine or treatment* or therapy or intervention or management or patient* or tailor* or individual* or Patient-centred or Patient-centered or Patient-oriented or Patient-focused or Patient-based or Patient-tailored or client-cent* or client cent* or client-focus* or client focus* or Client-oriented or Client-based or Client-tailored or person-centered or person-centred or person centered or person centred or person-focus* or person focus* or Person-oriented or Person-based or Person-tailored or Individual-centred or Individual-centered or Individual-focused or Individual-oriented or Individual-based or Individual-tailored)):ti,ab,kw	
#15	(self-management or self-instruction* or selfmanagement or selfinstruction* or self management or self instruction* or self-guid* or self guid* or selfguid*):ti,kw	
#16	((information* or instruction* or educat*) NEAR/3 (material or advic* or advis* or handout* or hand-out* or pamphlet* or leaflet* or booklet* or brochure* or guid* or resource* or e-book*)):ti,ab,kw	
#17	((education* or learn* or training or teach*) NEAR/2 (program* or patient* or consumer* or material* or resource* or aid*)):ti,ab,kw	
#18	(patient NEAR/1 (information or knowledge or website*)):ti,ab,kw	
#19	(workshop* or seminar* or ((discussion or support) NEAR/1 group*)):ti,ab,kw	
#20	(factsheet* or fact sheet* or fact-sheet* or advice line* or advice-line* or hotline or help line* or help-line* or helpline*):ti,ab,kw	
#21	((tele* or tele-based or tele-support* or telehealth or tele-health or telemedicine or tele-medicine or internet* or internet-based or internetbased or internet-delivered or internet-support* or internet-assisted or web or webbased or web-based or web based or web-support* or web-assisted or WWW or email* or e-mail or telephone or phone or computer* or computer-based or computerbased or computer-support* or computer-assisted or PC or PCs or PC-based or pc-support* or pc-assisted or skype or skype-based or skype-delivered or skype-assisted or app or apps or app-based or app based or app-assisted or smartphone* or ipad* or tablet* or IOS or android* or online* or on-line or mobile or ehealth or e-health or e-therap* or distance or remote or video-conferenc* or videoconference* or chatroom* or electronic or digital* or technology or technology-based or technology-delivered or technology-supported or technology-assisted or distance) NEAR/3 (counsel* or support* or guide* or guidance or education or school* or course* or coach* or teach* or session* or information)):ti,kw	
#22	(counsel*ing or tele-counsel*ing or tele-education or tele-instruction or tele-session* or tele-class* or tele-course* or tele-guid* or tele-coaching or internet-counsel*ing or internet-	

	education or internet-instruction or internet-session* or internet-class* or internet-course* or internet-coaching or internet-guid* or web-counsel*ing or web-education or web-instruction or web-session* or web-class* or web-course* or web-coaching or web-guid* or e-counsel*ing):ti,kw
#23	#8 or #9 or #10 or #11 or #12 or #13 or #14 or #15 or #16 or #17 or #18 or #19 or #20 or #21 or #22
#24	#7 AND #23 with Publication Year from 2004 to 2020, in Trials

Cinahl

#	Query	Limiters/Expanders
S27	S21 AND S26	Limiters - Published Date: 20040101-20201231; Language: Danish, English, Norwegian, Swedish
S26	S22 OR S23 OR S24 OR S25	
S25	((patient* or person* or participant* or population* or allocate* or assign*) N3 (random* or blind* or mask*))	
S24	(placebo* or single-blind* or double-blind* or triple-blind* or ((single or double or triple) N1 (blind* or mask*))	
S23	(((random* or cluster-random* or quasi-random* or control#ed or crossover or cross-over or blind* or mask*) N4 (trial* or study or studies or analy*)) or rct)	
S22	PT Randomized Controlled Trial OR MH "Ranndomized Controlled Trials+"	
S21	S4 and S20	
S20	S5 OR S6 OR S7 OR S8 OR S9 OR S10 OR S11 OR S12 OR S13 OR S14 OR S15 OR S16 OR S17 OR S18 OR S19	
S19	(tele-counsel#ing or tele-education or tele-instruction or tele-session* or tele-class* or tele-course* or tele-guid* or tele-coaching or internet-counsel#ing or internet-education or internet-instruction or internet-session* or internet-class* or internet-course* or internet-coaching or internet-guid* or web-counsel#ing or web-education or web-instruction or web-session* or web-class* or web-course* or web-coaching or web-guid* or e-counsel#ing)	
S18	((tele* or tele-based or tele-support* or telehealth or tele-health or telemedicine or tele-medicine or internet* or internet-based or internetbased or internet-delivered or internet-support* or internet-assisted or web or webbased or web-based or web based or web-support* or web-assisted or WWW or email* or e-mail or telephone or phone	

	or computer* or computer-based or computerbased or computer-support* or computer-assisted or PC or PCs or PC-based or pc-support* or pc-assisted or skype or skype-based or skype-delivered or skype-assisted or apps or app-based or app based or app-assisted or smartphone* or ipad* or tablet* or IOS or android* or online* or on-line or mobile or ehealth or e-health or e-therap* or distance or remote or video-conferenc* or videoconference* or chatroom* or electronic or digital* or technology or technology-based or technology-delivered or technology-supported or technology-assisted or distance) N3 (counsel* or support* or guide* or guidance or education or school* or course* or coach* or teach* or session* or information))	
S17	(factsheet* or fact sheet* or advice line* or advice-line* or hotline or help line* or help-line* or helpline*)	
S16	(workshop* or seminar* or ((discussion or support) N1 group*))	
S15	(patient N1 (information or knowledge or website*))	
S14	((education* or learn* or training or teach*) N2 (program* or patient* or consumer* or material* or resource* or aid*))	
S13	((information* or instruction* or educat*) N3 (material or advic* or advis* or handout* or hand-out* or pamphlet* or leaflet* or booklet* or brochure* or guid* or resource*))	
S12	(self-management or self-instruction* or selfmanagement or selfinstruction* or self management or self instruction* or self-guid* or self guid* or selfguid* or self care or self-care or selfcare or guidance)	
S11	((information* or educat* or guid* or counsel#ing or school* or Course* or Coach* or instruction or class* or advic* or advis* or handout* or hand-out* or pamphlet* or leaflet* or booklet* or brochure* or website) N3 (health or headache og migraine or treatment* or therapy or intervention or management or patient* or tailor* or individual* or Patient-centred or Patient-centered or Patient-oriented or Patient-focused or Patient-based or Patient-tailored or client-cent* or client cent* or client-focus* or client focus* or Client-oriented or Client-based or Client-tailored or person-centered or person-centred or person centered or person centred or person-focus* or person focus* or Person-oriented or Person-based or Person-tailored or Individual-centred or Individual-centered or Individual-focused or Individual-oriented or Individual-based or Individual-tailored))	
S10	(MH "Psychoeducation")	
S9	(psychoeducat* or psycho-educat*)	

S8	(MH "Counseling") OR (MH "Anticipatory Guidance") OR (MH "Motivational Interviewing") OR (MH "Peer Counseling")	
S7	(MH "Telephone Information Services")	
S6	(MH "Programmed Instruction+")	
S5	MH ("Health education" or "Patient Education" or "Patient Discharge Education" or "Psychoeducation")	
S4	S1 OR S2 OR S3	
S3	SU (headache* or head ache*)	
S2	(headache* or head ache*)	
S1	MH "Headache" or MH "Headache, Primary" or MH "Tension headache"	

PEDRO

Abstract & Title: headache*
Therapy: Education
Method: Clinical trial
Published since: 2004-20

OT Seeker

Title/Abstract: headache* or migraine*
Method: Randomised Controlled Trial
Year published: 2004-2020
